# Eschenmoser's salts: from design to applications in organic synthesis

**DOI:** 10.1039/d6qo00713a

**Published:** 2026-09-15

**Authors:** Margherita Miele, Eisuke I. Comas Iwasita, Andrea D'Annibale, Andrés R. Alcántara, Laura Castoldi, Vittorio Pace

**Affiliations:** a University of Turin, Department of Chemistry Via Giuria 7 10125 Turin Italy; b University of Rome “La Sapienza”, Department of Chemistry P.le A. Moro 5 00185 Rome Italy vittorio.pace@uniroma1.it; c Complutense University of Madrid, Department of Chemistry in Pharmaceutical Sciences P.za A. Ramón y Cajal s/n 28040 Madrid Spain; d University of Milan, Department of Pharmaceutical Sciences, General and Organic Chemistry Section “A. Marchesini” Via Venezian 21 20133 Milan Italy laura.castoldi@unimi.it; e University of Vienna, Department of Pharmaceutical Sciences, Division of Pharmaceutical Chemistry Josef-Holaubek-Platz 2 1090 Vienna Austria vittorio.pace@univie.ac.at

## Abstract

For over half a century, Eschenmoser's salt and related analogues have been widely exploited in organic synthesis, enabling the aminomethylation and α-methylenation of a broad range of substrates. Owing to their tunable structure and counterions, these salts exhibit remarkable versatility in homologation, C–C bond forming reactions, C–H functionalization, and in the synthesis of heterocycles, natural products, and biologically active compounds. This review highlights the structural modifications, reactivity, and applications of Eschenmoser-type iminium salts, emphasizing their continuing impact and adaptability in modern synthetic methodology.

## Abstract 2

This review would provide a comprehensive perspective on Eschenmoser's salts as highly versatile releasing agents for introducing the ubiquitous aminomethyl fragment in organic arrays.

## Graphical abstract

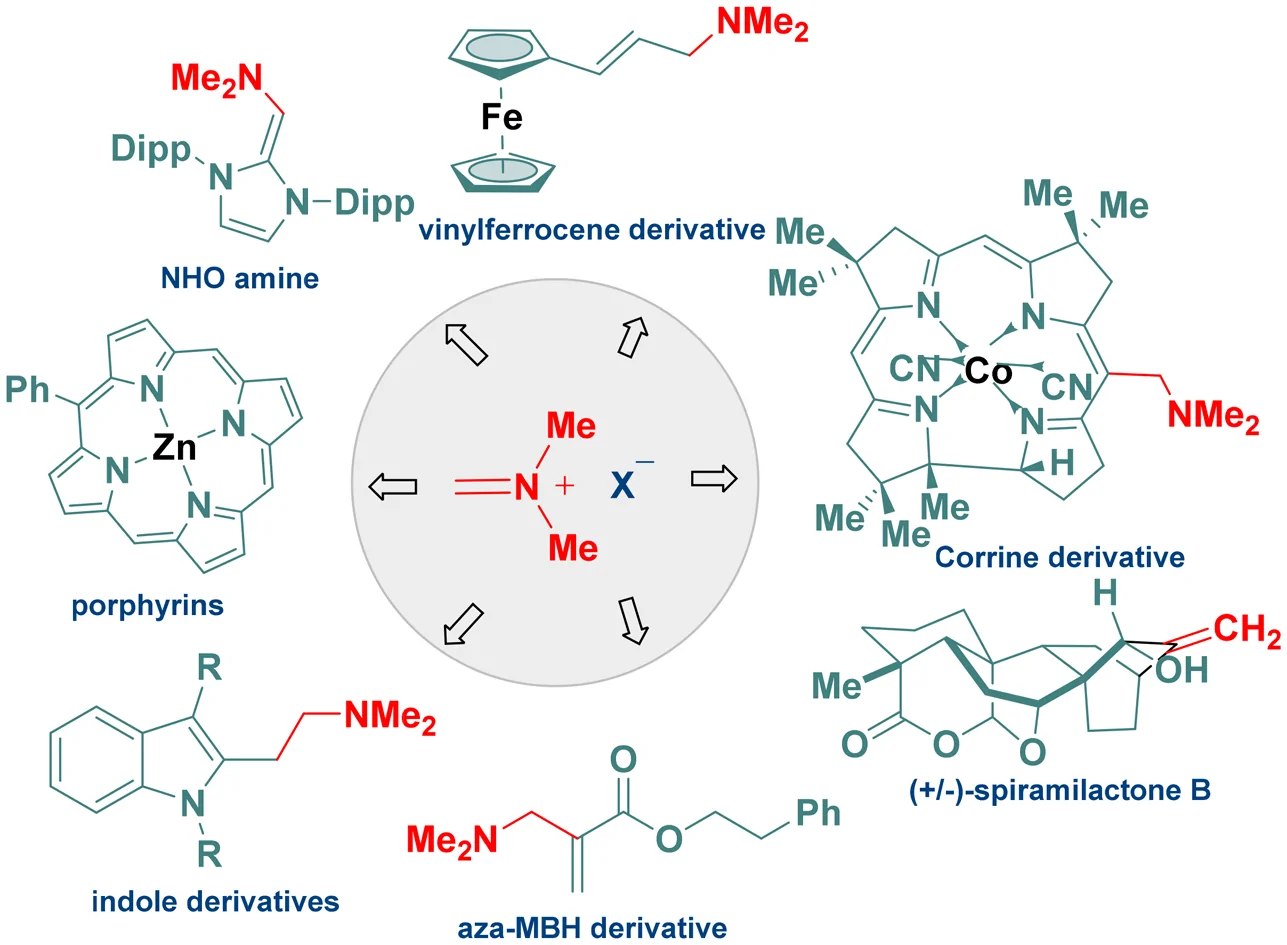

## 1. Introduction

**Scheme 1:**
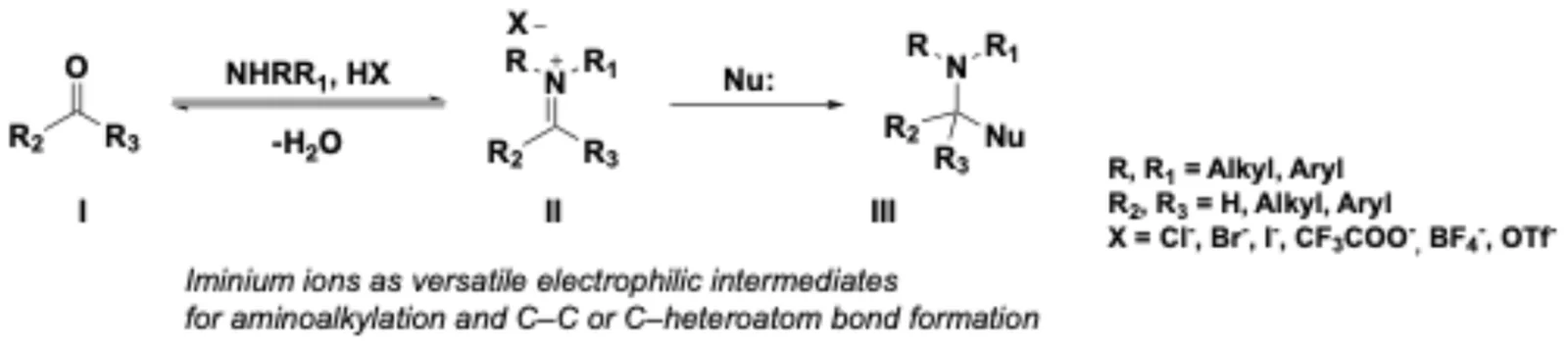
Electronic structure and general nucleophilic reactivity of iminium ions.

Nitrogen-containing compounds are widespread in natural products, pharmaceuticals, agrochemicals, and functional materials, making the construction of C–N-containing frameworks a major goal in organic synthesis. Within this context, imines and iminium ions represent particularly valuable intermediates because they combine the presence of a nitrogen functionality with activation of the adjacent carbon center. Iminium ions are characterized by a strongly polarized C=N^+^ bond, in which the positive charge is delocalized between the nitrogen and carbon, thereby imparting pronounced electrophilic character to the iminium carbon.^1,2^ Although neutral imines may be less electrophilic than their parent carbonyl compounds, conversion into iminium salts increases the electrophilicity of the C=N carbon and facilitates nucleophilic attack (Scheme 1).^3–5^

**Scheme 2:**
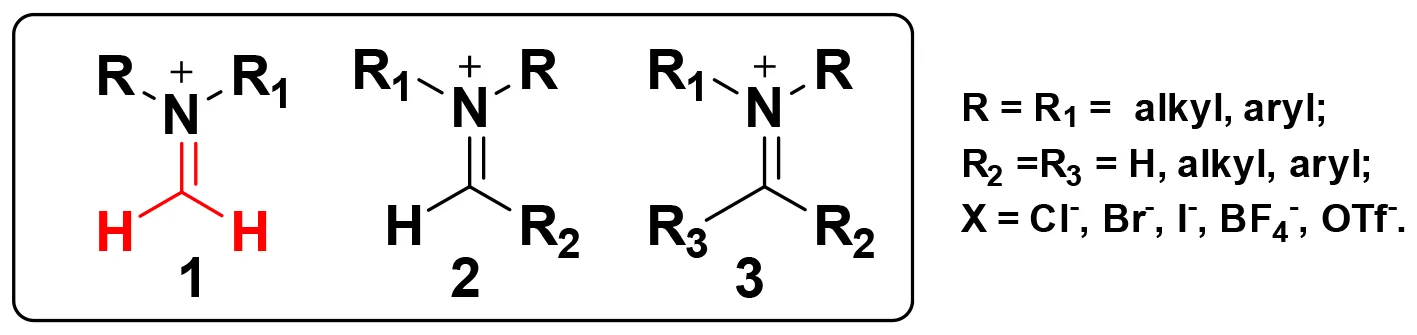
Iminium salts derived from formaldehyde, aldehydes, and ketones.

Iminium salts can be broadly classified according to the carbonyl compound from which they are derived as methaniminium salts 1, derived from formaldehyde; aldiminium or ternary iminium salts 2, derived from aldehydes; and ketiminium or quaternary iminium salts 3, derived from ketones (Scheme 2).^6^

The pronounced electrophilicity of iminium salts enables their application in a wide range of transformations, including condensation reactions with carbonyl and aromatic compounds, macrocyclization reactions involving electron-rich heteroarenes,^7^ Mannich-type reactions,^8^ Morita–Baylis–Hillman (MBH)-type reactions and their aza-variants, as well as C–H functionalization which has attracted significant attention in drug discovery.^8,9^

Many iminium ions are short-lived species that must be generated *in situ*, and their concentration, lifetime and reactivity can depend strongly on the precursor structure, acidity, solvent, counterion, and reaction conditions. Preformed iminium salts provide a practical alternative, as they are structurally defined electrophiles that can be used directly for aminoalkyl group transfer.^10,11^

Depending on their structure and counterion, preformed iminium salts may exhibit useful solubility in polar aprotic solvents, enabling their use with highly reactive nucleophiles. They readily react with active methylene compounds, organometallic reagents, and electron-rich or heteroaromatics to afford functionalized amines.^12,13^ Iminium salts can also participate as electrophilic partners in C–C bond forming reactions with organolithium, organomagnesium and organocopper reagents, as well as with electron-rich systems such as enamines, indoles, and amino- or hydroxyl-arenes.^14^ Additionally, their versatility further extends to the synthesis of heterocyclic ring systems.^12^

**Scheme 3:**
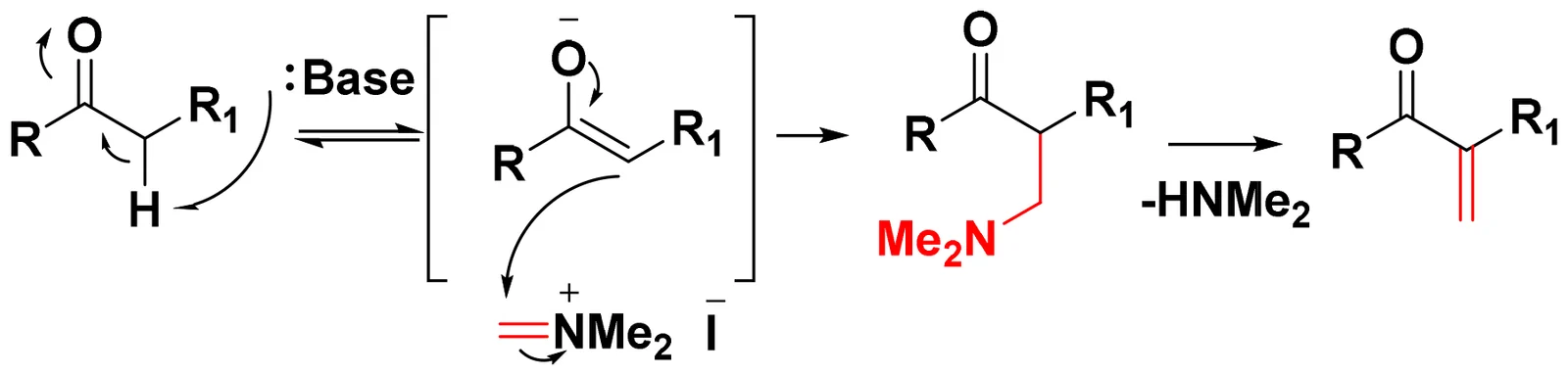
Typical Eschenmoser methenylation.

Among methaniminium salts, dimethyl(methylene)ammonium salts are particularly prominent. A prototypical example is Eschenmoser's salt, dimethyl(methylene)ammonium iodide, [(Me_2_N=CH_2_)^+^I^−^] S1.^15^ This reagent acts as an electrophilic aminomethylating agent toward carbon- and heteroatom-centred nucleophiles and can also serve as a one-carbon synthon for introducing an *exo*-methylene group at the α-position of carbonyl compounds. This transformation, known as Eschenmoser methenylation,^16^ proceeds through initial aminomethylation, followed by quaternization of the dimethylamino group and subsequent elimination, ultimately yielding the corresponding α-methylene carbonyl compound (Scheme 3).

**Scheme 4:**
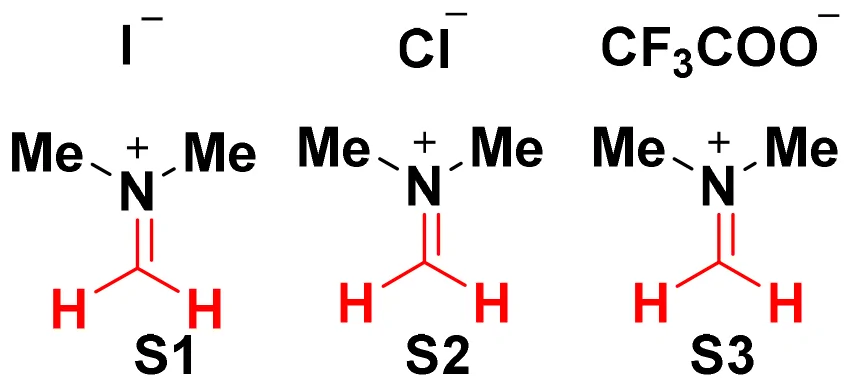
Dimethyl(methylene)ammonium salts with different counterions.

Alternative counterions, such as chloride [(Me_2_N=CH_2_)^+^Cl^−^] S2,^17^ first prepared by Böhme and popularized by Kinast and Tietze,^18^ and trifluoroacetate S3 introduced by Potier *et al.*^19^ (Scheme 4), have been widely applied in recent years. These compounds are functionally related Mannich-type aminomethylating reagents. Selection of an appropriate counterion should consider solubility, ease of purification, and moisture sensitivity to ensure optimal reaction outcomes.

Generally, the reactivity of iminium salts toward nucleophiles decreases in the order R_2_ = H > aryl > alkyl. More highly substituted iminium salts (R_2_ ≠ H) are, therefore, less frequently employed in Mannich reactions, although their substitution pattern may offer improved regio- and stereochemical control. Differences in reactivity among counterions are mainly attributed to variation in solubility; thus, the use of aprotic solvents (*e.g.* MeCN, DMF, CH_2_Cl_2_) can improve reaction rates and overall efficiency. In a comparison study, Lorenz *et al.*^20^ analyzed the different solubility for the three salts by reacting them with silyl enol ethers, the reaction times were kept identical for all the reagents, and the product resulted from the addition of the salt to the nucleophilic substrate. In DMF, all three salts exhibited high solubility and broadly comparable reactivity. In less polar solvents, however, the differences became more pronounced, with the trifluoroacetate derivative exhibiting greater solubility and, consequently, higher reaction efficiency.

While counterions may influence solubility and reaction kinetics, they generally have minimal impact on stereochemistry, with certain exceptions in aminomethylation reactions. However, some counterions, such as iodide, can promote undesired side reactions.^21^

This review focuses on the modifications, applications, and reactivity of methaniminium salts, with particular emphasis on Eschenmoser's salt and closely related derivatives, highlighting their versatility as electrophilic reagents across classical and contemporary applications in organic synthesis.

## 2. Types of iminium salts

### 2.1. Dimethyl(methylene)ammonium iodide (S1)

Dimethyl(methylene)ammonium iodide S1 (Scheme 4) is a hygroscopic solid and should therefore be stored and handled under dry conditions.^22^ Although S1 is readily soluble in highly polar aprotic solvents such as DMF, its solubility decreases significantly in less polar media. In CH_2_Cl_2_, lower conversions may be observed compared with the trifluoroacetate analogue, whereas in diethyl ether, the salt is essentially insoluble and no reaction is generally detected. Despite these limitations, S1 remains one of the most widely used dimethyl(methylene)ammonium reagents because of its established preparation, broad synthetic applicability, and generally reliable performance.^20^

**Scheme 5:**
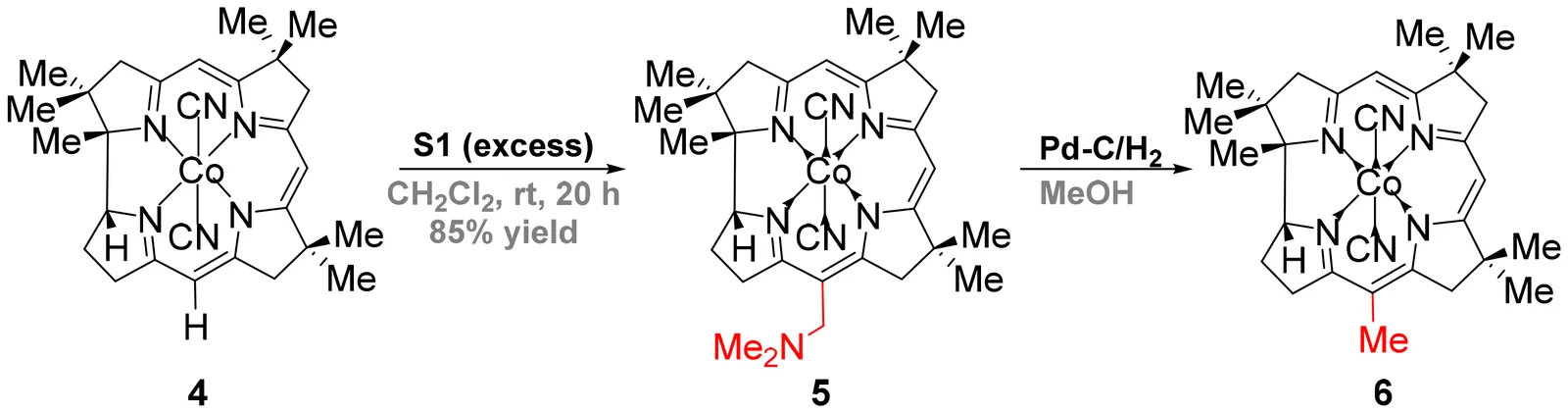
Synthesis of the corrine macrocycle by Eschenmoser *et al.*

During the synthesis of vitamin B_12_,^23^ the lack of an efficient methodology to introduce a methyl group at the *meso* positions of the macrocyclic and complex substrate corrin chromophore prompted the development of a reactive species capable of effecting this transformation. Eschenmoser *et al.*^15^ reported for the first time the synthesis and use of dimethyl(methylene)ammonium iodide (S), now known as Eschenmoser's salt. As depicted in Scheme 5, a solution of racemic dicyanocobalt(iii)-1,2,2,7,7,12,12-heptamethylcorrin 4 in CH_2_Cl_2_, containing an excess of S1, was stirred at room temperature for 20 h, affording the 15-(dimethylamino)methyl derivative 5 in 85% yield as a pure crystalline solid *via* a smooth electrophilic substitution. Subsequent hydrogenolysis of 5 furnished the corresponding 15-methylcorrin derivative 6. This sequence (4 → 6) provided a straightforward and efficient method for methylating sterically unhindered *meso* positions of the corrin system.

**Scheme 6:**
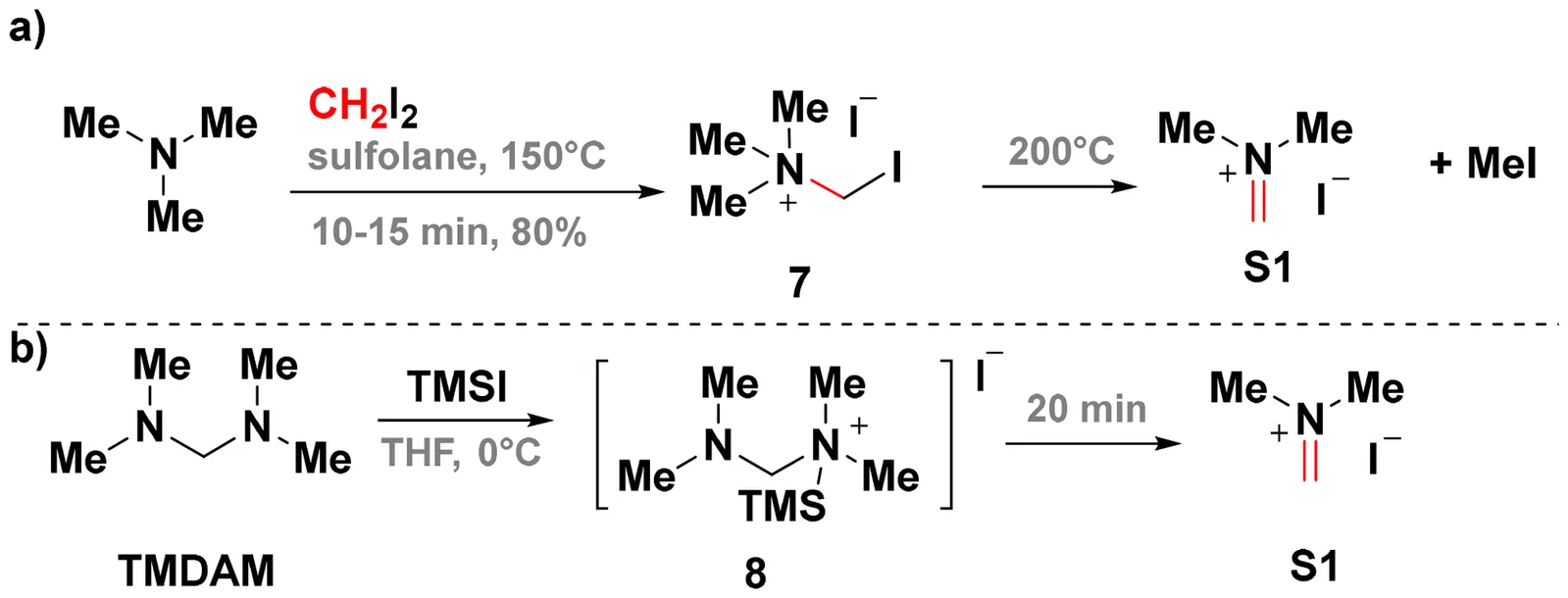
Eschenmoser's salt synthesis starting from trimethylamine or TMDAM.

Despite the elegance of the first synthesis of S1 (Scheme 6a),^15^ from trimethylamine and diiodomethane in tetrahydrothiophene dioxide (sulfolane), Bryson *et al.*^24^ reported in 1980 a methodology to make the salt synthesis shorter (Scheme 6b). Thus, by treating amino tetramethylmethylenediamine (TMDAM) with trimethylsilyl iodide (TMSI), the subsequent cleavage of 8 led to the desired iminium salt S1 in 96% yield.

**Scheme 7:**
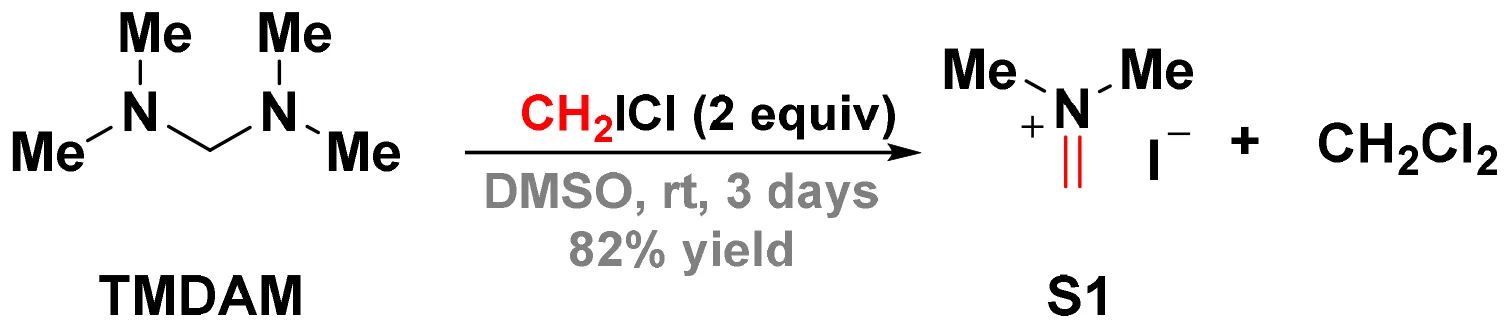
Eschenmoser's salt synthesis by Miyano *et al.*

In 1982, Miyano *et al.*^25^ prepared Eschenmoser's salt S1 (Scheme 7) from TMDAM and chloroiodomethane, while the multiple attempts to build the salt from other dihalomethanes, such as diiodo-, dibromo- and dichloromethane, led to less efficient yields.

### 2.2. Dimethyl(methylene)ammonium chloride (S2)

Dimethyl(methylene)ammonium chloride S2 is considered highly hygroscopic and requires careful exclusion of moisture during storage and manipulation.^17^ Its major practical limitation is its relatively low solubility in many commonly employed organic solvents. Although S2 dissolves effectively in highly polar aprotic media such as DMF, its solubility is reduced in CH_2_Cl_2_ and negligible in diethyl ether. These solubility constraints may limit its apparent reactivity under heterogeneous conditions, even though the intrinsic electrophilic behavior of the methaniminium cation is expected to remain closely related to that of the iodide and trifluoroacetate salts.^20^

**Scheme 8:**
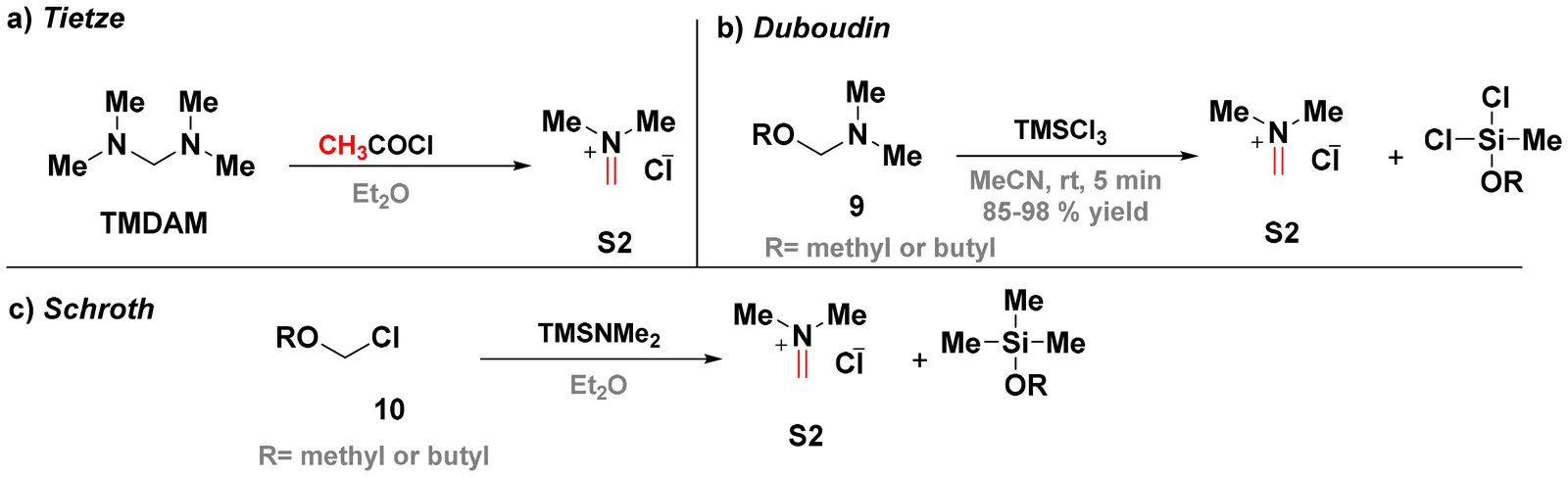
Different synthetic routes for the production of S2.

Following Böhme's first preparation in 1957,^17^ Tietze *et al.*^18^ proposed an alternative synthesis in which aminal TMDAM was reacted with acetyl chloride to afford salt S2 (Scheme 8a). Subsequently, Duboudin *et al.*^26^ reported in 1986 an elegant alternative method aiming to overcome limitations such as long reaction times and the use of expensive or hazardous reagents.^15,19,24,25,27^ Their strategy (Scheme 8b) employed geminal aminoether 9 which reacted with methyltrichlorosilane in acetonitrile at room temperature in just five minutes. Key advantages of this approach include standard and mild conditions, short reaction times, stoichiometric reagent usage, and the employment of TMSCl, which was an industrial by-product. Some years later, in 1993, Schroth's group^28^ described a quantitative preparation of Böhme's salt S2*via* an S_N_2 reaction between α-chloroether 10 and *N*,*N*-dimethyltrimethylsilylamine (Scheme 8c).

### 2.3. Dimethyl(methylene)ammonium trifluoroacetate (S3)

Dimethyl(methylene)ammonium trifluoroacetate S3 differs from the iodide and chloride analogues in being a liquid and generally more soluble in less polar organic solvents. This feature facilitates both homogeneous reaction conditions and convenient transfer by a syringe. In CH_2_Cl_2_, S3 can provide substantially higher conversions than S1 and S2, consistent with its greater solubility in this medium. Even in diethyl ether, where the iodide and chloride salts are insoluble, S3 retains limited solubility and may still afford modest product yields. Its preparation is, however, more demanding, because residual trifluoroacetic acid must be removed carefully, typically by distillation,^18^ to avoid reduced yields in Mannich-type reactions. Despite this additional purification step, S3 may be advantageous when homogeneous conditions in less polar solvents are required.^20^

**Scheme 9:**
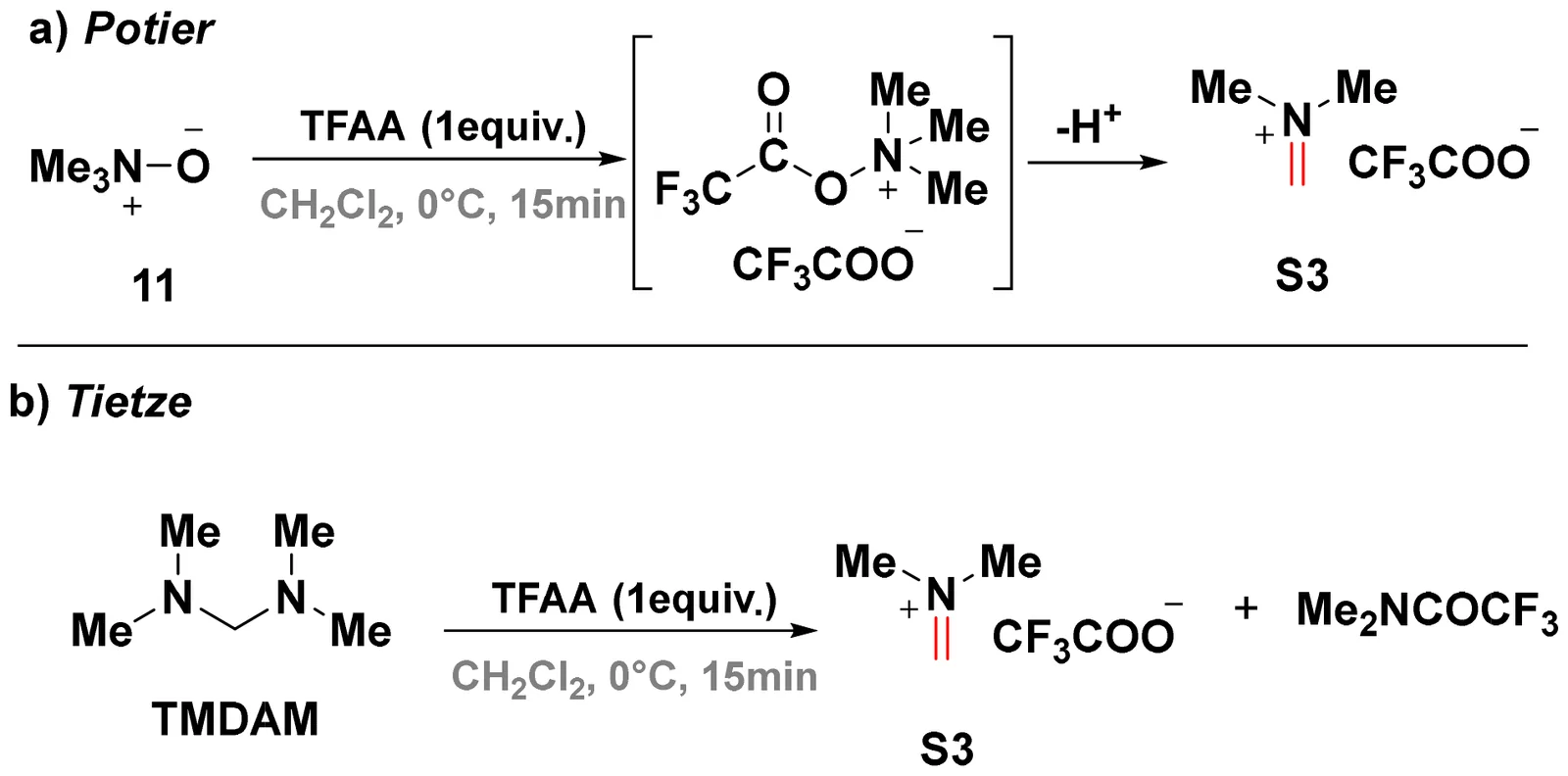
Different synthetic routes for the production of S3.

The first preparation of dimethyl(methylene)ammonium trifluoroacetate S3 was reported in 1968, when Potier *et al.*^19^ proposed the reaction between trimethylamine *N*-oxide 11 and trifluoroacetic anhydride (TFAA) in CH_2_Cl_2_ (Scheme 9a). Later, Tietze *et al.*^18^ reported a more frequently employed alternative procedure in which the salt S3 is obtained *via* the reaction of TMDAM and trifluoroacetic acid (TFA) (Scheme 9b).

The dimethyl(methylene)ammonium trifluoroacetate S3 – also known as Potier–Tietze's iminium salt – has a broad range of applications, in particular, in the field of organometallic chemistry; to the best of our knowledge, S3 is the only salt used for homologative amination reactions with organozinc reagents.^29–32^

## 3. Elimination of the dimethylamino group

Eschenmoser's salt is widely employed for introducing a methylene group at the α-position of various substrates. The reaction proceeds through quaternarization of an *N*,*N*-dimethyl Mannich base followed by elimination of the dimethylamino functionality. Among the available methodologies, four main strategies are commonly used to obtain the product contrary to Zaitsev's rule (*i.e.*, avoiding the formation of the most substituted alkene):

1. conversion to the corresponding quaternary ammonium salt (Hofmann's elimination);^33,34^

2. conversion to the corresponding *N*-oxide (Cope's elimination);^35–39^

3. use of a base and

4. simple agitation at room or lower temperatures.

**Scheme 10:**
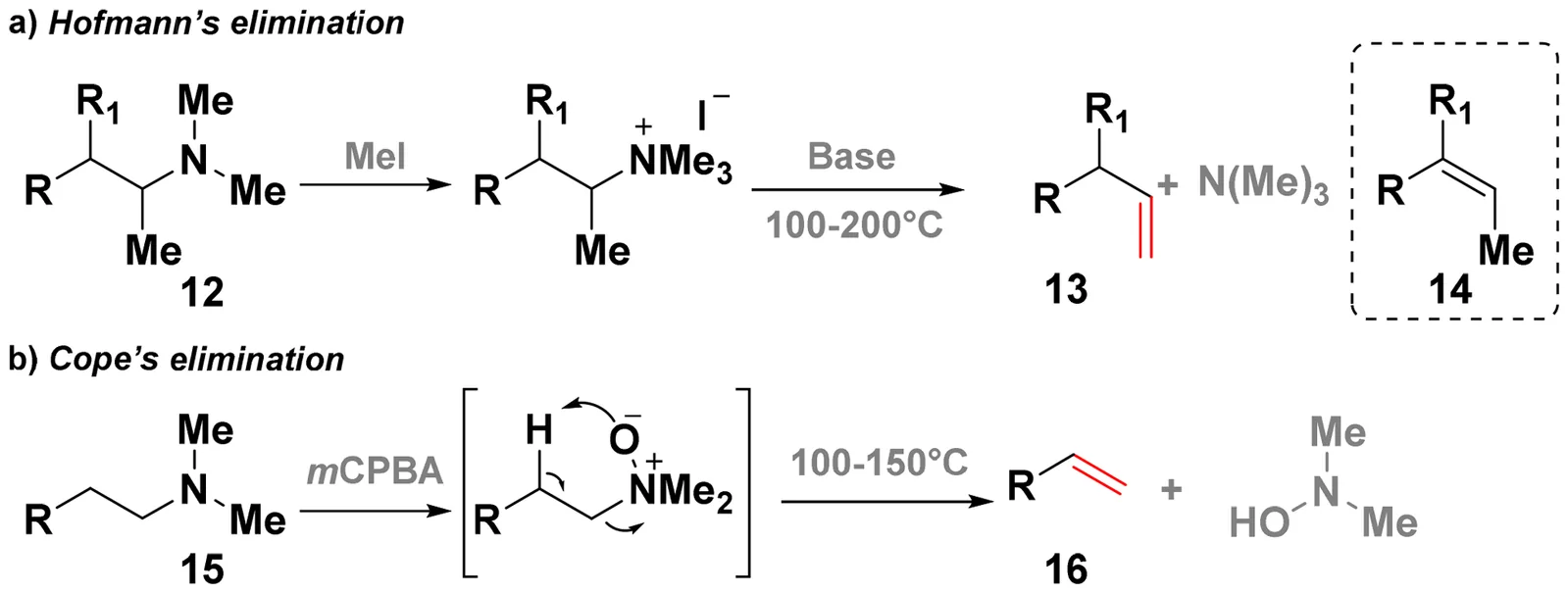
Methods for the elimination of the dimethylamino function.

The first method, Hofmann's elimination (Scheme 10a), involves exhaustive methylation of tertiary amine 12 with an excess of methyl iodide (MeI), followed by treatment with a strong base (*e.g.* Ag_2_O, DBU, Al_2_O_3_). Thereby, the generated quaternary ammonium salt undergoes elimination upon heating in the presence of the base (B^−^) and releases trimethylamine with the formation of a terminal olefin.^40–53^ Notably, taking into consideration the regioselectivity, the reaction generates the least substituted olefin (Hofmann's product 13) rather than the more substituted Zaitsev's product 14. This preference arises from the presence of the very bulky leaving group which favors abstraction of the proton from the primary carbon during elimination.

The so-called Cope's elimination consists of oxidizing the tertiary amino function to an *N*-oxide intermediate, which undergoes, after heating, intramolecular elimination, affording the desired terminal olefin.^54–57^ As shown in Scheme 10b, the tertiary amine 15 is reacted with *meta*-chloroperoxybenzoic acid (*m*-CPBA) which, by a *syn*-periplanar elimination, produces olefin 16 and hydroxylamine. In most cases the deprotonation proceeds according to Hofmann's rules since the *N*-oxide function preferentially reacts with the more accessible hydrogens, so it consequently leads to the less substituted olefin.^58^

The use of a base^59–61^ and stirring at different temperatures^62–68^ represent other techniques often employed. Usually, the reaction occurs at room temperature and only scarcely at lower temperatures.

However, these procedures typically involve a three-step sequence, which can be both laborious and time consuming (*e.g.* approximately 24 h for Hofmann's elimination). Consequently, the development of a more direct and rapid method for converting a dimethylamino group into a more reactive leaving group would enable a facile elimination enhancing Eschenmoser's methylenation in terms of both efficiency and operational simplicity.

In this context, Yamada *et al.*^69^ developed a rapid methodology for the cleavage of the dimethylamino group using 2-chloro-4,6-dimethoxy-1,3,5-triazine (CDMT). The reaction proceeds *via* the formation of a triazinylammonium intermediate, which readily undergoes elimination due to the strong electron-withdrawing nature of the triazinyl group.^70^

**Scheme 11:**
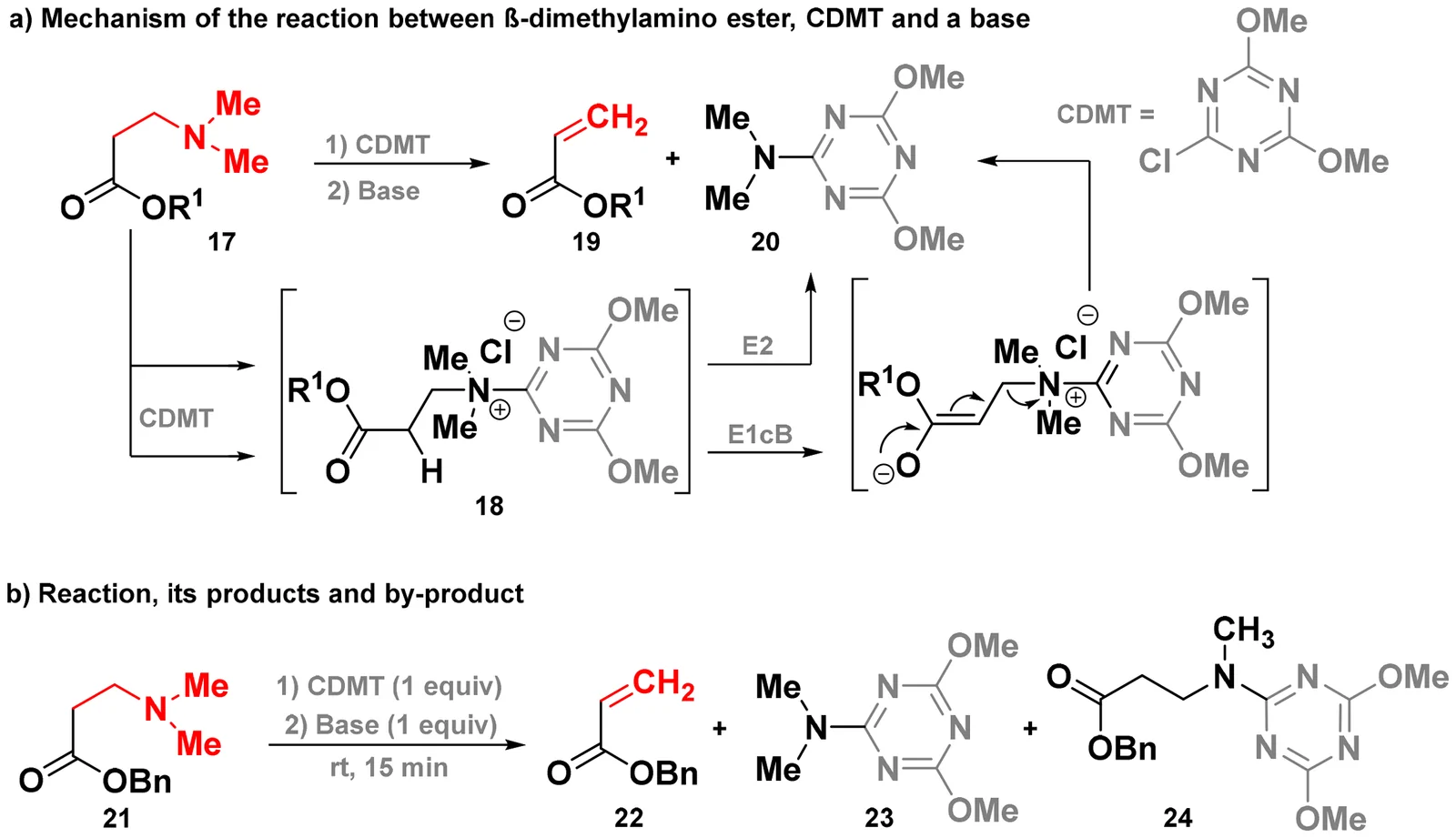
Removal of the dimethylamino function.

The treatment of β-dimethylamino ester 17 with CDMT affords the triazinylammonium salt 18 which upon subsequent E2 or E1cB elimination yields the desired α,β-unsaturated ester 19 along with the formation of the co-product 20 (Scheme 11a).

Optimization studies (Scheme 11b) revealed that the reaction of benzyl 3-(dimethylamino) propionate (21) with CDMT and triethylamine (Et_3_N) in CH_2_Cl_2_ at room temperature for 15 min provided 22 in 84% yield. The use of DMF, MeCN, and 2-PrOH improved yields up to 94%, 89%, and 85%, respectively, whereas MeOH and THF afforded lower yields (74–75%) due to product decomposition, most probably *via* the possible 1,4-addition or polymerization.

**Scheme 12:**
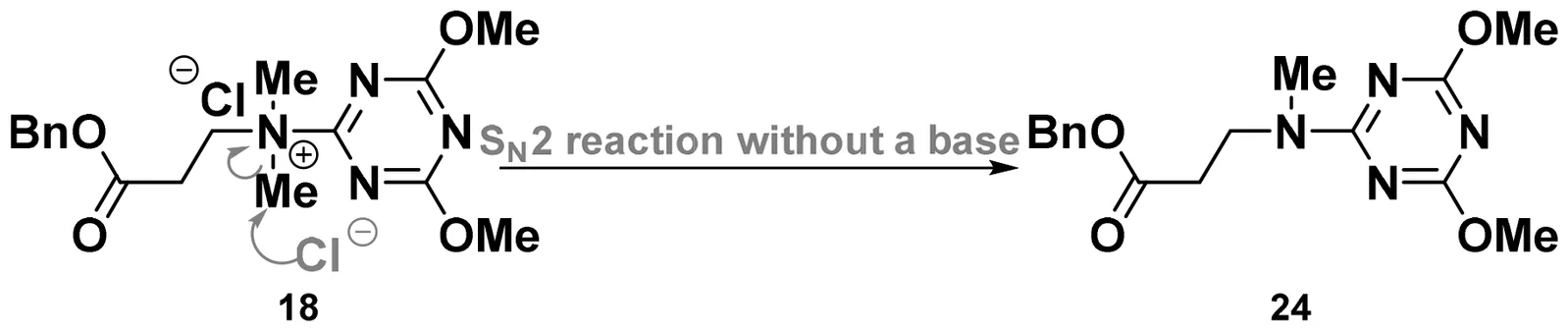
Competitive demethylation of the amino moiety.

Base screening in DMF showed that DBU, K_2_CO_3_, and 4-methylmorpholine led to moderate yields (53–80%), while pyridine or base-free conditions produced poor yields and the demethylated by-product 24 (Scheme 12). The formation of 24 likely results from an S_N_2 attack of chloride on the methyl group of the triazinylammonium intermediate since deprotonation at the α-position of the carbonyl group hardly proceeds under neutral or weakly basic conditions.

Substrate variation (Table 1) did not affect the outcome of the process except for α-methyl-substituted compounds, where steric hindrance of the α-proton promoted competitive demethylation (26b, 50% yield, entry 1). Switching the solvent to 2-PrOH mitigated this issue improving the yield up to 90%. The method also afforded acrylate 26e in 93% yield (entry 4), in contrast to only 26% yield obtained *via* the conventional MeI/NaHCO_3_ route. Interestingly, the high chemoselectivity between the dimethylamino and diethylamino groups in 25e is attributed to the reduced reactivity of the latter due to *gauche* repulsion.

**Table 1:** Removal of the dimethylamino function on different substrates

Entry	Reagent	Product	Solvent	Yield (%)
1	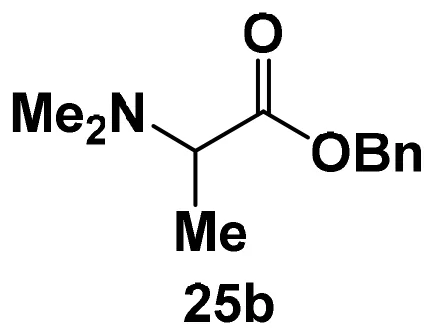	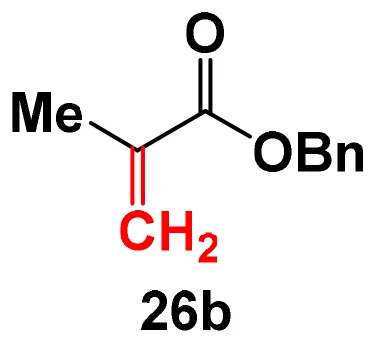	DMF	50
			2-PrOH	90
2	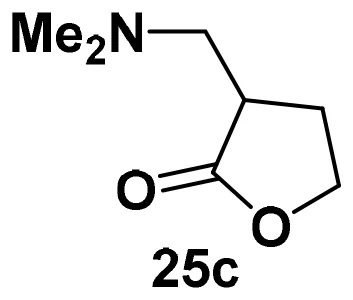	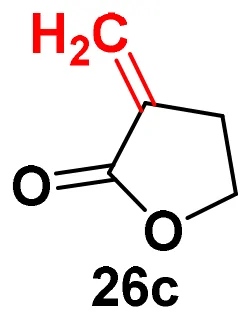	2-PrOH	77
3	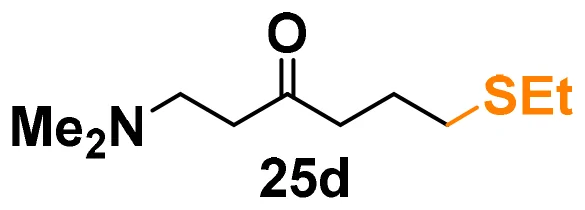	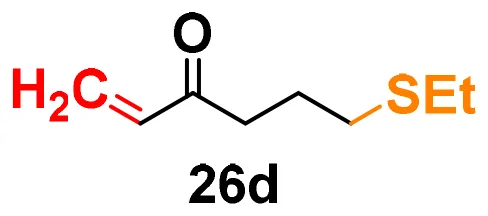	DMF	86
4	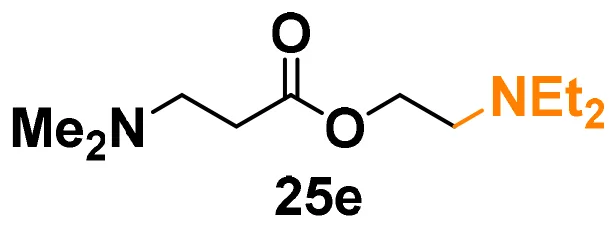	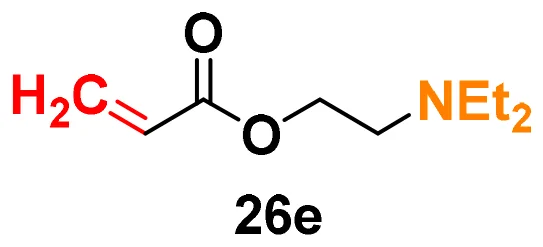	DCM	93

## 4. Reactions with different substrates

Dimethyl(methylene)ammonium salts have been widely employed in condensation reactions with active methylene compounds and arenes in a variant of the Mannich reaction.^71^ Theoretically, the use of preformed electrophiles allows overcoming, at least in principle, the limitations of the classical methods. These limitations comprise the drastic reaction conditions and the long reaction times that correlate with the presence of unwanted side reactions. In the case of unsymmetrical ketones, the regioselectivity cannot be controlled to any significant extent and is often strongly dependent on the reaction conditions; only aldehydes and ketones can normally be used, and some other carbonyl compounds such as carboxylic acids and their derivatives cannot be aminomethylated.

In the classical Mannich reaction, the iminium salt that reacts with the active substrate is reversibly generated; hence, the use of preformed reagents, such as Eschenmoser's salt, guarantees a higher concentration of the electrophile, therefore leading to lower reaction temperatures and much shorter reaction times. Consequently, many undesired side reactions are avoided even with sensitive substrates.^22^

Preformed iminium salts are soluble enough in many aprotic solvents, so the use of highly reactive nucleophiles (which ordinarily would decompose under the protic conditions of the classical Mannich reaction) is allowed.^20^

**Scheme 13:**
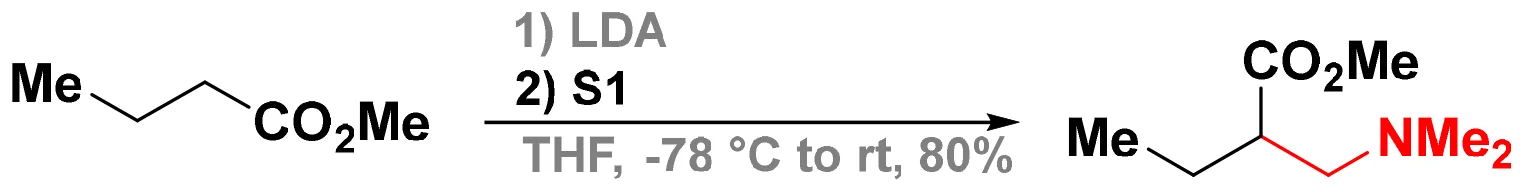
Typical reaction of Eschenmoser's salt.

Preformed kinetic enolates undergo methylation with these salts, extending the scope of the Mannich reaction to weakly acidic active methylene components such as esters (Scheme 13), lactones,^72^ unsaturated lactones,^73^ carboxylic acids,^74^ and nitriles.^75,76^

Furthermore, several advances are reported in the regioselective and stereoselective variants by using some particular reagents.^21,71^ Thus, for unsymmetrical ketones, predictable regiocontrol can be achieved if the enolate is regiospecifically prepared. Actually, enolates are generally prepared by deprotonation with strong bases such as lithium diisopropylamide (LDA)^13,76^ or potassium hydride (KH),^20,74^ or by other procedures such as cleavage of silyl enol ethers^74^ or enol carbonates^66^ with methyllithium, or alternatively by decomposition of α-diazo ketones in the presence of trialkylboranes to generate the corresponding boron enolates.^77^ Specifically, trimethylsilyl enol ethers, derived from ketones and aldehydes, can react with iminium salts in a complementary fashion to enolates.^72,74^ Thus, the reaction proceeds *via* a silyloxonium ion which hydrolyzes on aqueous workup; this strategy has been used to access *exo*-Mannich bases which do not undergo amino methylation under classical Mannich conditions. This method has been used for the synthesis of the Mannich base of (+)-camphor,^78^ proving how Eschenmoser's salt engaged extensively also in the total synthesis of natural compounds.

### 4.1. Addition to aldehydes and ketones

Dimethyl(methylene)ammonium salts are extremely well suited for the aminomethylation of carbonyl compounds. In this way, the synthesis of β-(dimethylamino) substituted carbonylic compounds can be achieved by direct reaction of the iminium salt, like Eschenmoser's salt, with aldehydes or ketones in acetonitrile or CH_2_Cl_2_.^18,79^

Ketones and aldehydes can usually be converted under mild conditions into the corresponding Mannich bases and then subsequently modified to obtain the methylene derivatives and other compounds of interest. This route is feasible for the aminomethylation of sterically hindered carbonyls as well as for the synthesis of sensitive Mannich bases; also in the case of highly functionalized carbonyl compounds, this can be done without the formation of side products. Temperature, affecting the aminomethylation, may vary from 0 °C to reflux, depending on the acidity of the carbonyls employed.

**Scheme 14:**
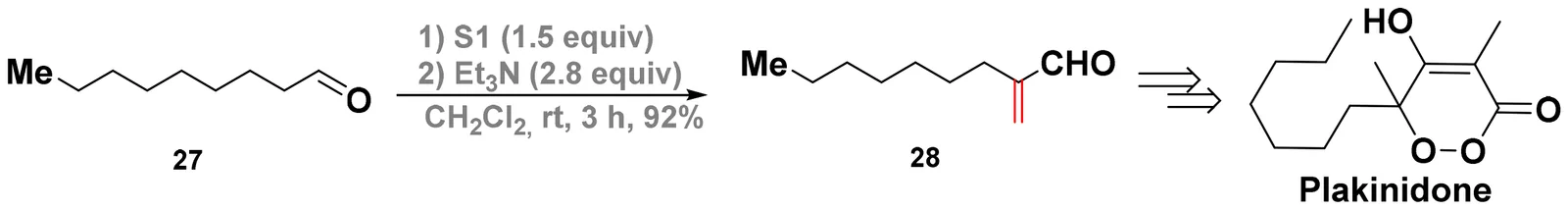
Methylenation of an aldehyde using Eschenmoser's salt S1 and trimethylamine.

Xu *et al.*^80^ took advantage of Eschenmoser's salt to easily introduce the methylene synthon at the α-carbon of 1-nonanale *en route* to a perlactone, a key synthon for the first synthesis of plakinidone, a rare cyclic organic peroxide isolated from *Plakoritis*, a marine sponge. A slight excess of the salt was slowly added to a solution of aldehyde 27 in CH_2_Cl_2_ at 0 °C, and subsequent addition of Et_3_N afforded the methylene synthon 28 in 92% yield (Scheme 14).

**Scheme 15:**
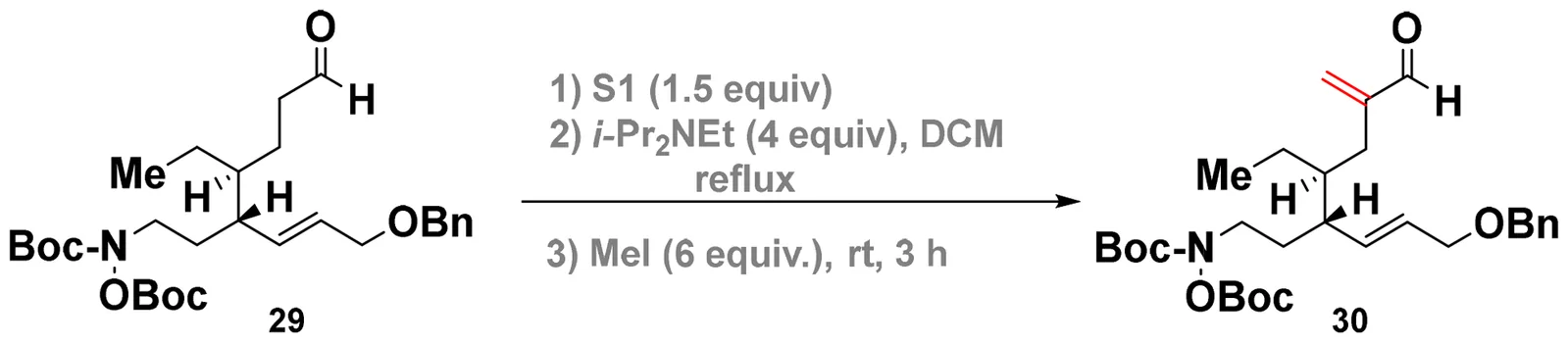
Methylenation of an aldehyde using Eschenmoser's salt S1.

In the synthesis of the marine alkaloid *Sarain A*, reported by Higo *et al.*,^81^ the insertion of the methylene group was achieved using Eschenmoser's salt S1 (Scheme 15) added to a solution of 29 in CH_2_Cl_2_ and *N*,*N*-diisopropylethylamine and then refluxed for 3 h. MeI was used to remove the dimethylamino group; after work-up and purification, enal 30 was obtained in 76% yield.

In the case of more complex substrates such as hindered and acyclic ketones, heating would benefit the outcome of the reaction increasing the rate of conversion. Conversely, for less reactive ketones the reaction time is considerably reduced in refluxing acetonitrile.^18^

**Scheme 16:**
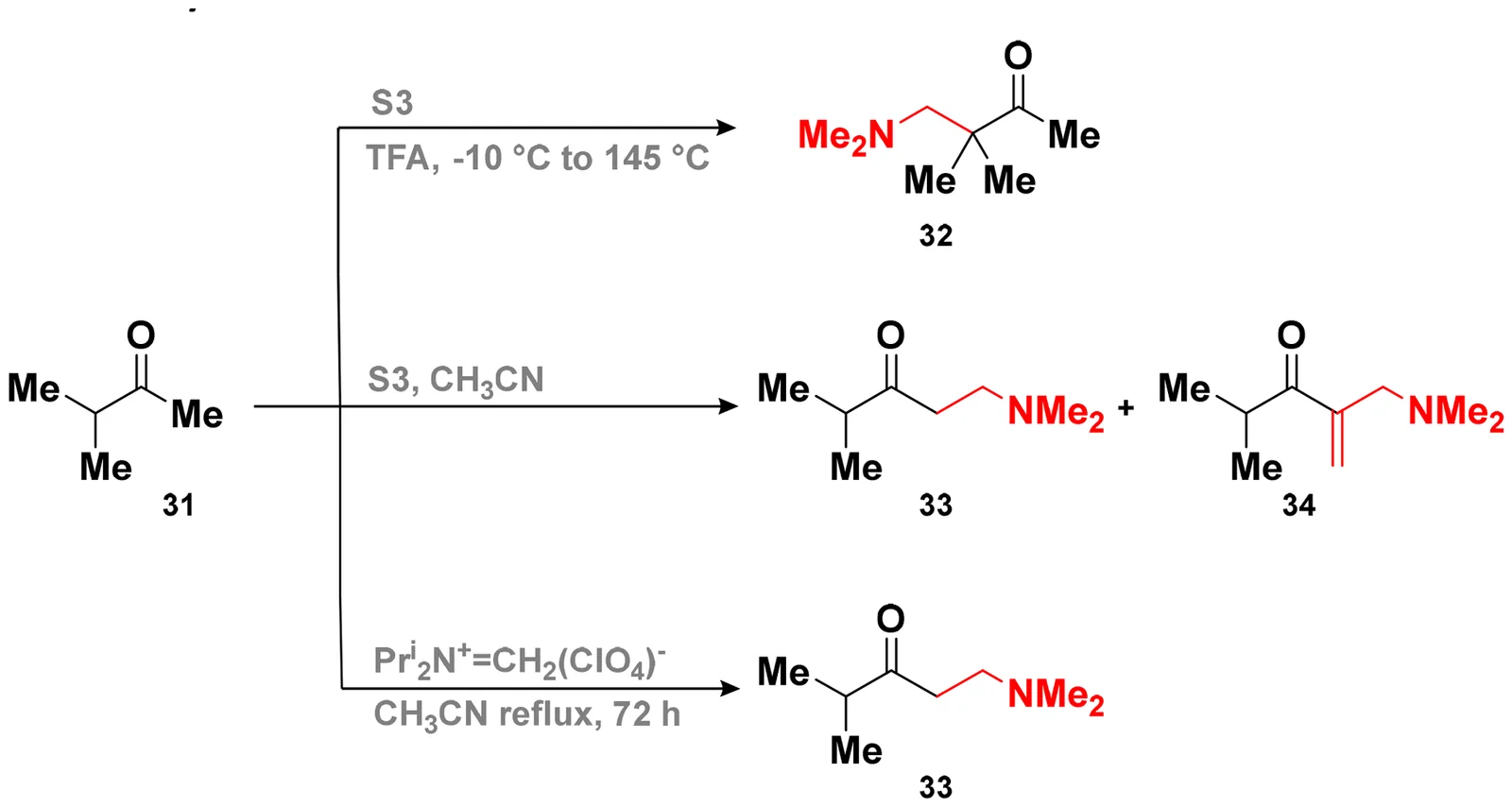
Dimethylaminomethylenation of ketone 31 using different solvents and different dimethyl(methylene)ammonium salts.

An important point to consider is regioselectivity. Indeed, the orientation of the Mannich reaction using unsymmetrical ketones in anhydrous solvents is highly dependent on the experimental conditions, solvent, and the structures of the ketone and iminium ions (Scheme 16).^31^ For instance, using trifluoroacetic acid (TFA), the reaction of an unsymmetrical ketone 31 with iminium trifluoroacetate S3 leads preferentially to highly substituted isomeric amino-ketone 32. On the other hand, the reaction occurs mainly at the less substituted α-position (3) in aprotic solvents such as acetonitrile. However, as reported by Jasor *et al.*,^30^ a significant amount of aminomethylene ketone (34) is obtained in acetonitrile due to the decomposition of the corresponding diaminoketone. This issue can be overcome employing a bulkier iminium salt, namely diisopropyl(methylene)iminium perchlorate, which affords the less substituted isomer 35 in yields up to 80%. Although this functionalization enables excellent regioselectivity, the perchlorate salt cannot be safely employed for large-scale preparations because of its potentially hazardous nature. Despite the existence of several regioselective variants, which are clearly milder and more efficient, this methodology in general provides good results even when the classical Mannich reaction fails or gives lower yields.

**Scheme 17:**
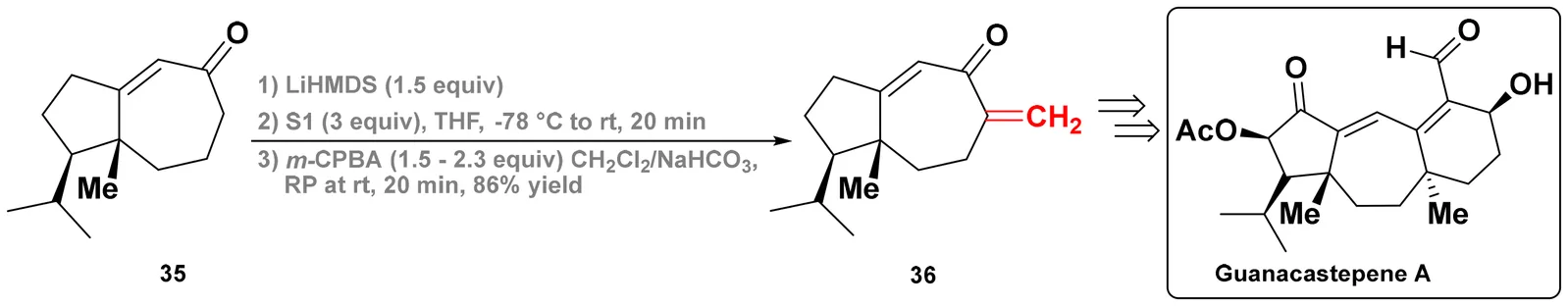
Methylenation of cyclic ketones using Eschenmoser's salt S1.

It should be noted, however, that the reaction becomes more challenging with cyclic ketones.^73^ For instance, in the case of cycloheptenone 35, the employment of Eschenmoser's salt offered an effective solution for the critical stereoselective alkylation at the C_8_ position of the key intermediate 36*en route* to *Guanacastepene A* (Scheme 17).^61^ In this transformation, a lithium base is employed to promote the reaction *via* enolization.

In general, the use of the corresponding enolates, which are more reactive toward the electrophilic salt due to their enhanced nucleophilicity, allows the reaction to proceed under milder conditions (−78 °C to rt). Moreover, employing preformed enolates allows the expansion of the scope of the reaction to less acidic methylene compounds; for unsymmetrical ketones, predictable regiocontrol can be achieved when the enolate is generated regiospecifically.^82^

### 4.2. Addition to enolates

Directly treating the carbonyl compounds with an iminium salt reagent has the obvious advantages of simplicity and feasibility in many applications. However, some limitations should be noted: (1) few compounds exhibit this type of reactivity, *exempli gratia*, for aldehydes, a competing aldol condensation is expected, which could substantially reduce the yield; (2) the extended reaction times could affect acid- or base-sensitive functionalities that might not be stable; and (3) the regioselectivity is hard to control. To resolve these problems, the employment of enolates, as nucleophilic substrates, could represent a valuable solution and a wise alternative in certain cases.

Hence, dimethyl(methylene)ammonium salts S1, S2 and S3 can quite easily react with a wide variety of enolates generated *in situ* by deprotonation of the carbonyl compound with KH,^13,74^ NaH,^51^ or LiHMDS^40–42,50,83^ (LDA in the case of carboxylic acid)^74^ or by reaction of trimethylsilyl ethers^74^ and enol carbonates^84^ with MeLi. The reaction between different types of enolates and iminium salts allows obtaining regiospecific products and aminomethylated compounds.

**Scheme 18:**
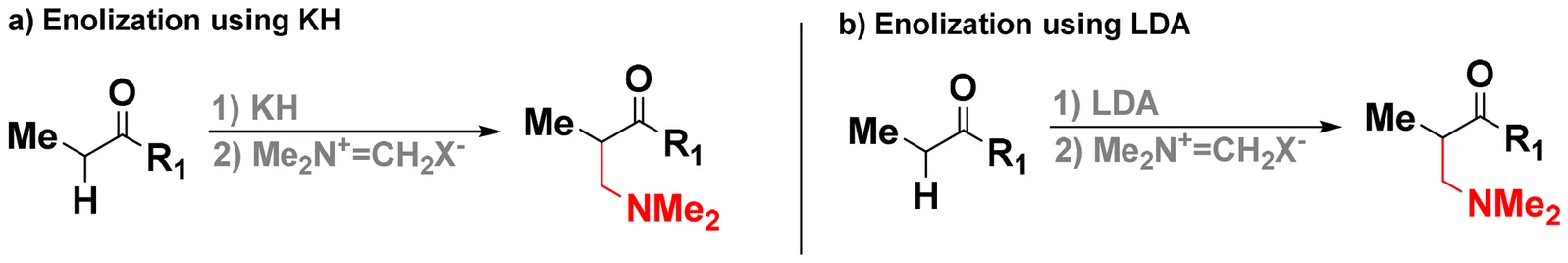
Dimethylaminomethylenation reactions using different types of reactive enolates.

The first approaches were carried out by the initial addition of a base followed by the iminium salt. The employment of lithium reagents proved to be an excellent method for yielding Mannich intermediates (of ketones, esters and lactone enolates) which, upon elimination of the ammonium salt, lead to the corresponding α-methylene compounds. In the case of the enolate generated from cyclohexanone (Scheme 18), in which equilibration of the less stable enolate with the newly formed ketoamide could decrease substantially the yield of the product, the inverse addition of the base (LDA or better KH) gave access to the desired product in a considerably higher yield.^13^

Similarly, Mandal *et al.*^61^ employed the lithium enolate of cycloheptenone 35, as shown in Scheme 17, reacting with Eschenmoser's salt, affording the dimethylaminomethylene Mannich base, which upon exposure to *m*-CPBA readily afforded the *exo*-methyleneted compound 36, a key intermediate for the enantiocontrolled synthesis of Guanacastepene A. This approach circumvents the problematic generation of an α-enolate in the original direct dialkylation strategy.

**Scheme 19:**
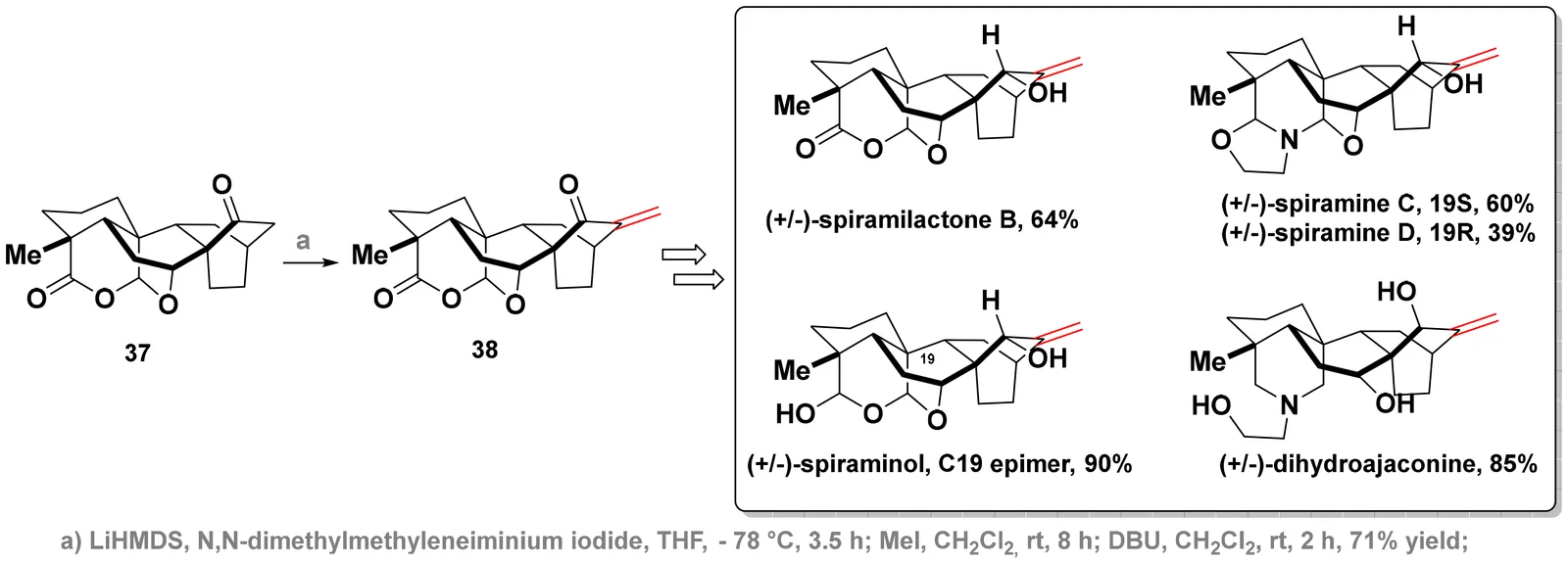
Access to atisane-type diterpenes and related diterpenoid alkaloids using Eschenmoser's salt S1.

A similar methodology was used by Cheng *et al.*^42^ in the collective total synthesis of atisane-type diterpenes and related diterpenoid alkaloids. The α-methenylation of ketone 37, *via* the reaction of the corresponding lithium enolate with Eschenmoser's salt, provided the desired exocyclic enone 38 in 71% yield. From this key intermediate, (±)-spiramilactone B, (±)-spiraminol, (±)-spiramines C and D, and (±)-dihydroajaconine were successfully accessed, leading to yields up to 90% (Scheme 19).

**Scheme 20:**
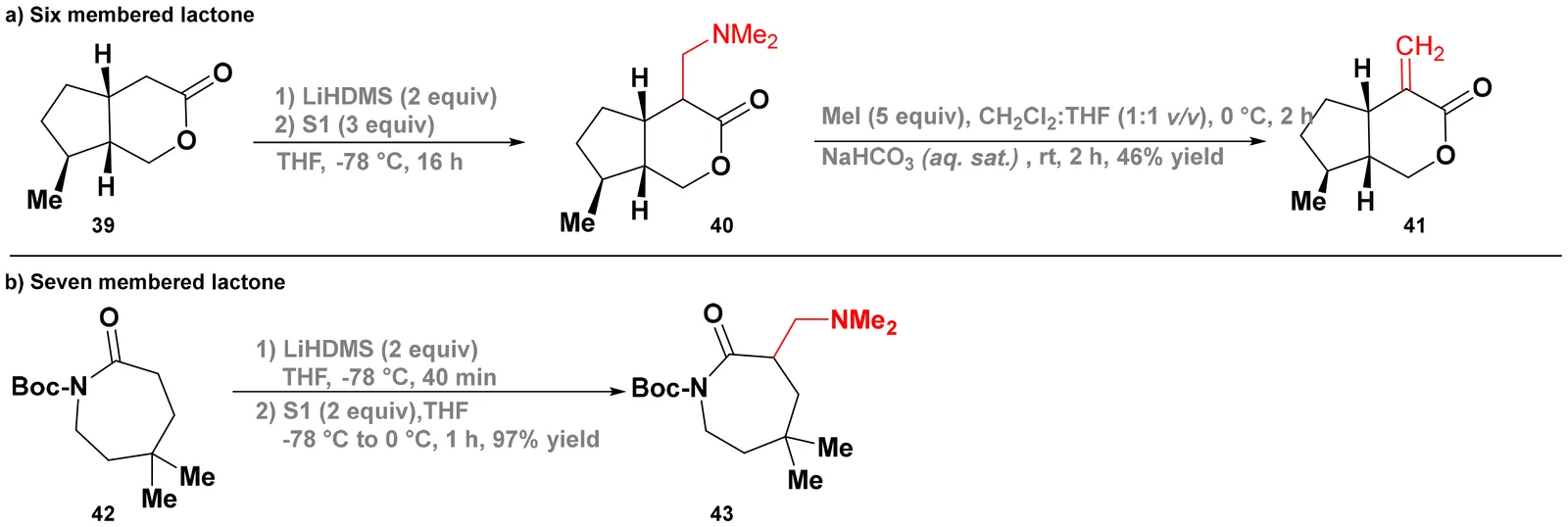
Reactions of Eschenmoser's salt S1 with two different cyclic enolates obtained by using LiHDMS.^41,85^

Also lithium lactones^41^ and lactam^85^ enolates, such as those derived from compounds 39 and 42, react with Eschenmoser's salt to deliver the corresponding β-(dimethylamino)-substituted products 40 and 43, which may undergo further elimination, leading to α,β-unsaturated compounds such as 41 (Scheme 20).

**Scheme 21:**
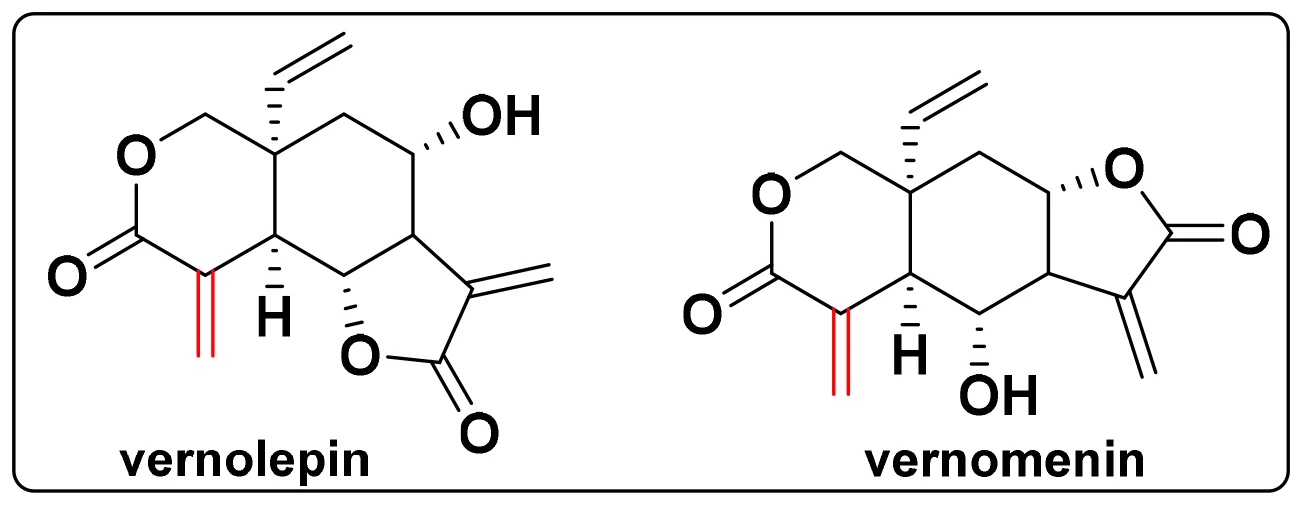
Synthesis of sesquiterpene lactones *via* the employment of Eschenmoser's salt.

Danishefsky *et al.*^72^ reported how the dimethylaminomethylation of lactones *via* their enolates, obtained by treatment with LDA in dry THF at −78 °C, illustrates the practicality of this procedure as a route to sesquiterpenes, such as vernolepin and vernomenin, without any hydroxyl group protection and reporting the first definitive structure of the two compounds (Scheme 21).

This strategy could be exploited also in reactions involving lithium^86^ and sodium^51^ ester enolates.

**Scheme 22:**
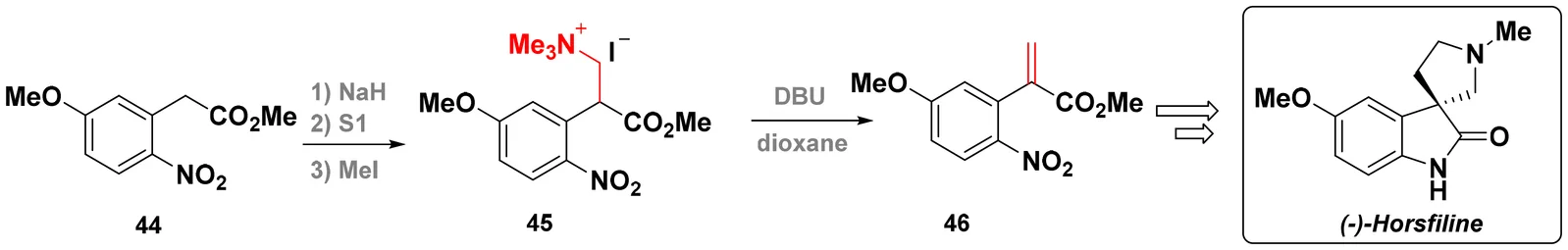
The employment of Eschenmoser's salt S1 in the highly enantioselective synthesis of (−)-horsfiline.

In this regard, Cravotto *et al.*,^51^ aiming a straightforward procedure *en route* to (−)-horsfiline and having in mind the “atom economy” of the process, reported a Mannich-type reaction to achieve the desired functionalization at the activated methylene group. Thus, as depicted in Scheme 22, the treatment of 44 with NaH led to the corresponding sodium enolate which, upon reaction with dimethyl(methylene)ammonium iodide and conversion to methiodide 45, was transformed into the desired acrylate 46 upon treatment with DBU.

Considering the excellent stereocontrol achieved with acyl-1,3-dithiane 1-oxide (DITOX) derivatives in asymmetric Mannich reactions, the corresponding methylenation reactions with Eschenmoser's salt were examined using enolates derived from *syn*- and *anti*-2-ethyl-2-propanoyl-1,3-dithiane 1-oxide.^83^ Moderate selectivity was observed with lithium-, boron-, and zinc-based enolates, whereas titanium and zirconium enolates failed to afford the desired products (Table 2 ). The poor diastereoselectivity can be rationalized by the lower reactivity of Eschenmoser's salt, which may reduce its ability to discriminate effectively between the *Re* and *Si* faces of the enolate compared to more reactive electrophiles.

**Table 2:** The employment of DITOX and Eschenmoser's salt S1 in a Mannich reaction

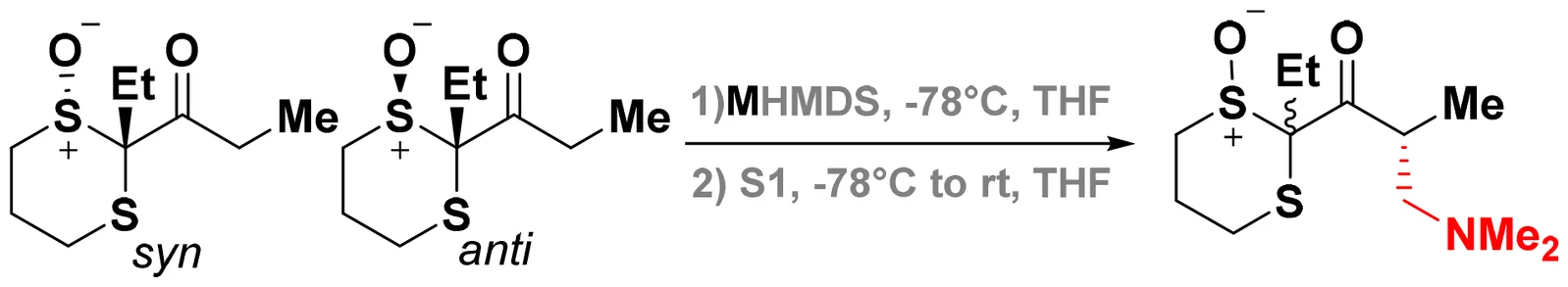			
Substrate	Metal	Selectivity	Yield (%)
*anti*	Lithium	1.6 : 1	72
*anti*	Zinc	1.6 : 1	68
*anti*	Boron	1.4 : 1	64
*syn*	Lithium	6 : 1	68
*syn*	Zinc	6 : 1	64
*syn*	Boron	4 : 1	56

Although direct treatment of enolates with an iminium reagent provides a convenient “one-pot” approach that can significantly reduce reaction times, the acidity of the resulting Mannich product may protonate the enolate, thereby lowering the yield. Moreover, when longer reaction times are employed, undesired disubstituted byproducts can be formed. In addition, the amine generated from LDA enolate formation may react with the iminium ion to produce a protonated bisaminomethylene, which can further protonate the enolate; as the concentration of the amine increases, the reaction yield correspondingly decreases.

**Scheme 23:**
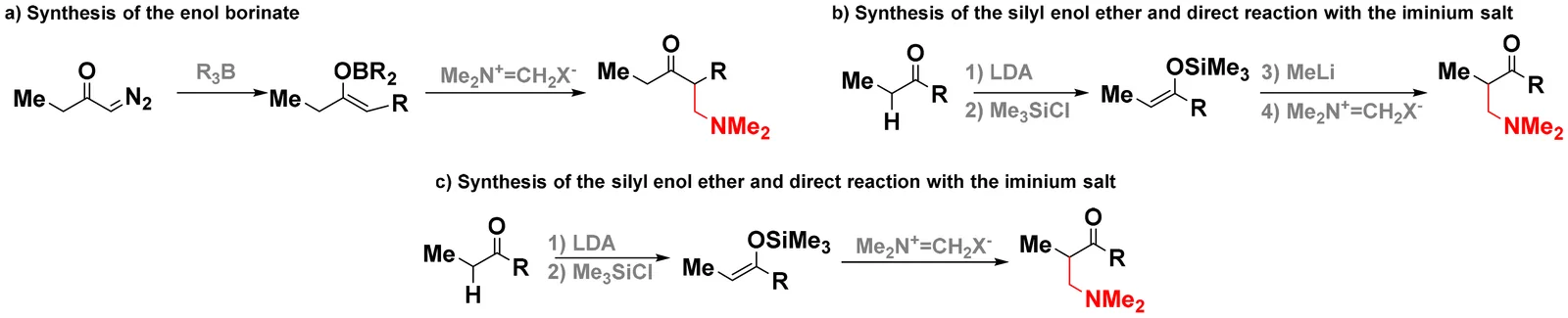
Dimethylaminomethylenation reactions using different types of reactive enolates.

To avoid the presence of an amine, a proposed solution consists of preparing either borinates^77^ (Scheme 23a) or silylated^74^ enols (Scheme 23b). Borinates must be prepared from diazoketones, therefore limiting their applicability, while silyl derivatives may be prepared in high yields from a variety of aldehydes or ketones.^87^

In general, the aminomethylation of aldehydes and ketones using dimethyl(methylene)ammonium salts is more efficient when their corresponding boron- or silyl-enol ethers are treated directly with the iminium reagent (Scheme 23c). For these processes, reaction times could vary from a few minutes to a few hours, yields are often high, and the site of attachment on unsymmetrically substituted ketones is both predictable and controllable.

### 4.3. Addition to boron and silyl derivatives

#### 4.3.1. Silyl derivatives

Silyl enol ethers are significantly better nucleophiles than the corresponding carbonyl derivatives; therefore, they could be used in Mannich-type reactions under much milder conditions, allowing the formation of Mannich bases not accessible with the classical route. So, this methodology enables effective control of stereoselectivity in the synthesis of β-aminocarbonyl compounds.

The enols obtained from the corresponding silyl derivatives may be treated with a base, namely methyl-lithium (MeLi), and subsequently with the iminium reagent at low temperature: following this strategy, the cleavage is rate-determining and the reactions are regiospecific.

**Scheme 24:**
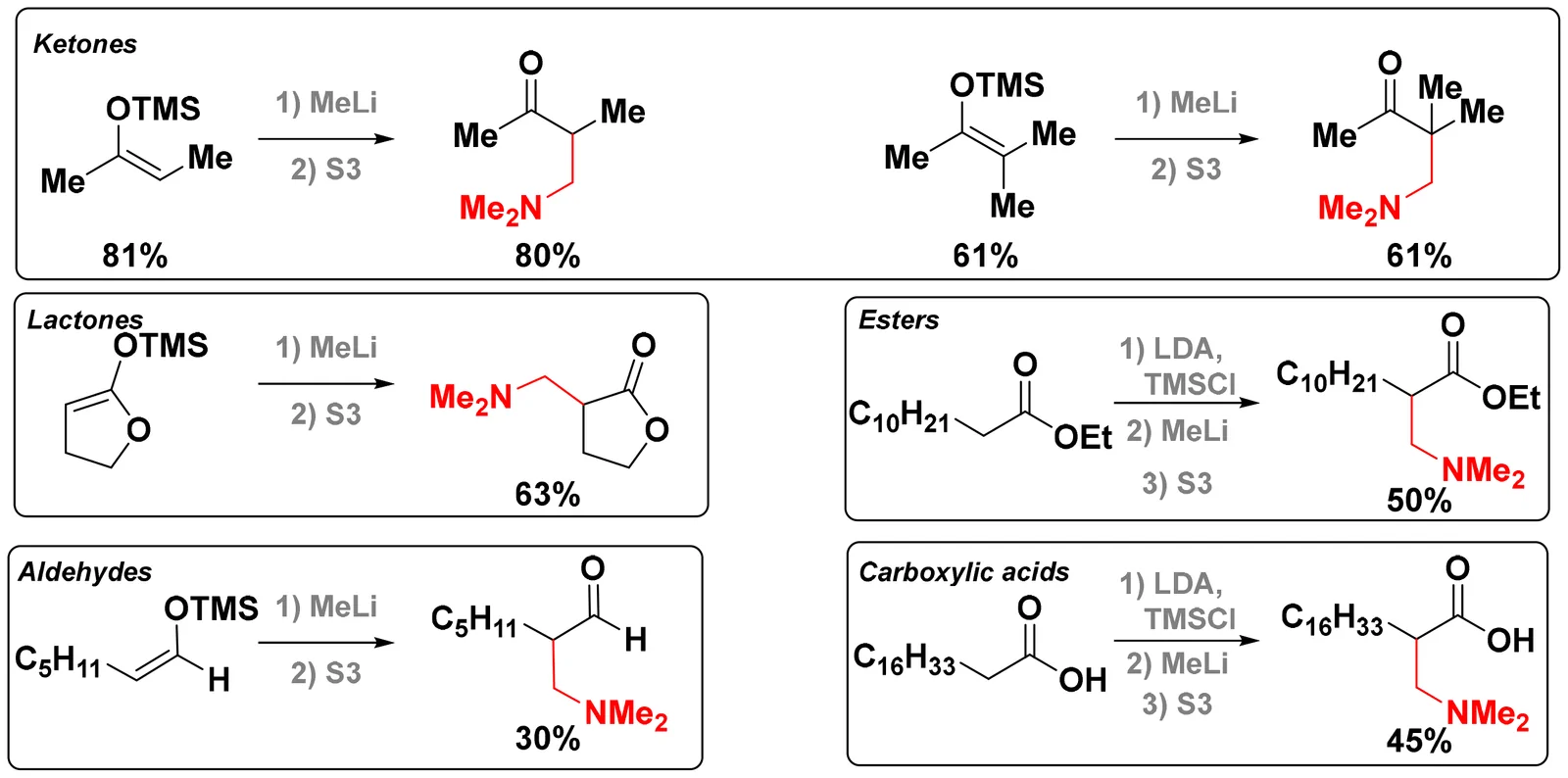
Dimethylaminomethylenation reactions reported by Holy *et al.*

As shown in Scheme 24, the silyl enol ethers of ketones and esters are generally formed in high yields; the methodology was feasible also for lactones (from γ-butyrolactone with 63% overall yield), aldehydes (from heptanal with 30% yield) and carboxylic acids (from stearic acid with 45% yield).^74^

**Scheme 25:**
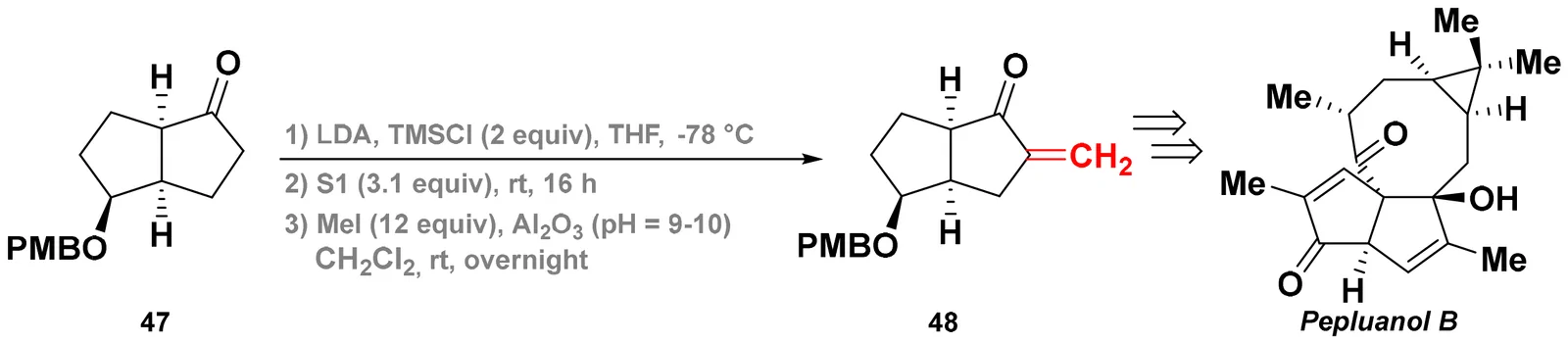
Methylenation of cyclic ketones using Eschenmoser's salt (S1).

Alternatively, and because of the deleterious effect of MeLi on several functional groups, the enol silyl ether could be combined with the iminium salt at room temperature, as reported in the case of bicyclic ketone 47 to furnish enone 48, involved in the construction of the [5–5] bicyclic system of the diterpenoid Pepluanol B in both racemic and highly enantioenriched forms (Scheme 25).^88^

**Scheme 26:**
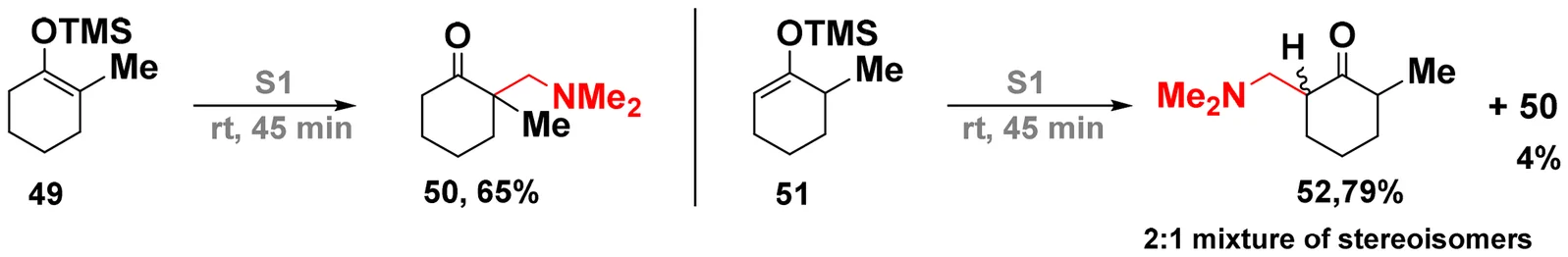
Regioselective synthesis of β-amino ketones starting from silyl enol ethers.

The aminomethylation of silyl enol ethers with dimethyl(methylene)ammonium salts is a popular method for the regioselective synthesis of β-amino ketones 50 and 52, as shown in Scheme 26. It is worth noting that, upon aminomethylation of 51, 52 is mostly formed alongside 50 (∼4%), while for the reaction of silyl enol ether 49, no detectable quantity of the regioisomeric 52 was detected.^89^

**Scheme 27:**
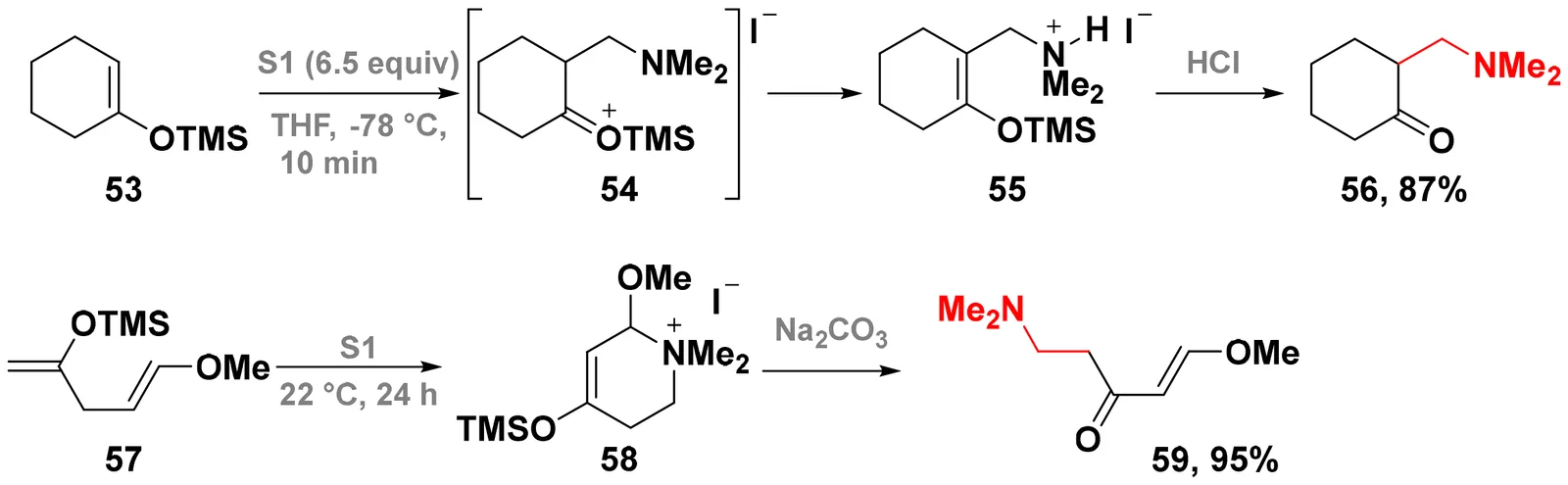
Reactions between silyl enol ethers and Eschenmoser's salt S1.

Dimethyl(methylene) ammonium iodide S1 reacts immediately with silyl enol ethers such as 53 to afford ammonium salt 55, which, upon further acid hydrolysis and neutralization, afford the β-amino ketone 56. Interestingly, cycloaddition of S1 with siloxydiene 57, followed by basic cleavage of methiodide 58, yielded the Mannich base 59 in 95% overall yield, unravelling a route to otherwise difficult to access Mannich bases of unsaturated carbonyl systems. Danishefsky *et al.*^72^ postulated that the reaction proceeded *via* the formation of transient oxonium salts such as 54 (Scheme 27).

**Scheme 28:**
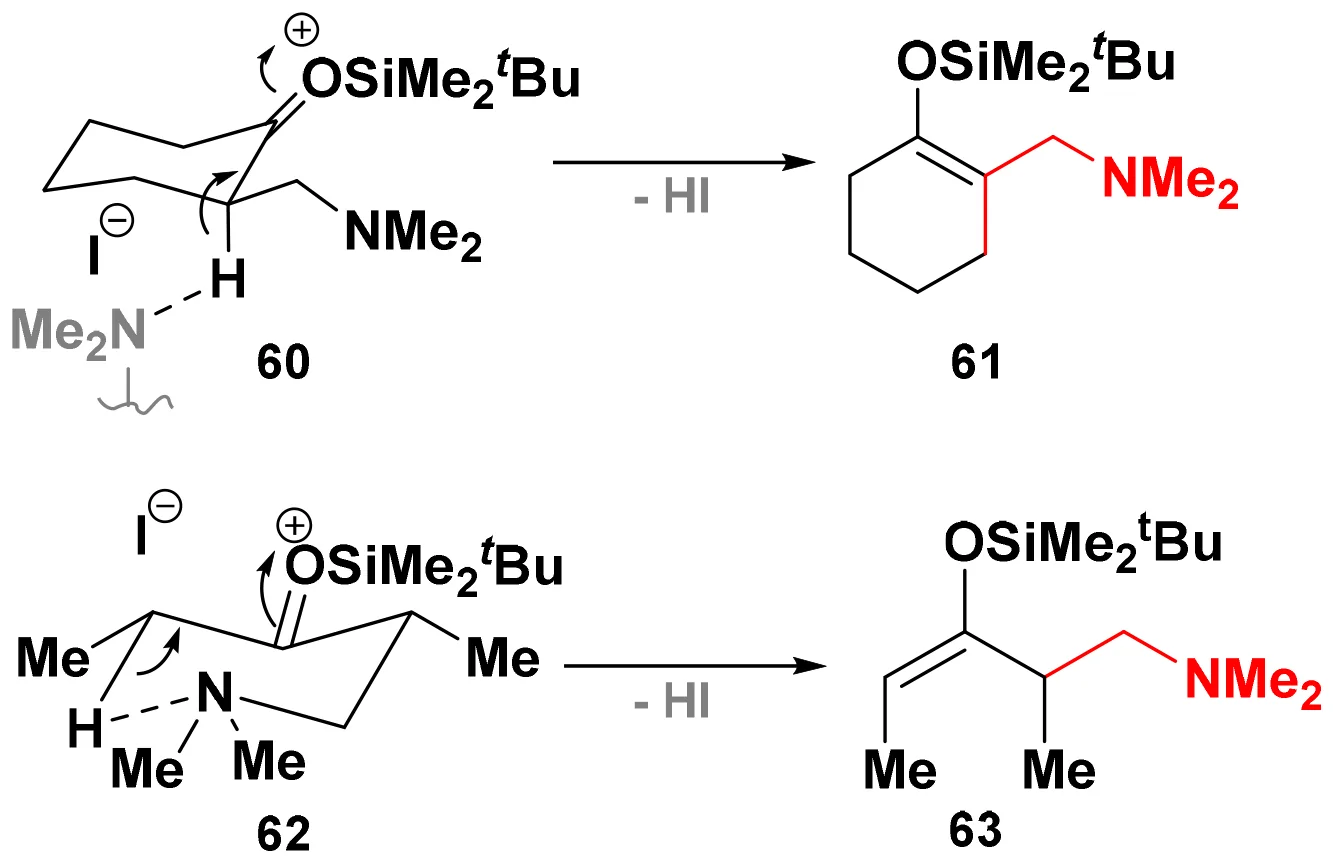
Transition states of the reaction between Eschenmoser's salt and cyclic or acyclic silyl enol ethers.

Indeed, intermediates derived from cyclic ketones can undergo regioselective α-deprotonation, while derivatives of acyclic ketones exhibit the opposite selectivity undergoing deprotonation at the α′-position. Wada *et al.*^90^ rationalized this phenomenon with the transition states reported in Scheme 28. Hence, for cyclic ketones, a transition state 60 with equatorial aminomethyl groups should be energetically more favorable. Due to steric hindrance, the deprotonation mediated by the iodide ion or nitrogen atom can only take place in an intermolecular manner, and this leads to the formation of a more highly substituted double bond, that is, a more thermodynamically favored product. On the other hand, for derivatives of acyclic ketones, a six-membered cyclic transition state 62 has been postulated, which allows an intramolecular deprotonation, resulting in a shift of the double bond and thus to the formation of products such as 63.

**Scheme 29:**
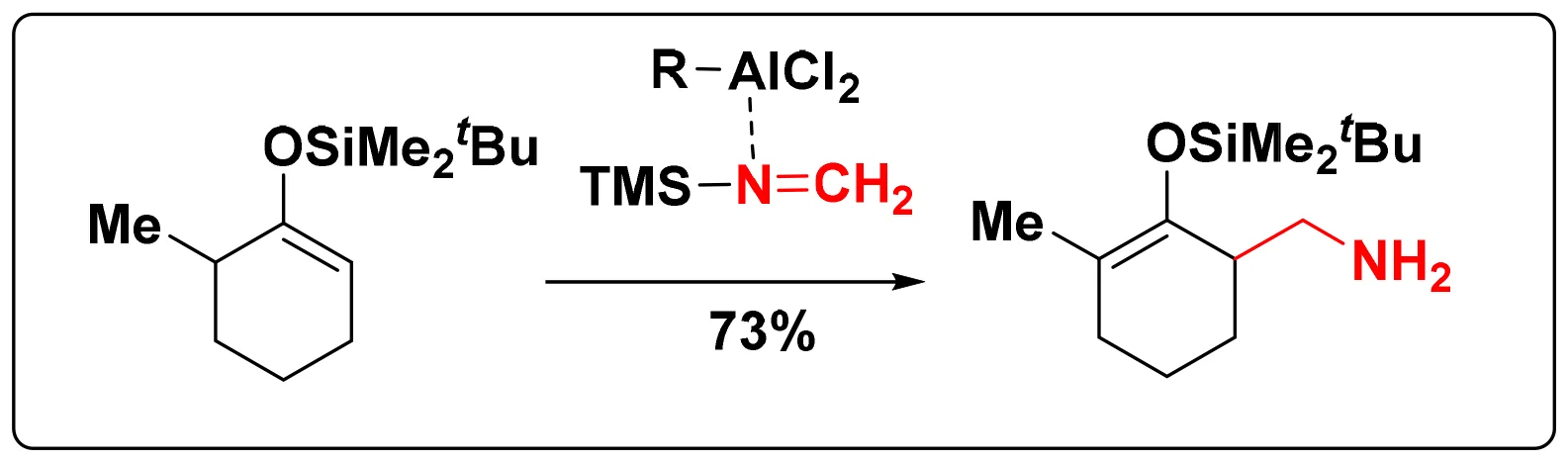
Inverse regioselectivity *via* the use of *N*-silylated formaldimines.

It is worth mentioning that the reactions carried out with *N*-silylated formaldimines, instead of S1, showed inverse regioselectivity, in which both cyclic and acyclic silyl enol ethers are deprotonated at the α′-position (Scheme 29). Thus, in this case, the presence of the Lewis acid activating the electrophile induces a more plausible stepwise reaction mechanism.^91^

**Scheme 30:**
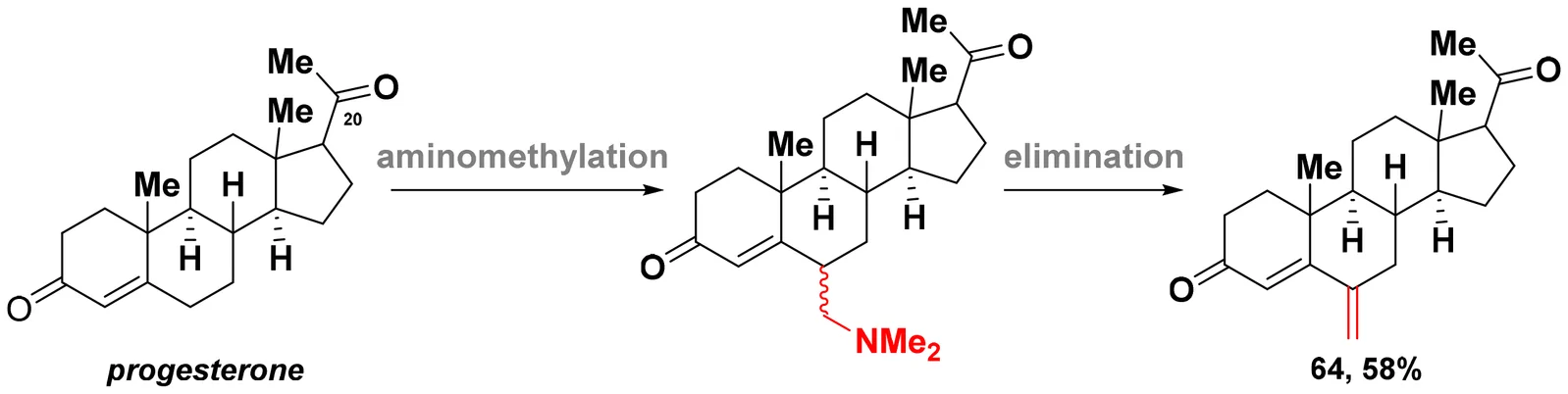
Extension of the process to the steroid series.

The use of silyldienolates demonstrated excellent feasibility for the γ-functionalization of enones (Scheme 30). This methodology was successfully extended to the steroid progesterone, which was converted to the corresponding silyldienolate and subsequently transformed into derivative 64 in 58% yield – remarkably, without the need for protection of the C_20_ ketone functionality.

**Scheme 31:**
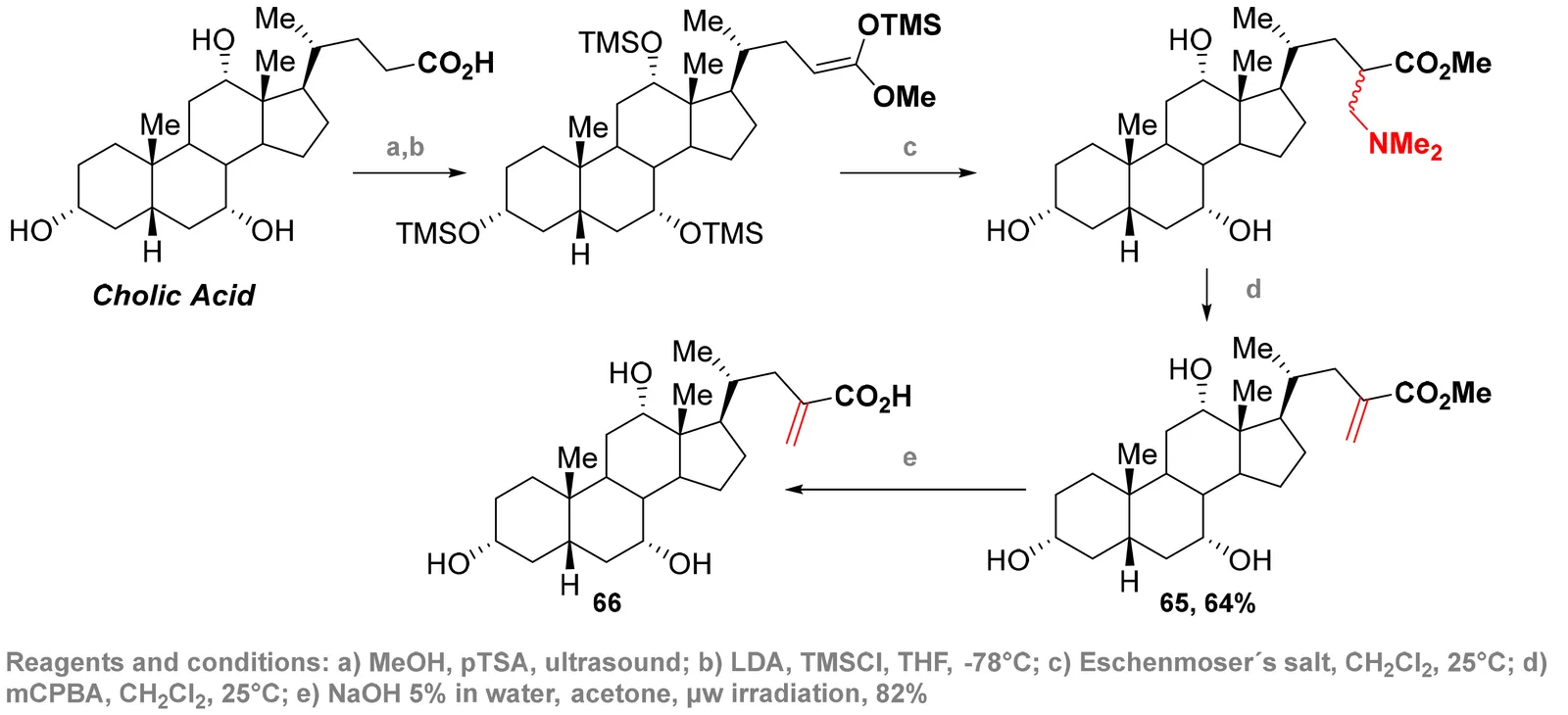
Synthesis of 23-methylen-cholic acid 66.

Recently, Rosatelli *et al.*^92^ reported the employment of Eschenmoser's salt for the preparation of 66, a C_23_-methylidene derived from cholic acid. The semisynthetic pathway enabled the isolation of 66 in 82% yield and an overall yield of 64% for intermediate compound 65 (Scheme 31). The derivatives, characterized by the presence of substituents at the C_7_- and C_23_-positions of the steroidal scaffold, have been instrumental in the design and development of selective and potent membrane Takeda G-protein receptor 5 (TGR5) agonists implicated in several physiological and pathological processes, including induction of the thermogenic program in brown and white adipose tissue, regulation of glucose homeostasis, and insulin resistance.

**Scheme 32:**
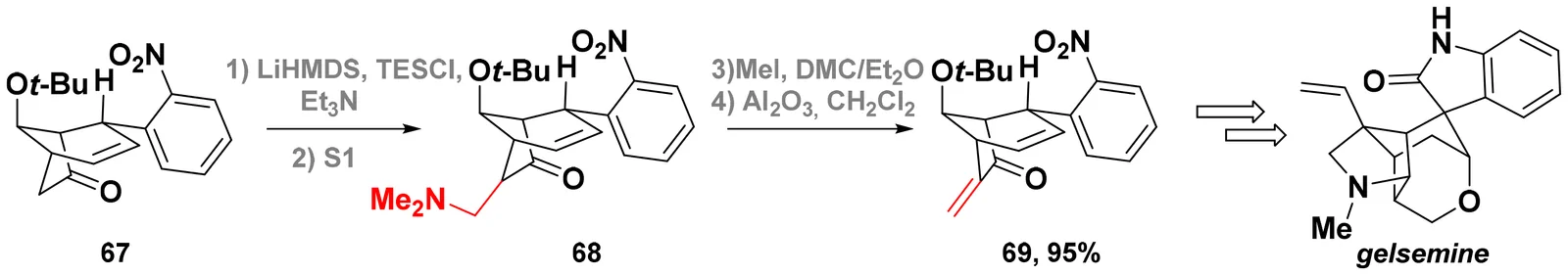
Reactions of Eschenmoser's salt for the total synthesis of *gelsemine*.

The successful employment of silyl enol ethers in reactions with Eschenmoser's salt found perfect applicability also in the total synthesis of a gelsemine alkaloid also known as *gelsemine* (Scheme 32).^93^ The oxetane precursor 67, previously converted to its silyl enol ether, was treated with S1 to afford dimethylaminomethylene ketone 68, ultimately leading to the α-methyleneketone 69 (95% yield) through quaternization of the nitrogen and α-elimination.

**Scheme 33:**
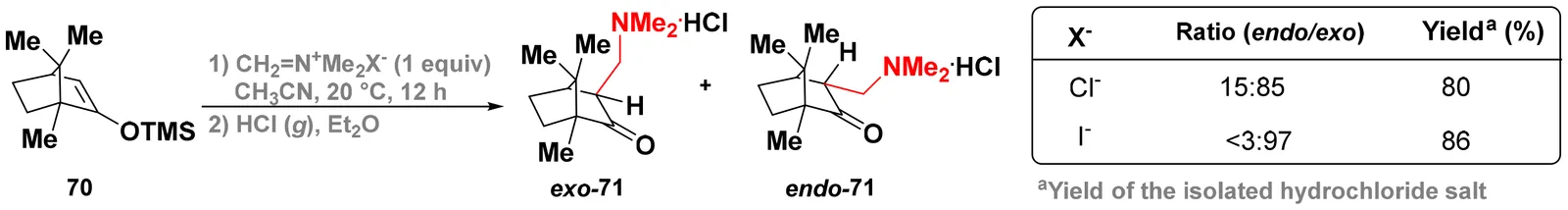
Reactions of S1 and S2 salts with the trimethylsilyl enol ether derived from (1*R*)-(+)-camphor.

In a similar fashion, the reaction of the trimethylsilyl enol ether derived from d-camphor 70 (Scheme 33) with the iminium salt predominantly afforded the *exo*-form of the corresponding α-amino ketone (*exo*-71). Both Eschenmoser's salt and the chloride analogue reacted effortlessly to produce the *exo*-isomer, although the iodide provided better selectivity, affording a crude product with less than 3% of the *endo*-71 isomer.^78^

Based on the procedure reported by Holy *et al.*,^20^ by alternatively employing potassium enolate, the experimental *exo* : *endo* ratio of the isomers was found to be 1 : 4, representing an equilibrium mixture of the two isomers, due to the basicity of the enolate anion. This means that the *endo* isomer is the thermodynamically more stable product, while the reaction with trimethylsilylenol ether, giving the *exo* isomer in excess (86% yield crude, *exo* : *endo* = 97 : 3), is guided by kinetic control.

**Scheme 34:**
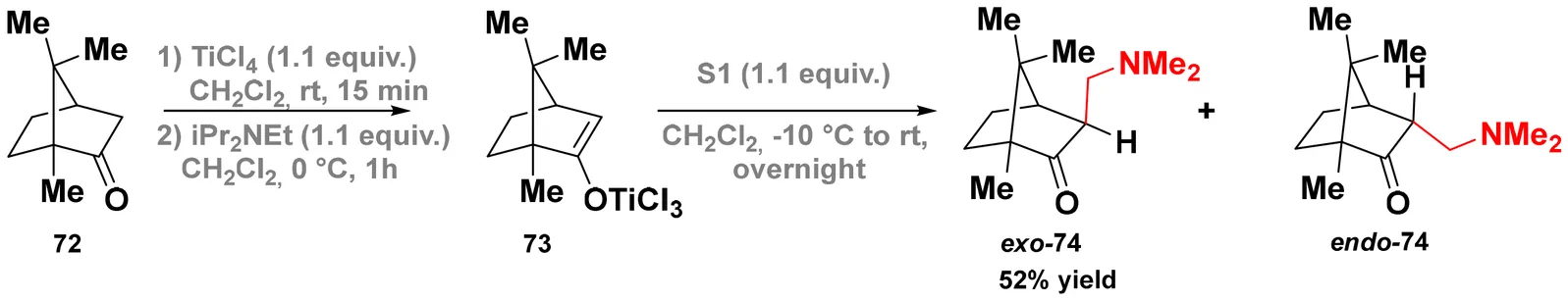
Reactions of Eschenmoser's salt with the titanium enolate of d-camphor.

Interestingly, the reaction involving Eschenmoser's salt and titanium enolate 73, obtained from d-camphor 72, afforded adduct *exo*-74 in a satisfactory yield of 52%, with an *exo* : *endo* ratio of 95 : 5 (Scheme 34), offering a one-pot alternative to the employment of the corresponding trimethylsilyl enol ether.^94^

**Scheme 35:**
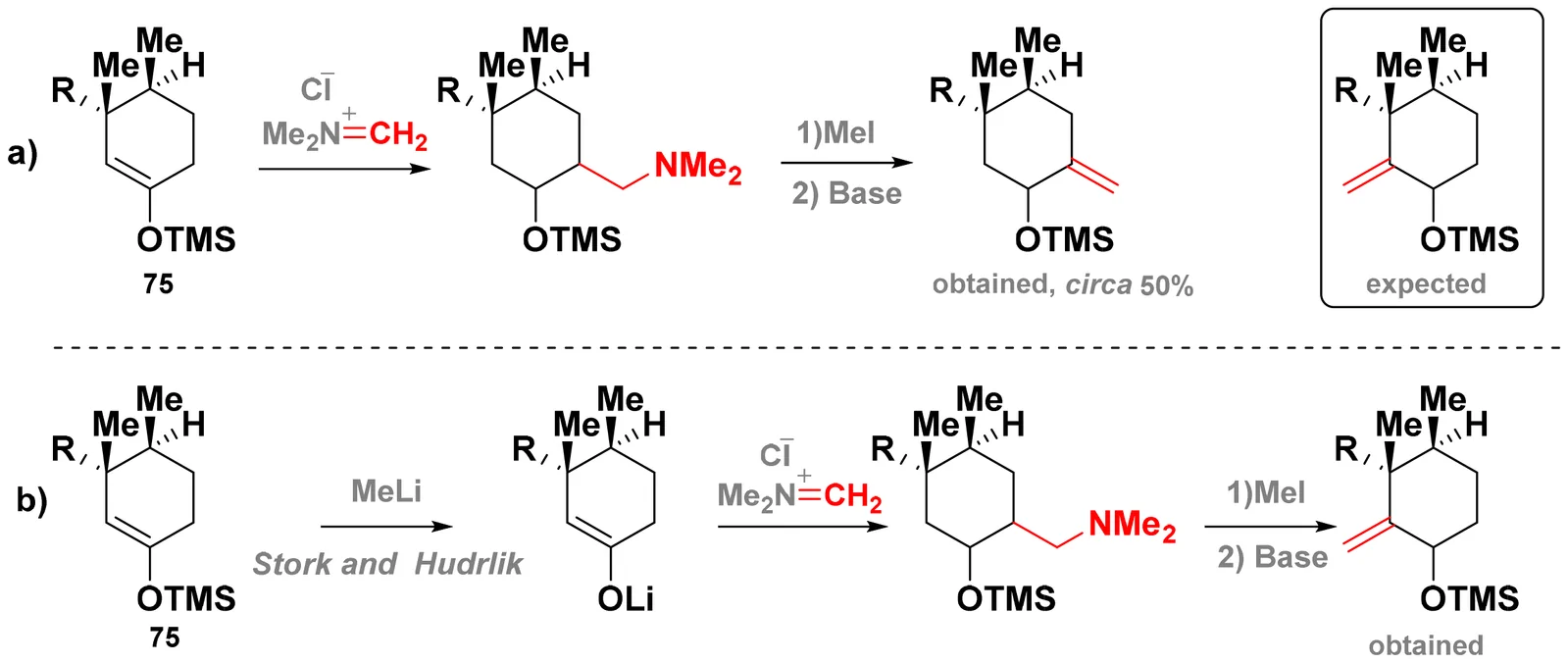
Reactions of Eschenmoser's salt with enolate.

It should be noted that deviation from the normal pathway is observed for some compounds, as in the case of compound 75, in which the reaction of the silyl enol ether with the preformed Mannich salt showed no regioselectivity (Scheme 35a).^95^

This outcome was rationalized by considering the higher steric hindrance during the attack by the Mannich cation on compound 75 (bearing a β,β,γ-trisubstitution) compared to the less substituted cyclohexenes where no specificity is lost. A solution, found in the methodology developed by Stork and Hudrlik,^96^ involves treatment of the silyl enol ether with MeLi, followed by the addition of the Mannich salt, affording the desired α-methyleneted compound (Scheme 35b). Although the exact mechanism underlying the loss of regioselectivity must be deeply studied, a reasonable explanation lies in the looseness of the oxygen–metal bond and the electron availability at the β-carbon of the enolate salt. These factors are related to the ability of the enolate to function as a base, therefore mediating the proton transfer reactions responsible for specificity loss. Consequently, lithium enolates were preferred over the sodium or potassium analogs.

#### 4.3.2. Boron derivatives

**Scheme 36:**
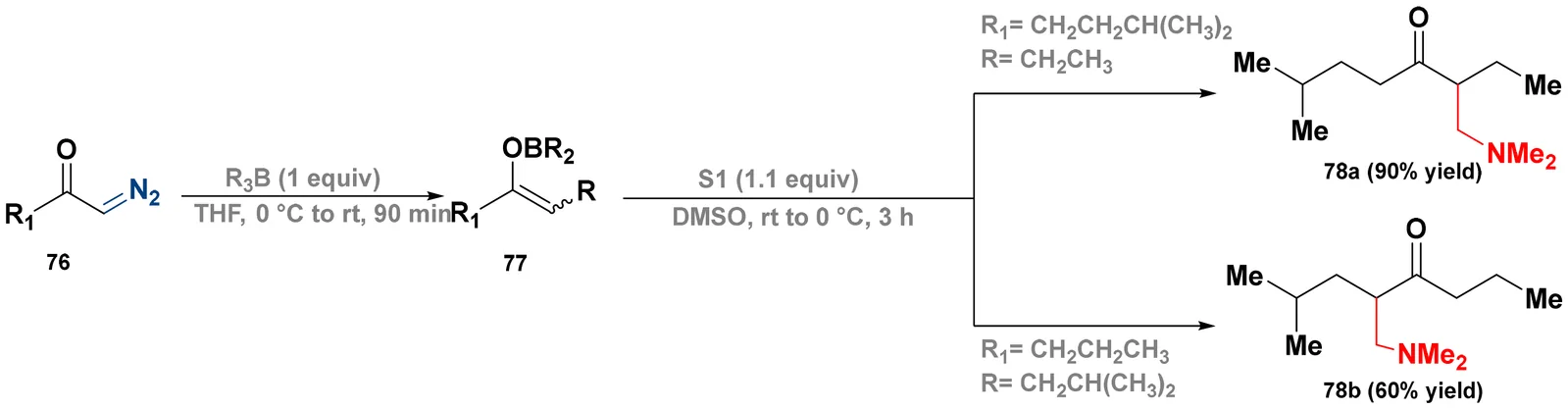
A method to selectively prepare two regioisomeric β-amino ketones tuning the starting materials.

If the side-specific α-alkylation of ketones in general requires the initial conversion of the unsymmetrical ketone to a suitable derivative, as well as the specific formation of one of two possible enol(ate)s, the reaction of boron enol ethers with Eschenmoser's salt is a powerful methodology for the regioselective preparation of β-amino ketones (Scheme 36). This method consists of the conversion of α-diazoketones 76 with trialkylboranes to give the boron enol ethers 77, which are then aminomethylated by the iminium salt. The particular advantage of this method is the highly regioselective synthesis of two regioisomeric β-amino ketones such as 78a and 78b,^77^ which are derived from ketones with almost identical α-positions by simply tuning the R and R_1_ substituents at the starting boroenol ether 77.

Even if aminomethylation of boron enol ethers allows the synthesis of regioisomerically pure Mannich bases in good yields, their use is unfortunately handicapped by the difficult experimental methodology required.

**Scheme 37:**
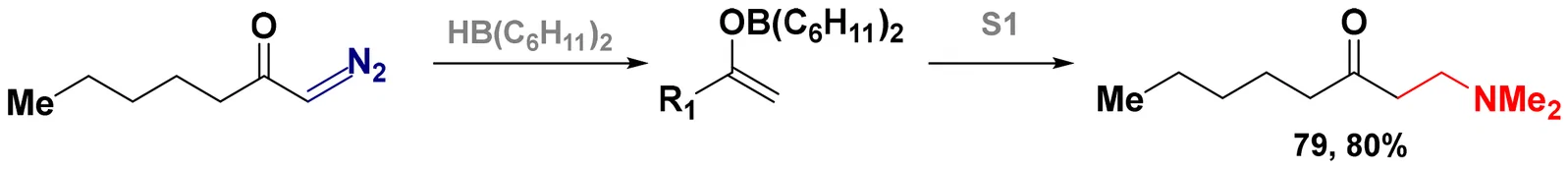
Construction of terminal enol boranes and their reaction with Eschenmoser's salt S1.

In order to develop a method for the regiospecific construction of terminal enol boranes, the employment of dicyclohexylborane was found convenient allowing the preparation of specific derivatives of methyl ketones, such as 79, upon reaction with Eschenmoser's salt^97^ (Scheme 37).

**Scheme 38:**
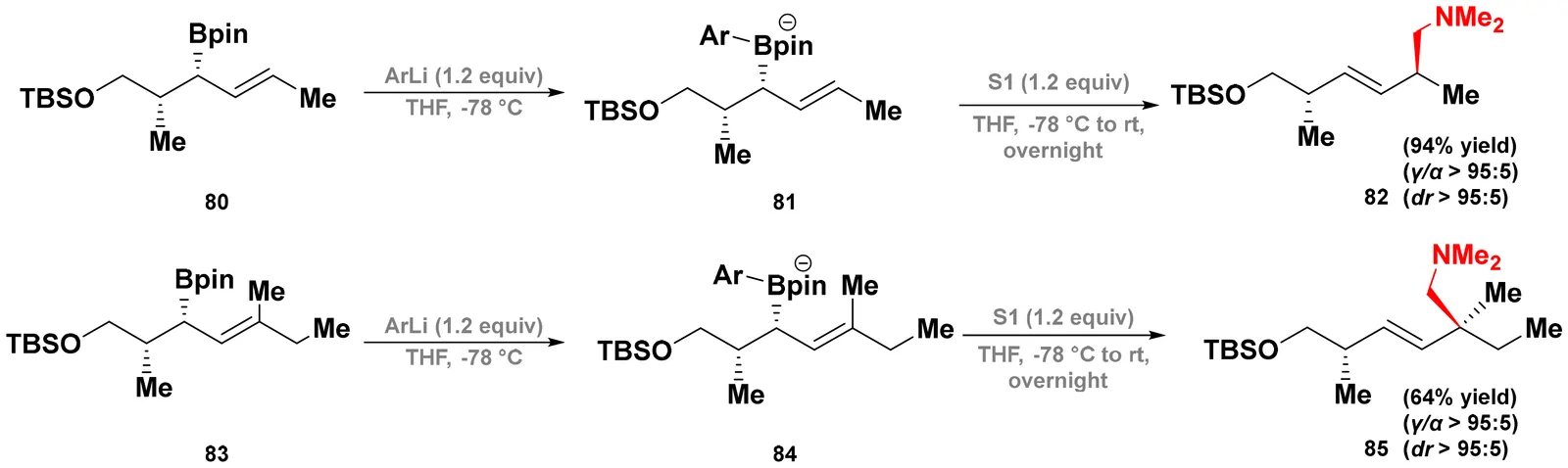
Reactions between Eschenmoser's salt and activated allylborons.^97^

Intriguingly, Eschenmoser's salt proved to be a very efficient electrophile in reactions with boronate complexes as reported by García-Ruiz *et al.*^98^ in the case of the reaction between the iminium salt and allylborons (Scheme 38). Despite the low nucleophilicity of these compounds, which had limited their broader use, the addition of an electron-deficient aryllithium (ArLi = 3,5-(CF_3_)_2_C_6_H_3_Li) activates the allylic boronic esters (80 and 83) into more nucleophilic boronate complexes (81 and 84), enabling S_E_2′ reactions with the iminium salt S1, leading to stereo-defined aminomethylated products (82 and 85). These C–C bond forming reactions show high regio- and stereospecificity and complete *E*-selectivity.

**Scheme 39:**
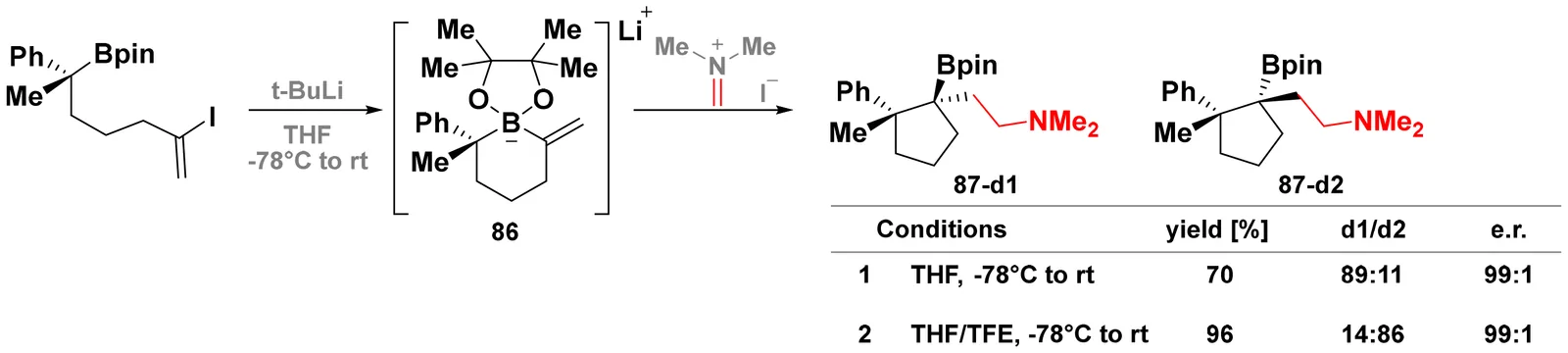
Reactions between Eschenmoser's salt and the 6-membered cyclic alkenyl boronate complex.

Based on the fact that adding an aryllithium to form a boronate complex increases the nucleophilicity of the stable boronic ester by 7 to 10 orders of magnitude. Fairchild *et al.*^99^ showed how the addition of *tert*-butyllithium to a boronic ester enabled the formation of a 6-membered cyclic alkenyl boronate complex 86, which readily reacts with Eschenmoser's salt to undergo enantiospecific and highly diastereoselective ring contraction, furnishing boronic esters 87 with high yields and 100% enantioselectivity. Interestingly, by simply adding a co-solvent, a complete inversion of the diastereoselectivity of the reaction was observed. Noteworthily, in THF at low temperature, the reaction was in favor of diastereomer 87-d1 (89 : 11), whereas with the addition of fluorinated alcohols, 2,2,2-trifluoroethanol (TFE), diastereomer 87-d2 was favored over d1 in 86 : 14 ratios and with 100% enantioselectivity (Scheme 39).

**Scheme 40:**
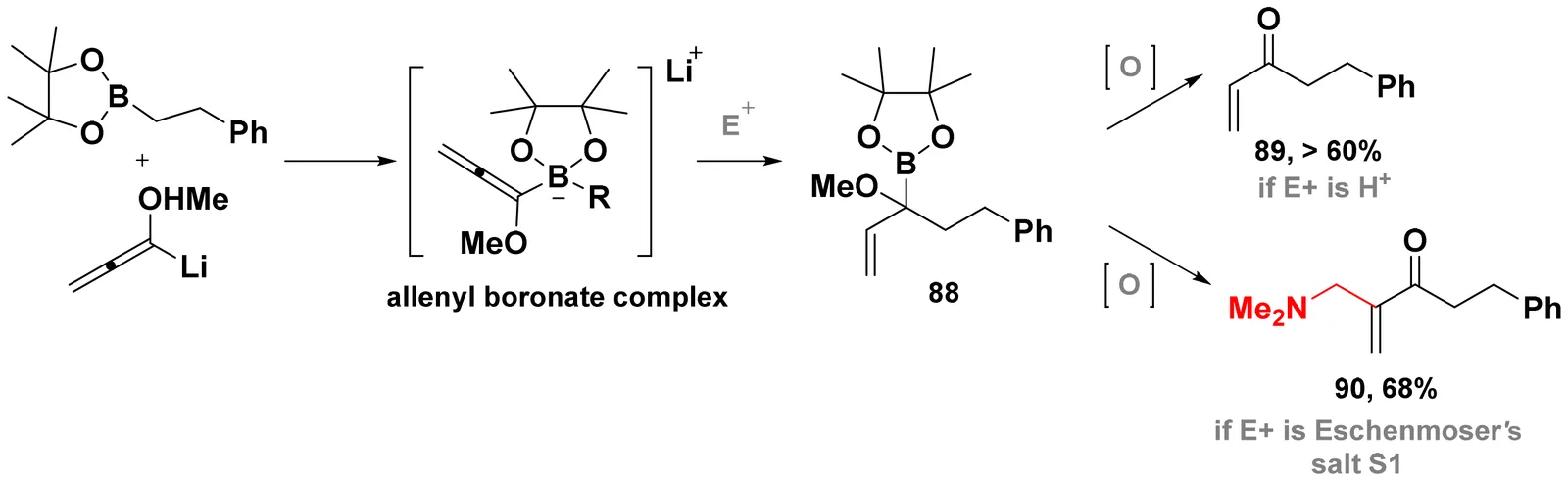
Synthesis of α-substituted enones and one-pot synthesis of α,β-unsaturated ketones from boronic esters.

In the context of C–C bond formation, the addition of a functionalized metalated element to a competent electrophilic linchpin represents a straightforward methodology for accessing a broad range of substrates. In fact, taking advantage of the unique properties of alkoxyallene or also n–π conjugated heteroallyl systems,^100^ a variety of synthetic pathways became available. For instance, taking inspiration from the work of Suzuki,^101^ describing the reaction of methoxyallenyl lithium with alkyl boranes to form active allenyl borane complexes, the employment of methoxyallene (as a three carbon building block) and boronic esters generated an allenyl boronate complex, which upon protonation could undergo 1,2-migration to give allylic boronic intermediate 88.^102^ The use of boronic esters, unable to undergo the typically rapid 1,3-borotropic shift observed with boranes, combined with the induction of 1,2-migration using electrophiles and subsequent oxidation of the resulting intermediate 88 with NaBO_3_·4H_2_O, enabled the synthesis of a broad range of densely functionalized enones (*e.g.*89, >60% yield). Eschenmoser's salt proved to be an effective electrophile in this reaction, allowing the introduction of an amine functionality to furnish enone 90 in 68% yield (Scheme 40).

### 4.4. Addition to unsaturated compounds

Unsaturated compounds such as alkenes,^103^ aromatic compounds^104^ and different types of heterocycles^105–107^ are good substrates for reacting with Eschenmoser's salt and its analogues. In this context, the dipolar nature of diazo compounds makes them susceptible to addition to different electrophilic reagents, enabling, for example, homologations or epoxidations. This reactivity, coupled with their easy preparation from readily accessible precursors, allows their use as versatile reagents in a plethora of metal carbene transformations.^108^ A great variety of experimental procedures, avoiding the hazardous employment of diazomethane, is extensively reported in the literature.^109^ Thus, the reactions of vinyl diazo compounds, in which the alkene is directly attached to the diazo functional group, with electrophilic reagents have the potential to offer complementary processes to those of metal carbenes. The employment of suitable electrophilic partners could allow the retention of the diazo functionality, providing structurally complex diazo-containing compounds.

**Scheme 41:**
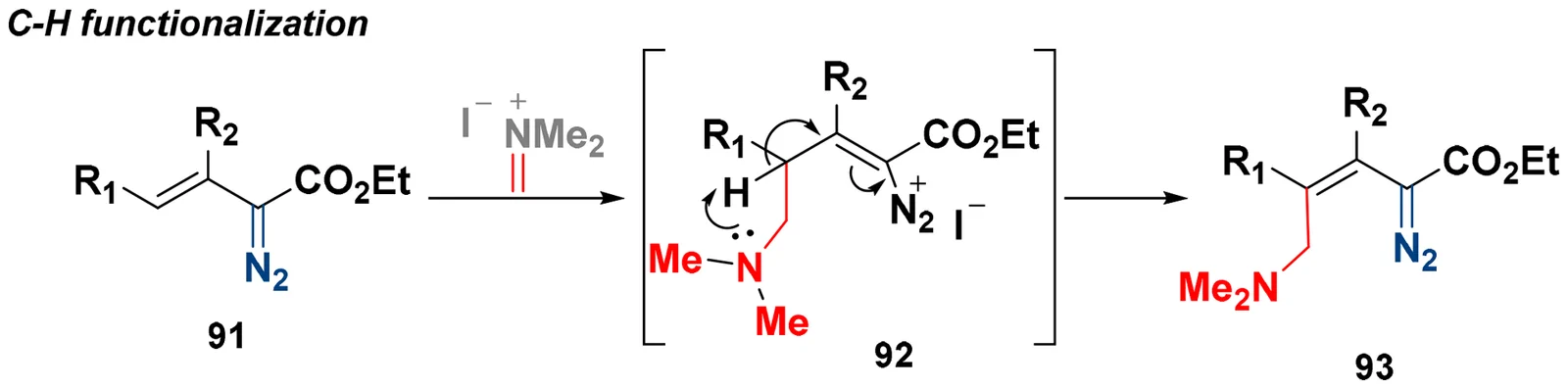
C–H functionalization proposed mechanism.

When applied to vinyl diazocompounds such as 91,^110^ Eschenmoser's salt underwent a formal C–H functionalization reaction yielding *E*-substituted vinyl products in high yields and stereoselectivity. The plausible mechanism is rationalized by the formation of a vinyl diazonium intermediate 92, which, upon deprotonation, delivers the formal C–H functionalization products 93 (Scheme 41).

**Scheme 42:**
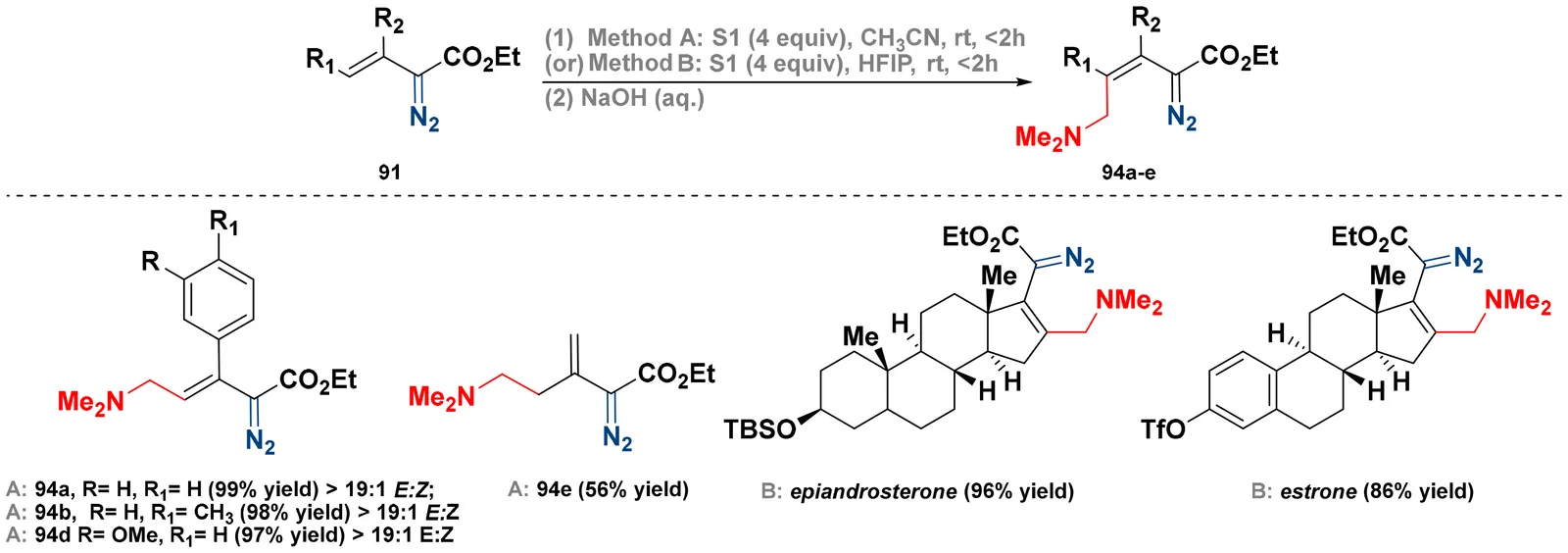
Reactions between Eschenmoser's salt S1 and vinyl diazocompounds with high stereoselectivities.

The process tolerates both electron-withdrawing and electron-donating substituents, yielding compounds 94a–d in 91%–99% yields with >19 : 1 *E* : *Z* ratios. Interestingly, product 94e was isolated in 56% yield from the β-methyl-substituted vinyl diazo compound upon an addition–elimination sequence, as depicted in Scheme 42. Furthermore, natural products *epiandrosterone* and *estrone* derivatives undergo the transformation at room temperature in 1,1,1,3,3,3-hexafluoro-2-propanol (HFIP), delivering the corresponding products in 96% yield and 86% yield, respectively.

**Scheme 43:**
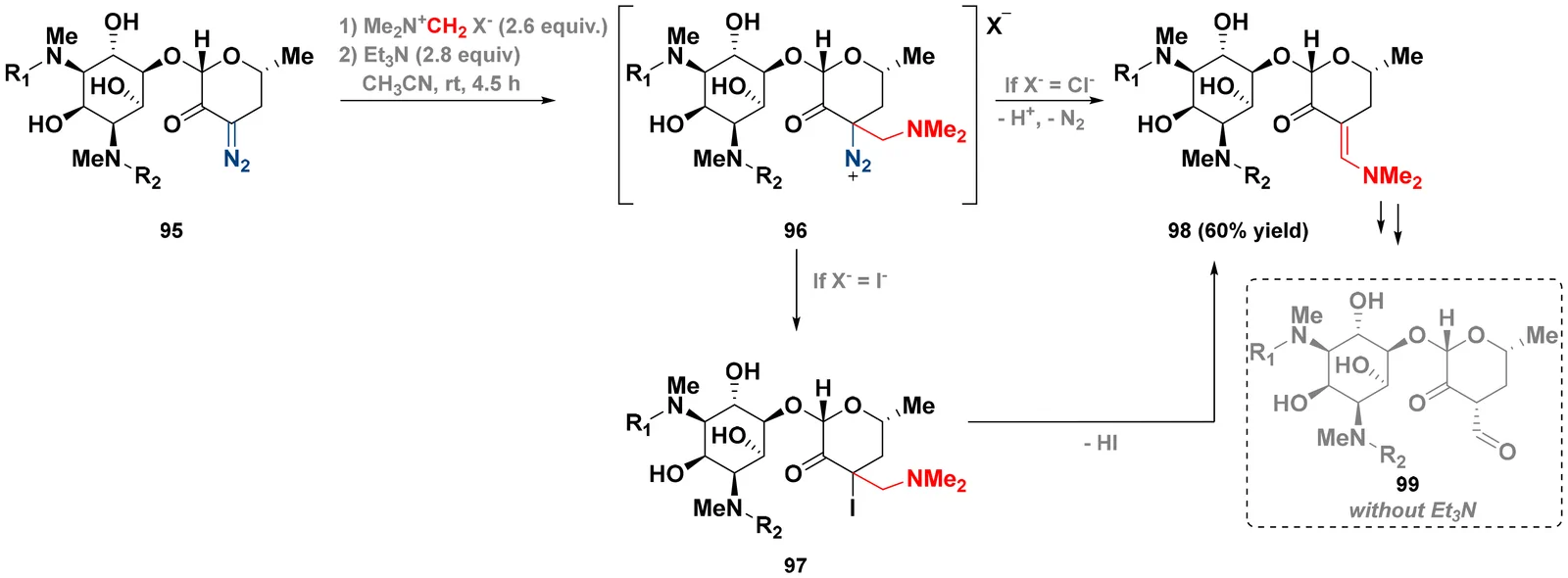
Reactions between Eschenmoser's salt S1 and an α-diazoketone.

As reported by Thomas and Fritzen,^111^ α-diazoketones 95 could react with methaniminium salts, releasing N_2_ and affording vinyl amide derivatives 98. While Eschenmoser's salt promotes a rapid reaction, by-product 97 may be formed due to the high reactivity of iodide. Employing a suspension of the chloride salt in the presence of triethylamine as an acid scavenger (thereby preventing HCl-induced hydrolysis to aldehyde 99) allowed the desired product 98 to be isolated in high yield (Scheme 43).

**Scheme 44:**
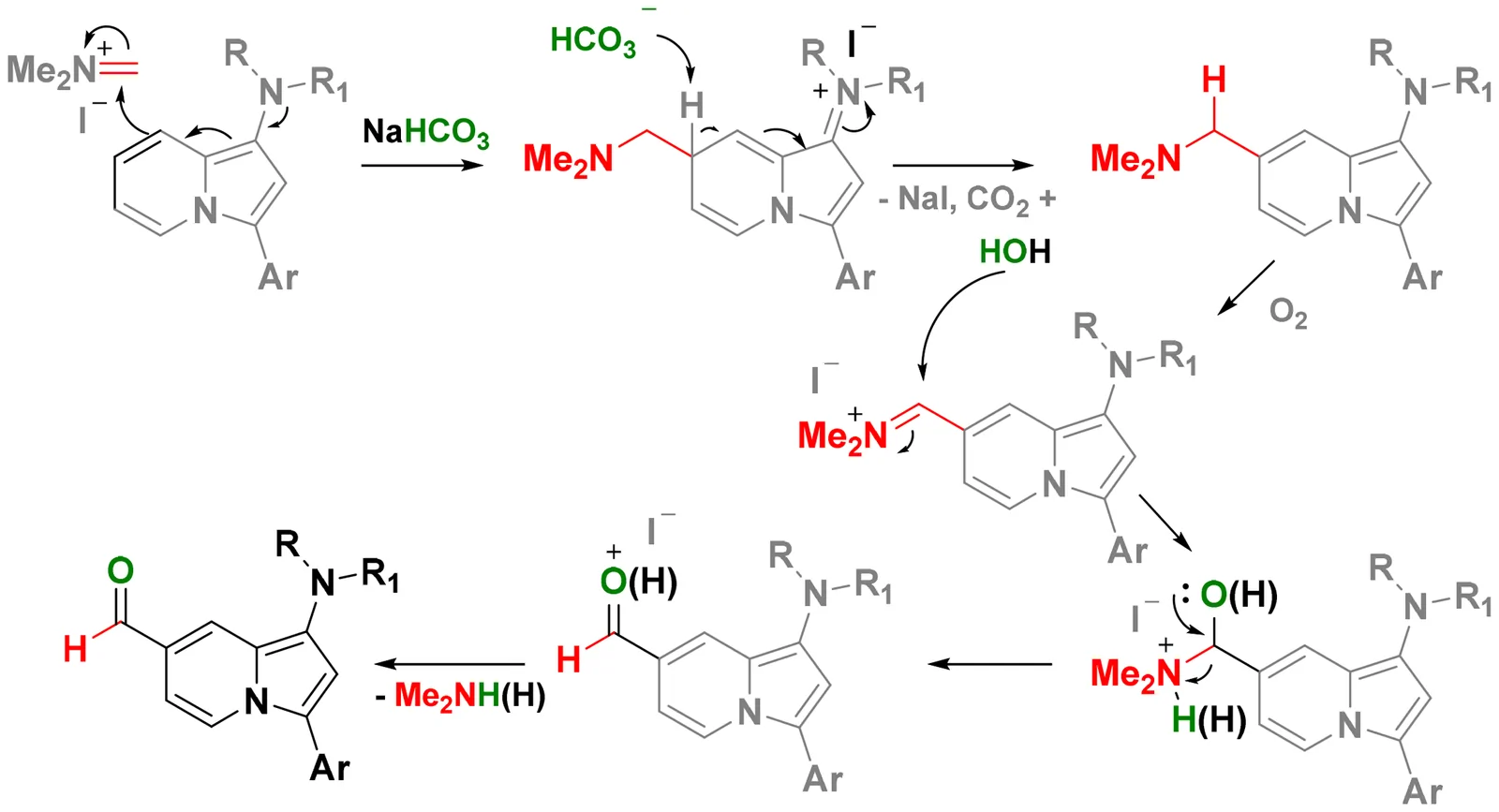
Mechanism of the C–H formylation reaction with Eschenmoser's salt.

Eschenmoser's salt employed in C–H functionalization can also be applied in the late-stage C–H formylation of heterocycles, in which it proved to be a valuable direct and regioselective formylating agent for indolizines.^112^ In accordance with the theoretical aspects, adapting the protocol to the *in situ* generation of the salt resulted in lower isolated yield and longer reaction time. The mechanistic aspects of the protocol (depicted in Scheme 44) point towards the Friedel–Crafts-type reaction between the indolizine and Eschenmoser's salt to form an iminium ion, followed by a base promoted re-aromatization to give Eschenmoser's product, which finally undergoes oxidation in air to generate a (dimethyl)iminium ion. A subsequent addition of water leads to a hemiaminal, which undergoes simultaneous liberation of dimethylamine and the formation of the C=O bond.

**Scheme 45:**
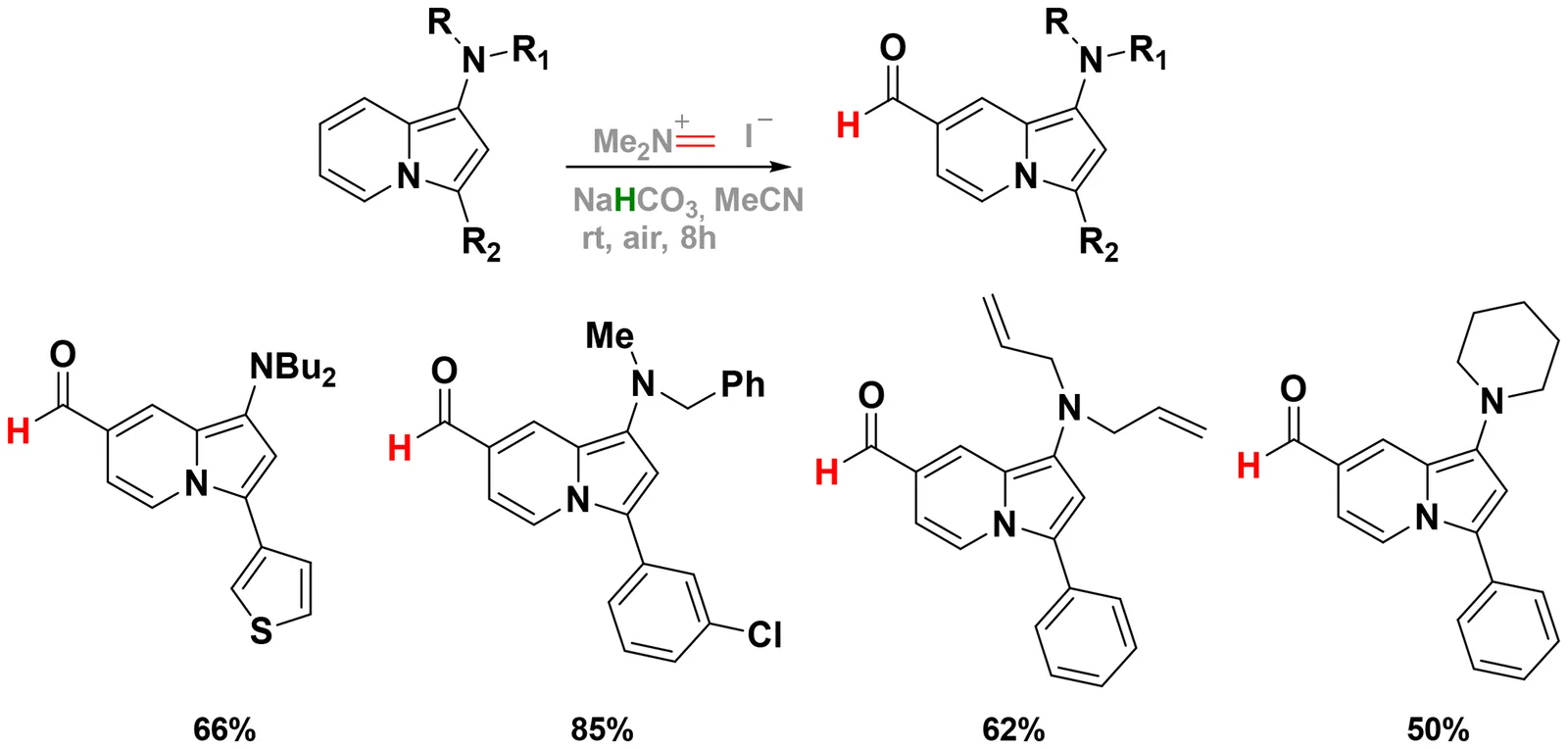
Scope of the C–H formylation reaction with Eschenmoser's salt.

The optimized formylation conditions were extended to indolizines derived from secondary amines, giving the corresponding products in yields ranging from 30 to 85% (Scheme 45).

**Scheme 46:**
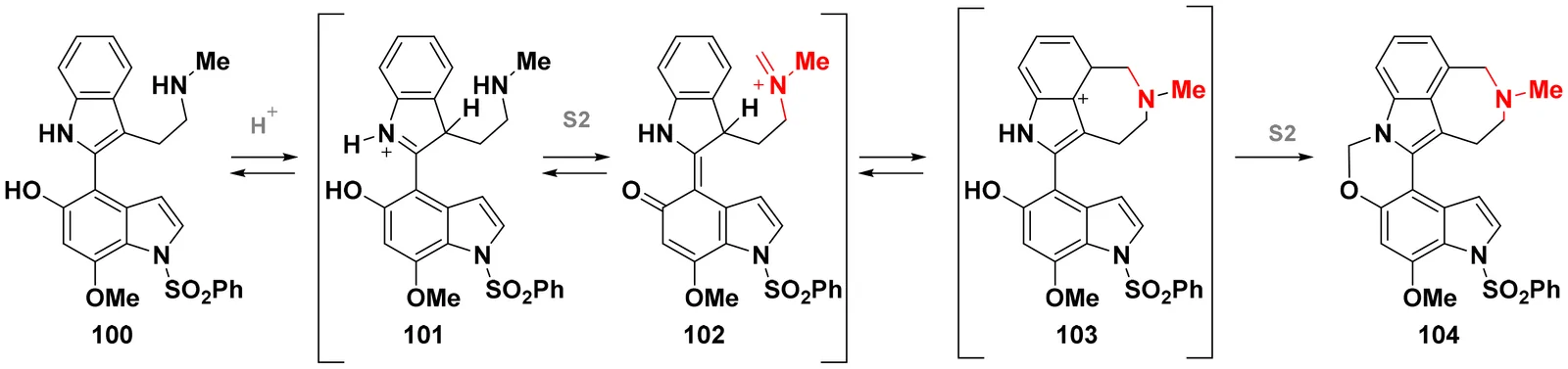
Reaction of the chloride iminium salt with an amine.

Similarly, salt S2 has been employed for the cyclization of tryptamine derived bisindole substrates such as 100 (Scheme 46)^113^*via* intermediate 102, which undergoes Friedel–Crafts alkylation to give the desired product 104.

On the other hand, indoles are considered some of the most valuable compounds in pharmaceutical research, as they are versatile pharmacophores present in many natural products, drugs, agrochemicals and other biomolecules. The development of indole chemistry and, in particular, functionalization strategies is experiencing a rapid development and represents a major advancement in many fields.^114^

**Scheme 47:**
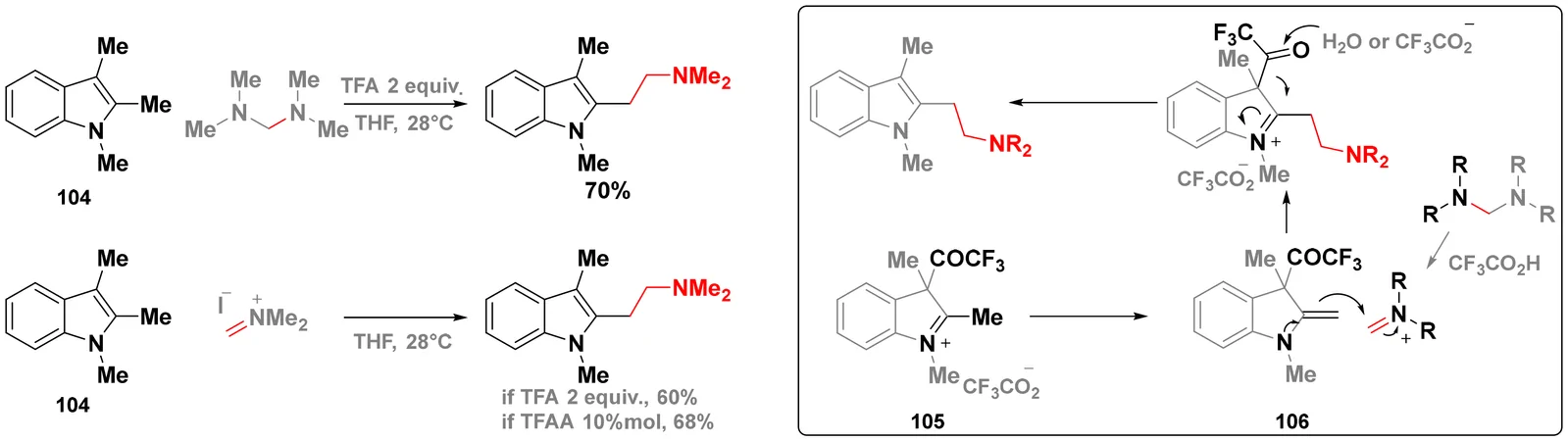
Umpolung strategy for the C(sp^3^) H aminomethylation of 2-methylindole.

In the context of metal-free induced C(sp^3^) H functionalization of indoles *via* nucleophilic addition to electrophiles, an elegant acid-mediated umpolung of 2-methylindole was reported by Kong *et al.*^115^ to accomplish C(sp^3^) H aminomethylation using aminals as electrophilic partners and employing TFA as a catalyst (Scheme 47). The use of TFA was crucial in the process for promoting the formation of aminomethylated products. To prove and understand the indispensable roles of TFA in the process, Eschenmoser's salt was used in the reaction with 1,2,3-trimethylindole 104. Surprisingly, the reaction did not proceed in the absence of TFA, while involving an equivalent amount, 60% of the desired product was isolated. These results revealed that TFA reacts with the aminal to form the electrophilic iminium ion *in situ*; interestingly, TFA activates the indole, generating the trifluoroacetylation intermediate 105, which can then facilitate the umpolung to form an enamine 106, enabling the subsequent C(sp^3^) H activation followed by the formation of the aminomethylated products.

**Scheme 48:**
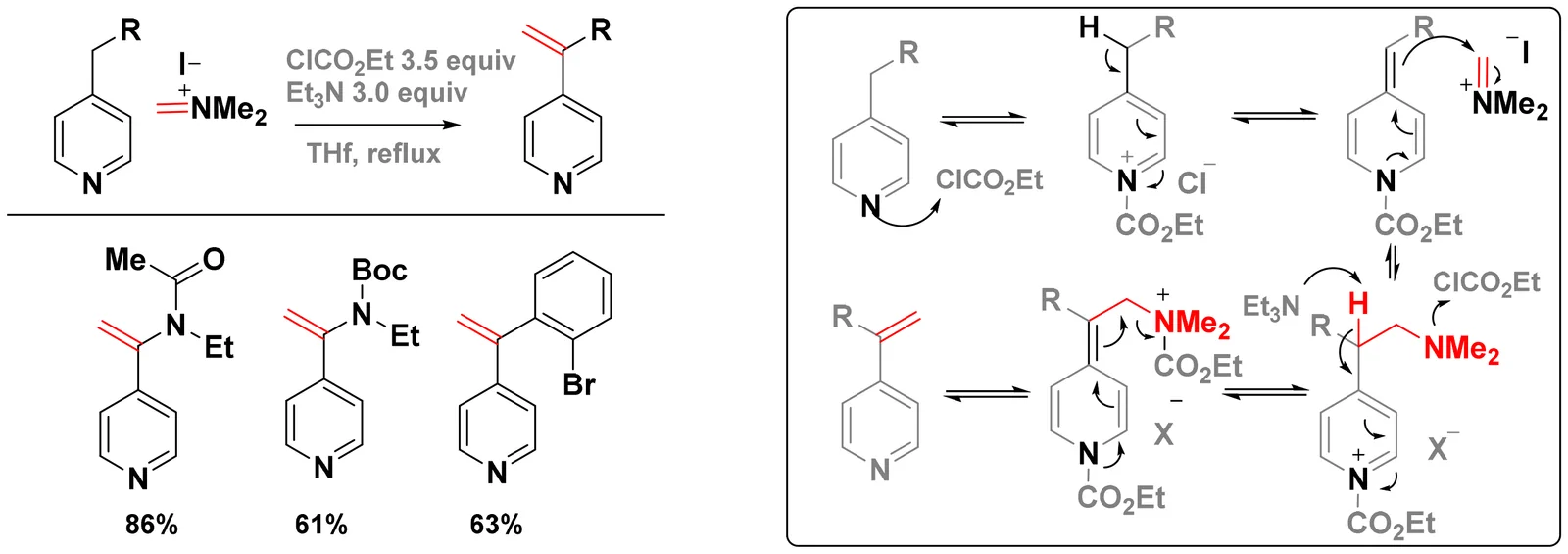
4-Alkylpyridine methenylation: a plausible mechanism.

Having in mind the relevance of conjugated vinyl and alkenyl pyridines as synthetic intermediates and highlighting the versatility of organometallic addition–elimination pathways for accessing alkenylated substrates,^116^ a sequential organometallic addition–dehydration pathway could enable the synthesis of disubstituted 4-alkenyl pyridines.^117^ Alternatively, a straightforward approach towards geminal 1,1-disubstituted 4-alkenyl pyridines involves the use of a suitable methenylating agent. The reaction of 4-(aminomethyl) pyridines and Eschenmoser's salt allowed the efficient synthesis of 4-alkylpyridine derivatives (Scheme 48). The reaction proceeded *via* alkylidene dihydropyridine intermediates, conveniently generated under mild conditions, using ethyl chloroformate (as a pyridine activating agent) in combination with triethylamine (Et_3_N). The plausible mechanism underlines the ability of the excess ethyl chloroformate to assist the elimination of the dimethylamino group originating from Eschenmoser's salt.^118^

The preformed iminium salts (Eschenmoser's salt and its chloride) proved to be effective for the introduction of the aminomethylene fragment into substituted 2-pyridones by employing microwave-assisted organic synthesis (MAOS).^119^ Salt S1 was irradiated for 9 min in 1,2-dichloroethane at 160 °C in the presence of pyridone 107a, resulting in the aminomethyleneted product 108a in 78% yield. Upon modifying the reaction conditions (using S2 for 400 s and at 140 °C), it was obtained in 92% yield. Similarly, satisfactory yields and short reaction times were also reported for more sterically hindered 2-pyridones 107b and 107c (Table 3), although in these cases, a better performance was observed employing the corresponding morpholine ammonium salt.

**Table 3:** Reactions between S2 and an aromatic compound under MW irradiation

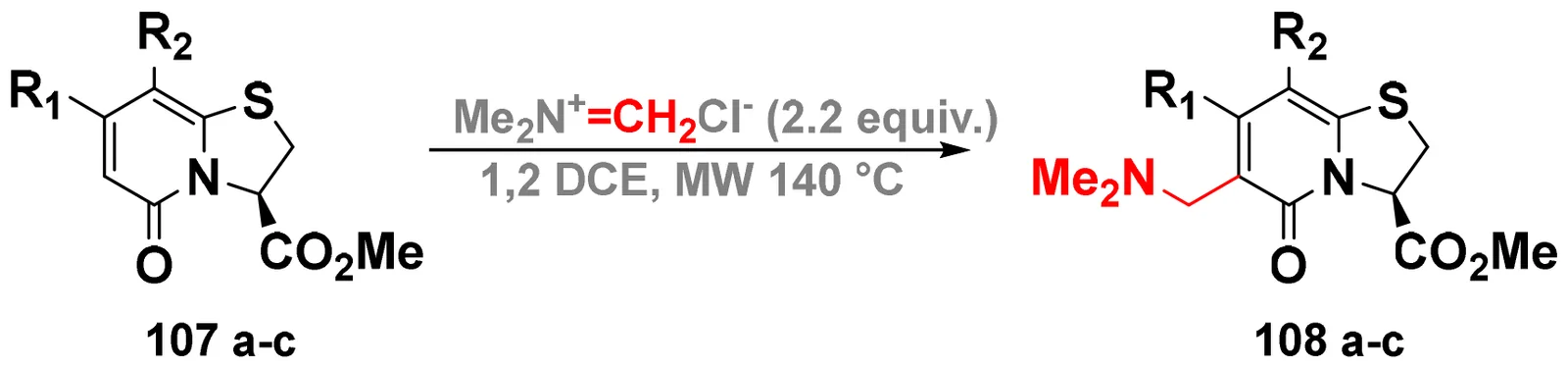		
Substrates	Time (s)	Yield (%)
R_1_ = Ph, R_2_ = Me	400	108a, 92%
R_1_ = Ph, R_2_ = CH_2_-1-naphthyl	800	108b, 48%
R_1_ = cyclopropyl, R_2_ = CH_2_-1-naphthyl	800	108c, 55%

**Scheme 49:**
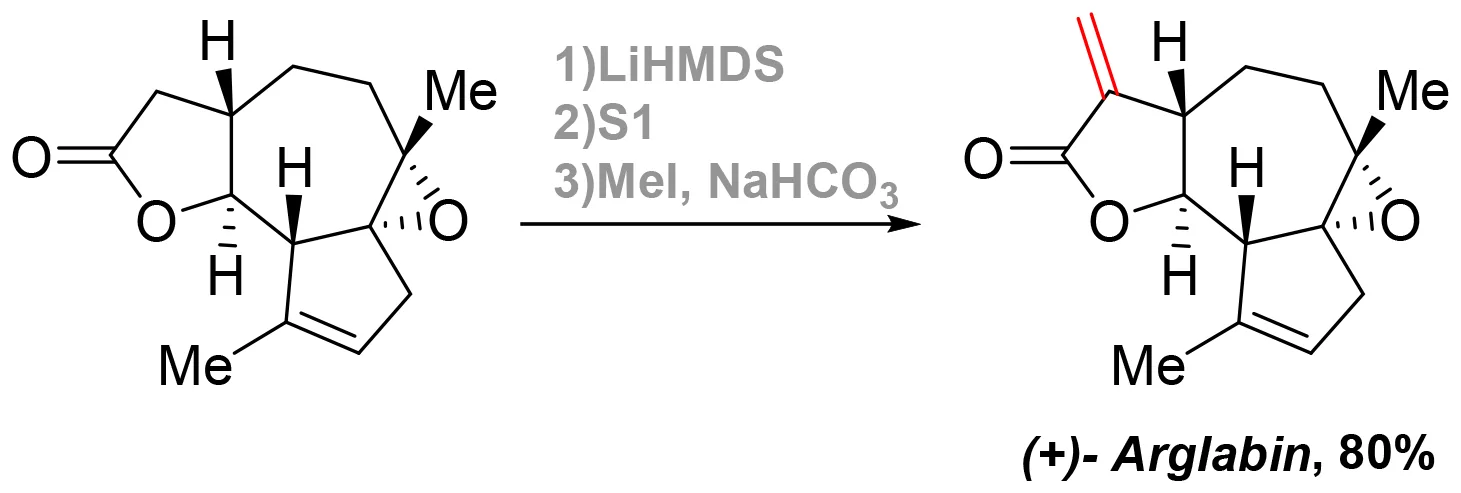
Eschenmoser's salt for the construction of an α-alkylidene lactone motif.

The high interest in the α-alkylidene lactone motif, typically constructed by the condensation of lactone enolates with aldehydes or iminium ions, as described in the synthesis of (+)-arglabin (Scheme 49),^120^ and found in numerous synthetically challenging and biologically important natural products, has prompted the development of one-pot assembly procedures.

**Scheme 50:**
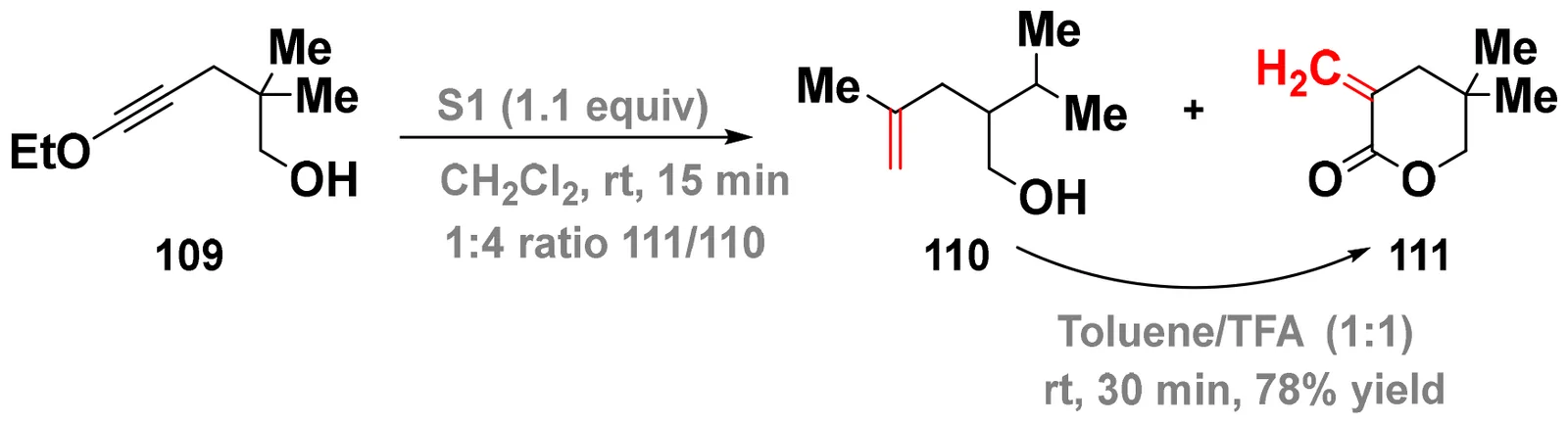
Reactions between Eschenmoser's salt and an alkynyl substrate.

In this context, Ng *et al.*^121^ showed that Eschenmoser's salt was able to react with alkynyl substrates, giving access to α-methylene lactone starting from an ynol ether *via* a tandem bond-forming reaction that would allow the rapid build-up of molecular complexity in a single step. Thus, the reaction of ynol ether 109 with S1 in CH_2_Cl_2_ at room temperature yielded a 1 : 4 mixture of α-methylene γ-valerolactone 111 and acyclic enoate 110, which resulted in a quantitative conversion to the α-alkylidene lactone 111 upon exposure to 1 : 1 TFA/toluene at room temperature for 30 min (Scheme 50).

Intriguingly, the methenylation of propargylic C–H bonds was also achieved using cyclopentadienyliron(ii) dicarbonyl complexes as catalysts and an iminium electrophile generated *in situ* from 2,2,6,6-tetramethylpiperidine, allowing the development of a protocol that is formally and mechanistically analogous to the Eschenmoser methenylation.^122^

**Scheme 51:**
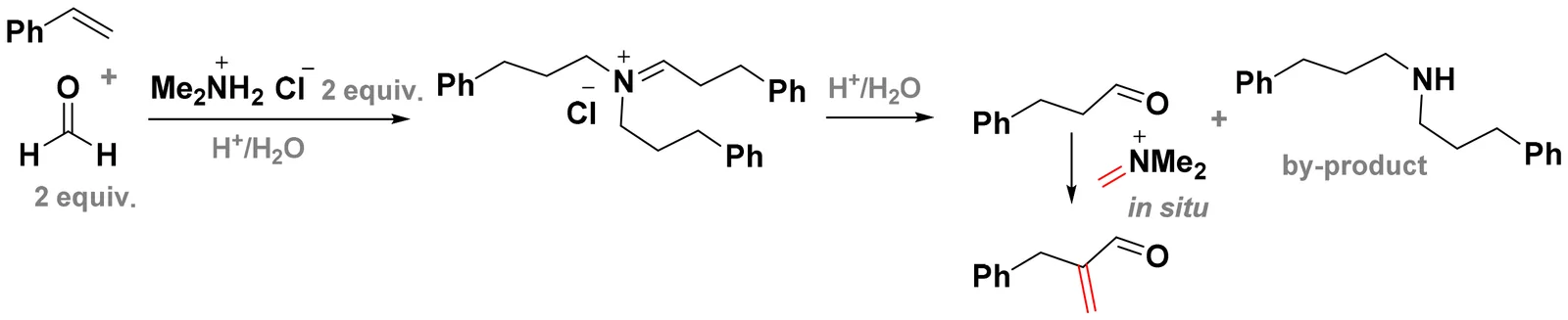
Reaction between Eschenmoser's salt and styrene.

Iminium ions have been reported to react with alkenes such as norbornene derivatives in the presence of formaldehyde.^123^ Despite this strategy leading to a mixture of products in most cases, in the late 1980s, Manninen^124^ reported a metal-free pathway *en route* to α,β-unsaturated aldehydes by reacting styrene, paraformaldehyde and dimethylammonium chloride in the presence of acetic acid, furnishing the targeted compounds in yields up to 50% (Scheme 51). The reaction of an alkene with an *in situ* formed Eschenmoser's salt led to the formation of a tertiary iminium cation, which can be hydrolyzed to yield an aldehyde and a secondary amine; subsequently, the aldehyde reacted with the iminium ion, yielding the targeted α,β-unsaturated products. Despite being attractive, this pathway has significant shortcomings, such as the stoichiometric use of S2 and a poor atom economy due to the formation of a secondary amine as a byproduct.

**Scheme 52:**
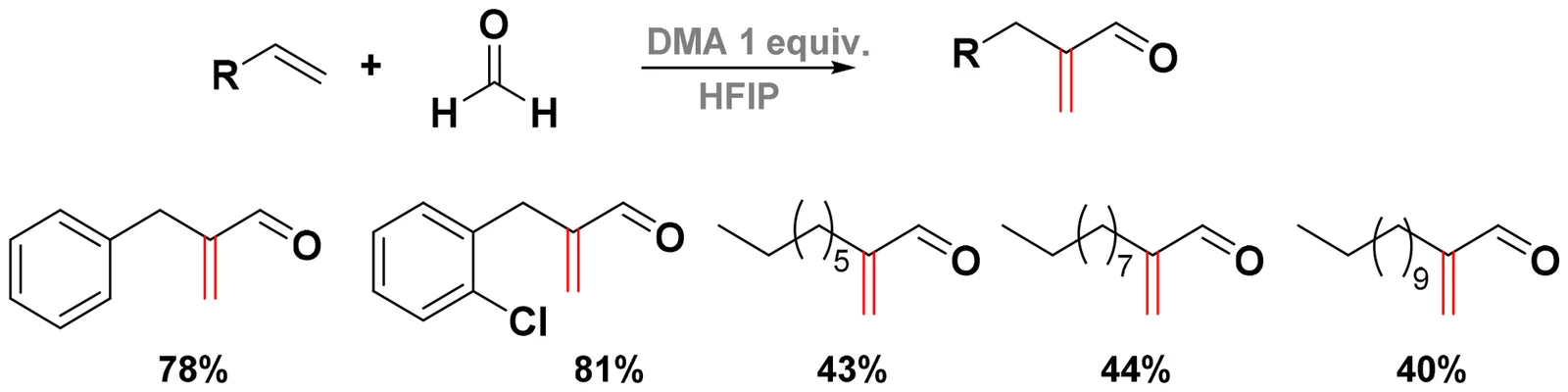
Metal-free pathway to α,β-unsaturated aldehydes.

An alternative methodology for this reaction involved a one-pot, metal-free pathway without the formation of the secondary amine as a co-product by employing dimethylamine (DMA) for the *in situ* generation and regeneration of Eschenmoser's salt (Scheme 52). The reaction of styrene, formalin and one equivalent of DMA in HFIP for 20 h afforded the corresponding 2-benzylacrolein in 78% yield. The methodology was also applicable to less reactive linear alkenes such as 1-octene, 1-decene and 1-dedecene.^125^

**Scheme 53:**
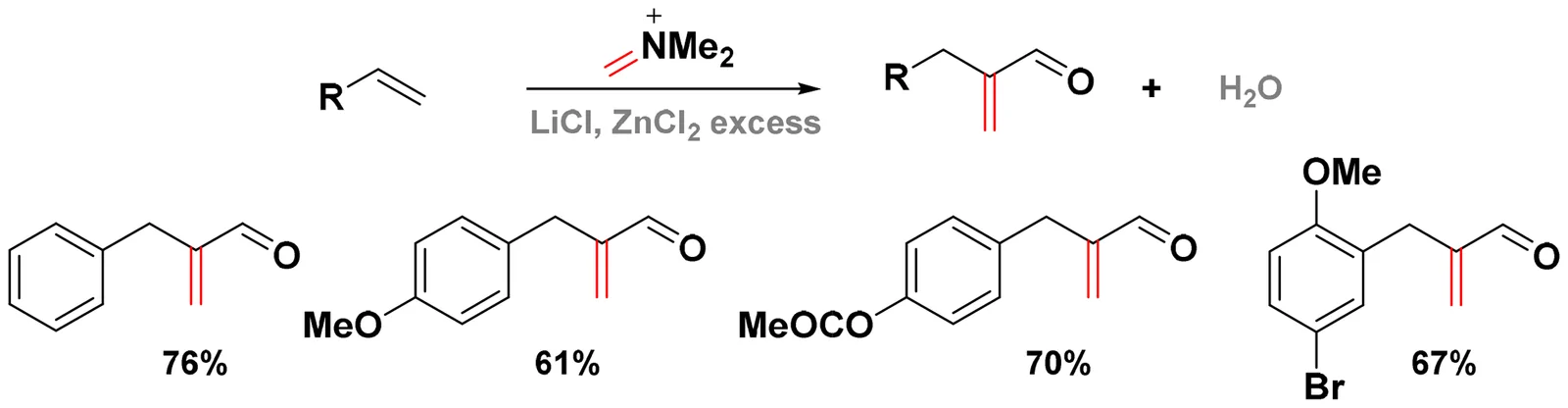
ZnCl_2_/LiCl/H_2_O-mediated transformation of styrenes.

Notably, a recent study by Fedorov *et al.*^126^ has demonstrated that iminium ions react with styrenes *via* a ZnCl_2_/LiCl/H_2_O-mediated transformation providing α,β-unsaturated aldehydes in yields up to 60% (Scheme 53).

**Scheme 54:**
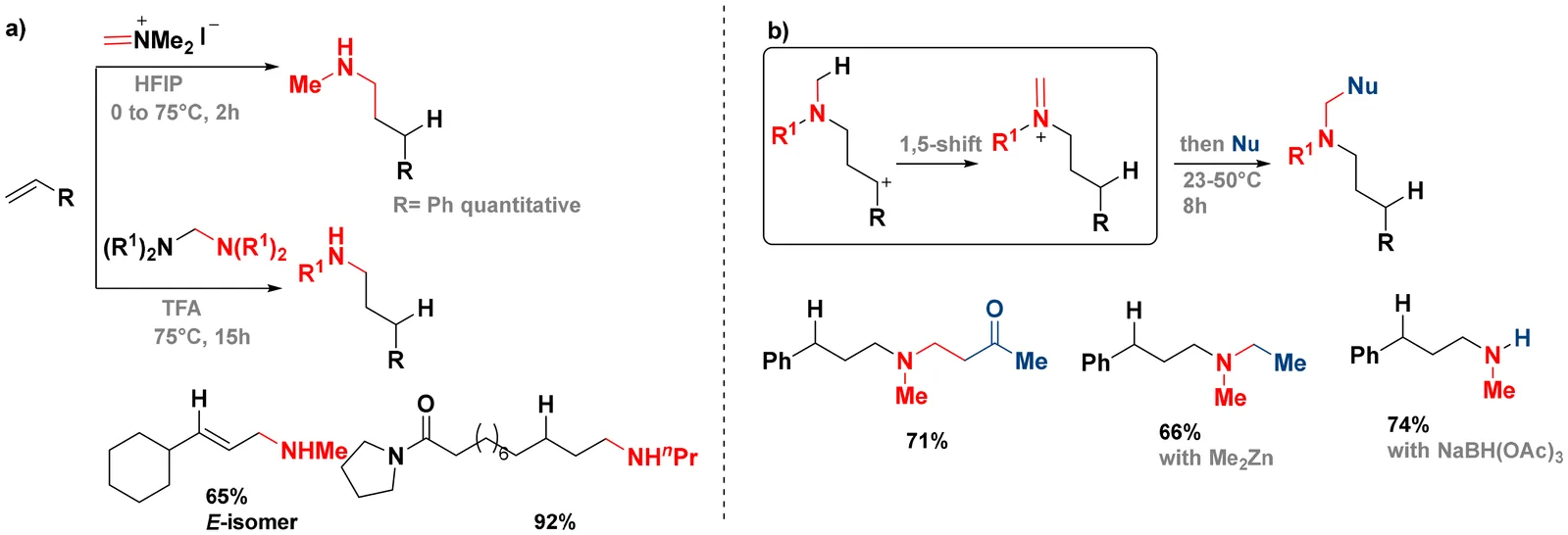
Reactions between Eschenmoser's salt and π-systems.

The nucleophilic addition of a π-system to an iminium ion, followed by an internal 1,5-hydride transfer of the resulting carbenium ion, affords hydroaminomethylated products or, upon nucleophile addition, directly functionalized derivatives. Hence, Maulide *et al.*^127^ reported Eschenmoser's salt reacting with styrene in HFIP to yield quantitatively the corresponding linear amine (Scheme 54a). Expanding the substrate scope, alternative iminium ion precursors such as the commercially available aminals in TFA at 75 °C furnished secondary amines in high yields after aqueous hydrolysis. For terminal alkynes, the reaction provided secondary allylic amines exclusively as the *E*-stereoisomers, consistent with the *syn* nature of the hydride transfer. The iminium ion formed *via* a 1,5-hydride shift (Scheme 54b) can also be engaged in domino C–C or C–H functionalization to afford trisubstituted or tertiary benzylic amines, or in hydroaminomethylation–functionalization sequences with organozinc nucleophiles affording the corresponding amines in up to 60% yield.

**Scheme 55:**
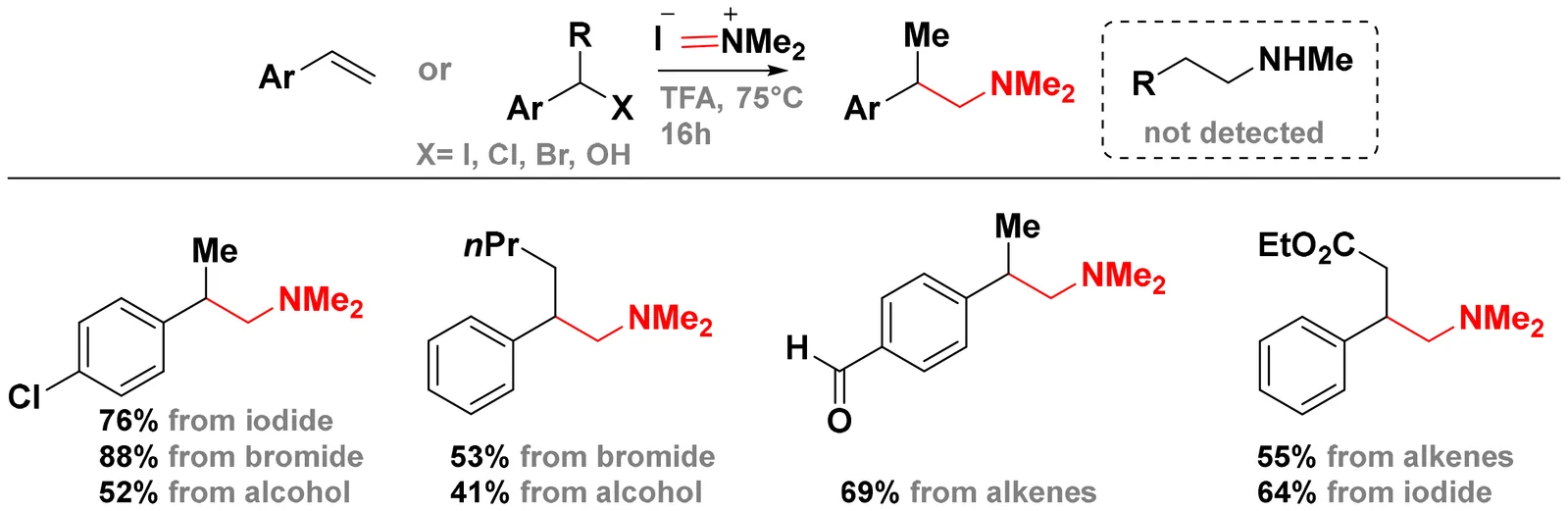
Reactions with Eschenmoser's salt in TFA.

Interestingly, when the reaction of Eschenmoser's salt with styrene was carried out in TFA rather than HFIP at the same temperature, a branched dimethylated product was obtained (Scheme 55).^128^ The transformation proceeded smoothly with styrenes, benzyl halides, benzyl alcohols and even cinnamic esters, affording the corresponding aminomethylenated products in very good yields and with excellent chemoselectivity.

**Scheme 56:**
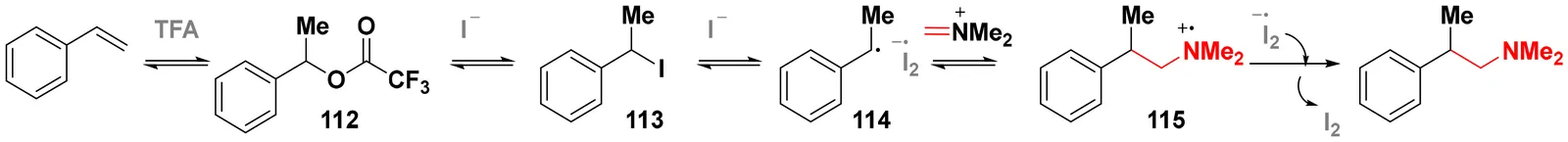
Proposed reaction mechanism based on experimental and computational results.

Testing iminium ions bearing counteranions other than iodine (chloride, bromide or triflate) revealed the exclusive reactivity of S1, as no product formation was observed in these cases. This result may be attributed to the reducing ability of iodide. Indeed, experimental and computational data suggested that the reaction proceeds *via* the formation of a TFA-adduct 112 (Scheme 56),^128^ which, in the presence of iodide, generates the corresponding benzylic iodide 113. Homolytic cleavage of the C–I bond affords a diiodide radical complex and a radical species 114 capable of adding to Eschenmoser's salt to form a new C–C bond, yielding the aminium radical cation 115. Subsequent single-electron transfer furnishes the reduced tertiary amine and molecular iodine (I_2_) as the only by-product.

**Scheme 57:**
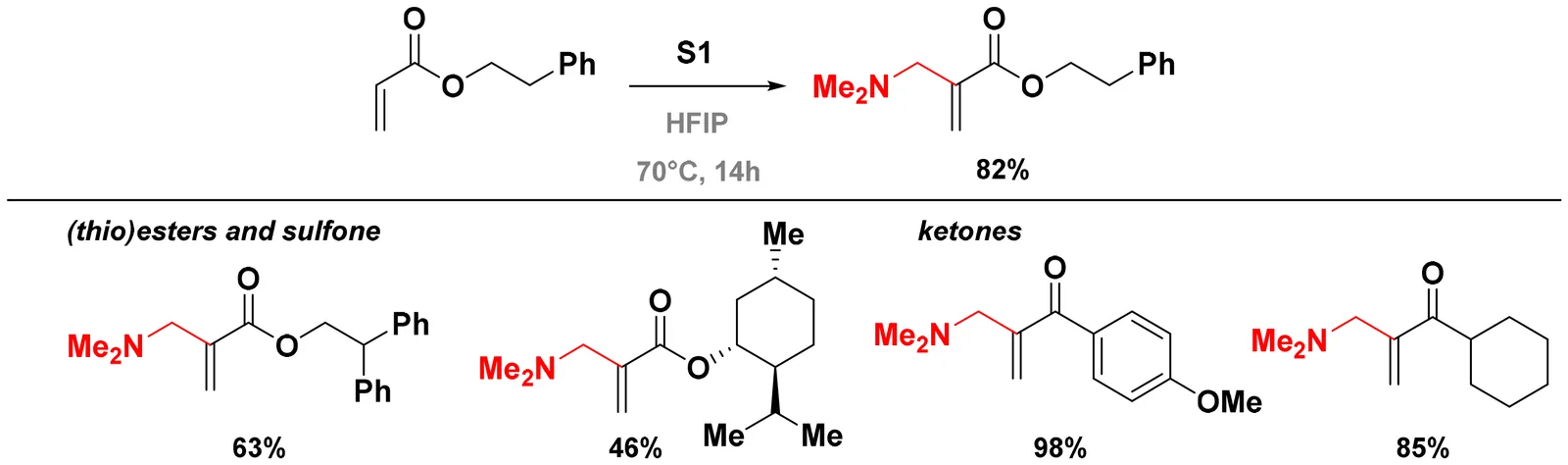
MBH-type coupling of Michael acceptors and Eschenmoser's salt.

Among the many available methods for C–C bond formation,^129,130^ α-functionalized alkenes activated by electron-withdrawing groups (EWGs) are versatile substrates indeed. The Morita–Baylis–Hillman (MBH) reaction and its aza-variation, which employs imine derivatives as electrophiles, have attracted particular interest in the synthesis of β-amino carbonyl compounds. While these processes typically require a Lewis base catalyst, a catalyst-free α-aminomethylation of Michael acceptors was recently reported using iminium ions.^131^ In this context, Böhme's salt S2 was completely unreactive, whereas Eschenmoser's salt S1 reacted with homobenzylacrylate affording the desired aza-MBH adduct in 82% yield (Scheme 57).

The combination of the iodide counterion and HFIP as a solvent proved crucial for mediating the reaction. Computational studies, examining solvent interactions with S1 or S2, highlighted the strong effect of HFIP in facilitating halide dissociation, with a halogen–carbon distance of 0.528 Å for Böhme's salt and 0.840 Å for Eschenmoser's salt. Computed Gibbs free energies suggested that dissociation of S1 in HFIP is thermodynamically favorable (Δ*G* = 7.2 kcal mol^−1^), whereas it is highly reversible for S2 (Δ*G* = 0.5 kcal mol^−1^). The observed halide effect was thus attributed to the higher dissociation probability of Eschenmoser's salt. Investigation of the scope with various Michael acceptors allowed the isolation of a broad range of products in high yields, as shown in Scheme 57 (*infra*).

**Scheme 58:**
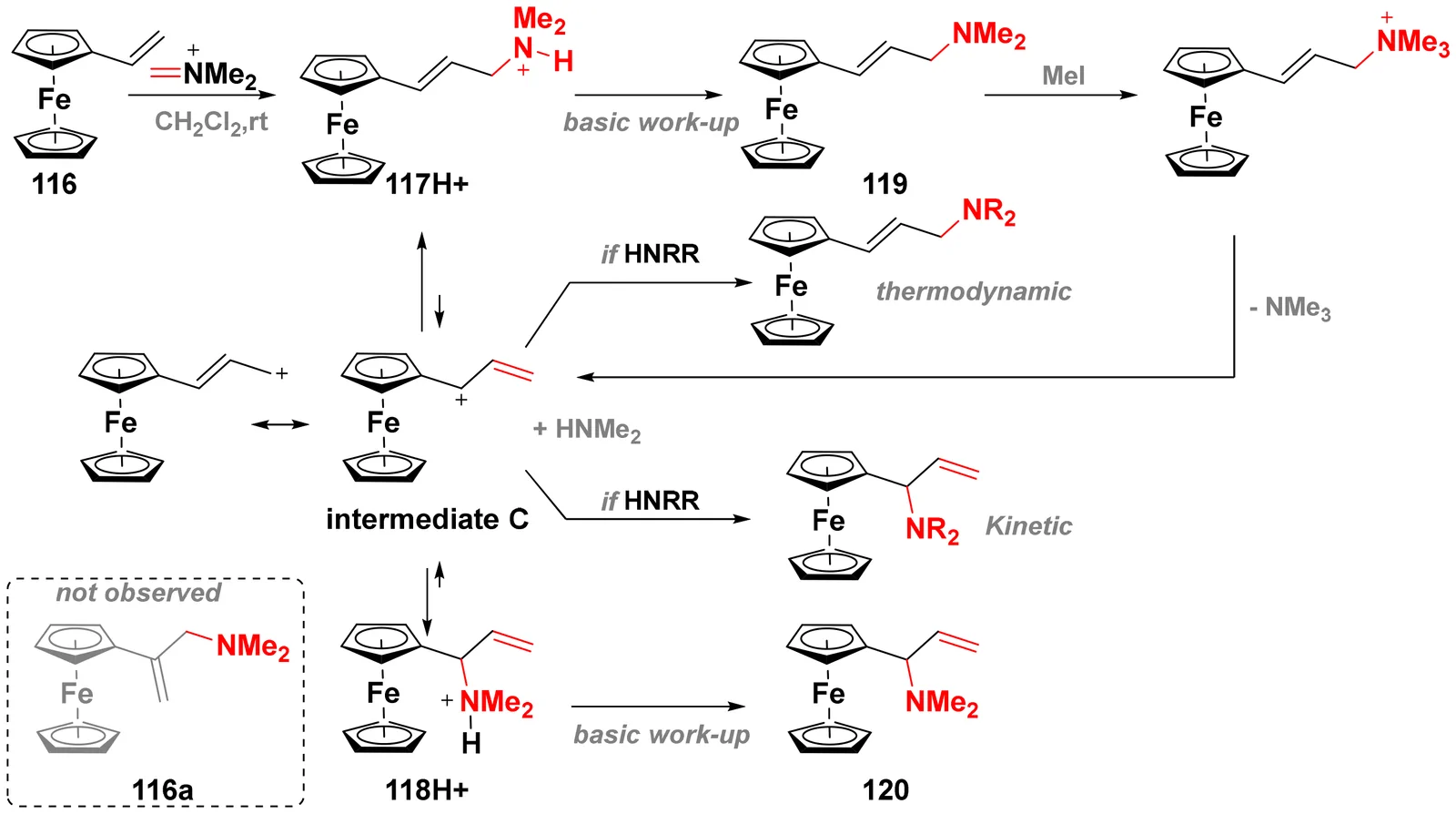
Reactions between Eschenmoser's salt (S1) or its chloride analogue S2 and vinylferrocene.

Considering the strong ability of ferrocene to stabilize carbocations in α-positions, Bouchene *et al.*^132^ assumed that vinylferrocene would be able to react with Eschenmoser's salt or other dimethylmethylenammonium salts allowing the formation of ferrocene-containing allylamines (Scheme 58). The reaction of commercially available Eschenmoser's salt S1, or its chloride analogue S2, with vinylferrocene yielded, after 16 h, the linear allylic amine 119 with perfect regio- and stereoselectivity. Under the same conditions, when the reaction time was limited to 5 hours, complete consumption of 116 yielded product 119 along with traces of isomer 120. While the branched isomer 116a (resulting from the attack at the α-position of the ferrocene moiety) was not observed, the formation of by-product 120 was rationalized as arising from the rearrangement of ammonium intermediate C (present before the basic workup), in which the positive charge is located on the carbon atom α to the ferrocene moiety. Starting from C, the ammonium species 117H+ is thermodynamically favored, while 118H+ slowly equilibrated to the stable ammonium form, yielding only product 119 after 16 h. This key observation opened the doors to a series of new ferrocenylallyl amines *via* nucleophilic substitution of the resulting ammonium salt with primary and secondary amines. Notably, the regioselectivity of the substitution, yielding the kinetic (branched allylamines) or the thermodynamic products (linear allylamines), can be tuned by simply adjusting the amount of base in the reaction media.

**Scheme 59:**
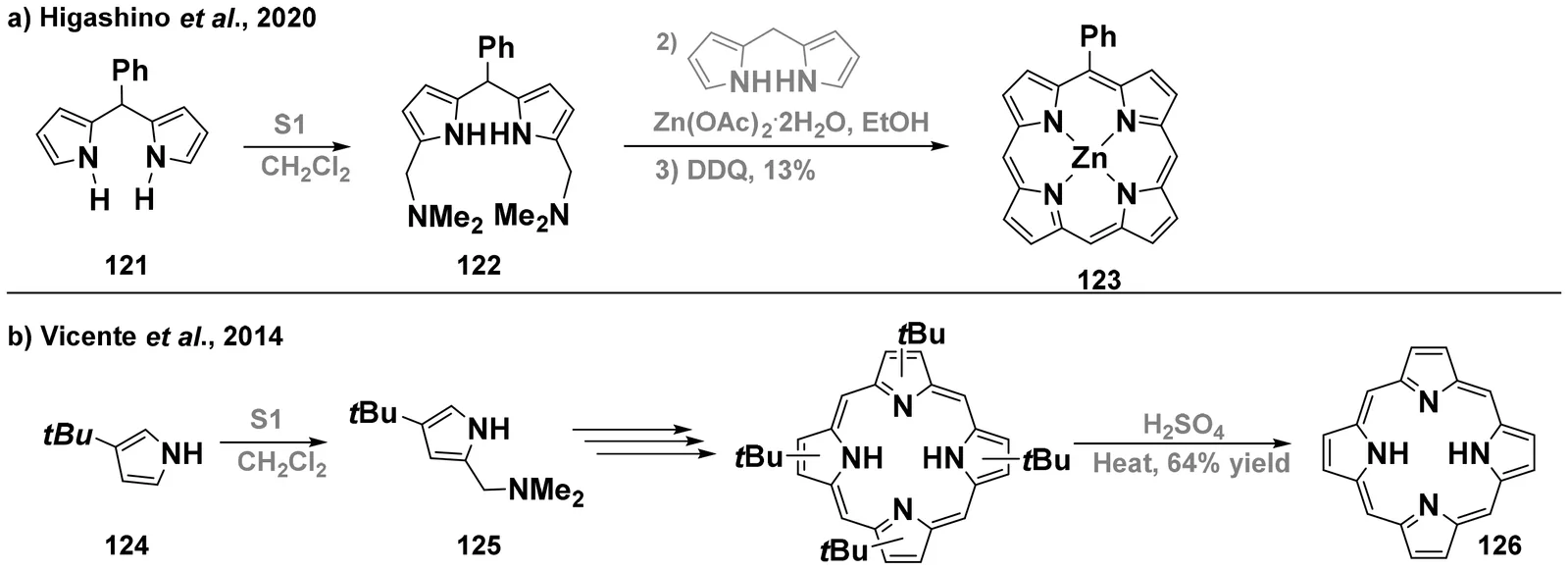
Synthesis of two porphyrins using Eschenmoser's salt as a starting material.

Considering the growing interest in developing building blocks for organic functional materials used in organic light-emitting diodes (OLEDs) and organic solar cells (OSCs), porphyrins (in particular *meso*-aminoporphyrins) have attracted increasing attention as efficient sensitizers for dye-sensitized solar cells (DSSCs). In this regard, porphyrin dyes bearing multiple *meso*-diarylamino groups are associated with unique optical and electrochemical properties. Among the currently available synthetic procedures for their preparation, the use of Eschenmoser's salt has been widely exploited in the synthesis of this class of macrocycles. Thus, monosubstituted 123 was synthesized by the reaction of S1 with 5-phenyldipyrromethane 121, obtaining the desired intermediate 122, which in the presence of 2,2′-dipyrromethane and Zn(OAc)_2_ afforded the desired porphyrinic structure in 13% yield (Scheme 59a).^133^

Initial attempts to synthesize the unsubstituted porphyrin 126 directly from pyrrole and formaldehyde resulted in low yields, primarily due to the poor solubility of the target compound (Scheme 59b). In contrast, the use of Eschenmoser's salt enabled the formation of the pyrrolic intermediate 125, which could be efficiently converted into soluble tetra-*tert*-butylporphyrin. Subsequent removal of the *tert*-butyl groups afforded the desired octaethylporphyrin in 64% yield, thereby circumventing the solubility limitations associated with 126.^134^

### 4.5. Addition to active methylene compounds

Active methylene compounds feature a methylene group flanked by two electron-withdrawing functional groups. This arrangement stabilizes the conjugate base *via* resonance, increasing acidity and facilitating deprotonation. Methylene salts readily react with such compounds.

**Scheme 60:**
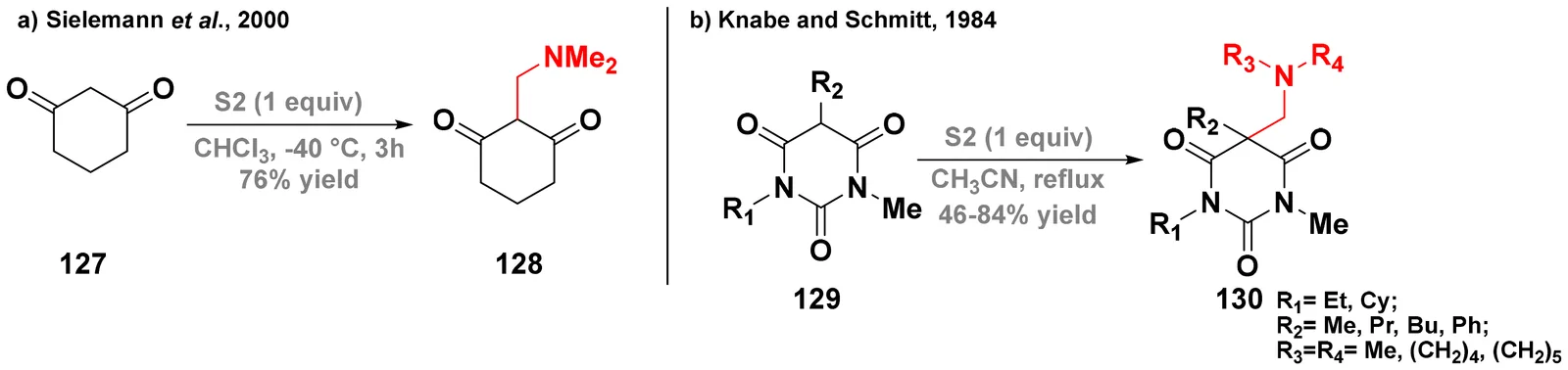
Typical reaction of the iminium salt with an active methylene compound.

For example, Böhme's salt S2 can react with cyclohexane-1,3-dione 127 to afford the corresponding (dimethylamino)methyl product 128 in good yield (Scheme 60a).^135^ Similarly, S2 can also react with more functionalized substrates, as reported by Knabe and Shmitt,^136^ affording a series of barbituric derivatives in reasonable yields (Scheme 60b).

**Scheme 61:**
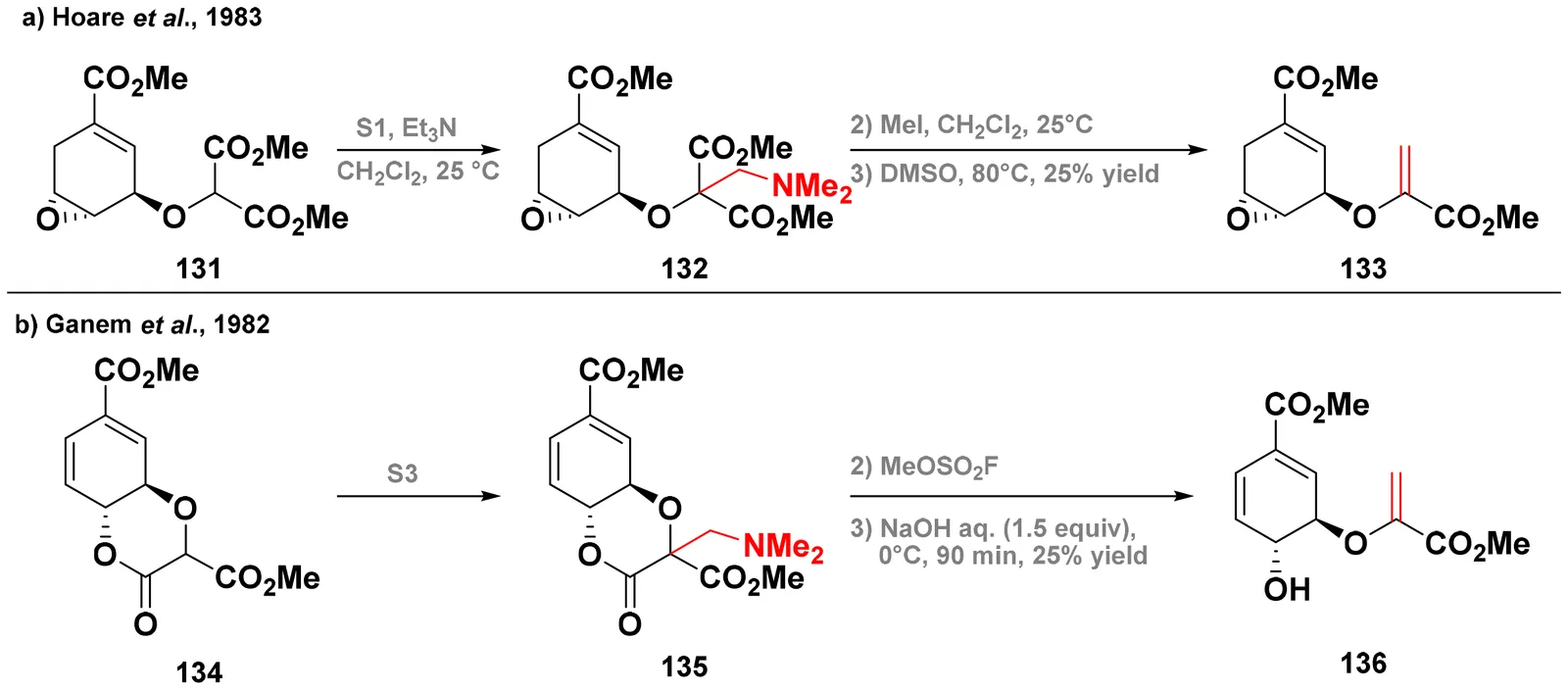
Reactions between Eschenmoser's salt and activated esters.

For less reactive compounds, such as esters, activation of the methylene group is also an efficient strategy to boost the acidity of the α-protons. For example, α-alkoxy malonate 131 reacted with an iminium salt to provide the Mannich base 132, which upon quaternization and thermal fragmentation yielded enol pyruvate 133 (Scheme 61a).^137^ Despite the mild reaction conditions preserving sensitive functionalities, such as epoxides, for more sensitive substrates, such as cyclohexadiene 134, salt S3 was used to avoid possible aromatization, probably triggered by iodide attack on the ring. The resulting Mannich base 135 is then directly converted to the desired product 136 upon base-induced fragmentation (Scheme 61b).^138^

**Scheme 62:**
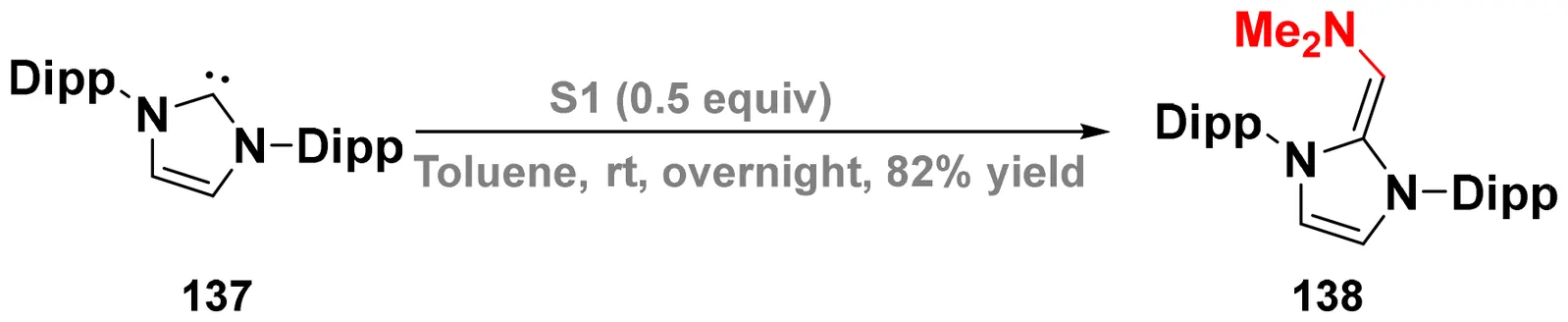
Synthesis of an NHO amine from S1 and an N-heterocyclic carbene (NHC).

Interestingly, the synthesis of N-heterocyclic olefine (NHO) amines can also be achieved, as described by Paisley *et al.*,^139^ through reacting Eschenmoser's salt with N-heterocyclic carbenes (NHCs) (Scheme 62). The dimethylamino-substituted NHO, compound 138, was then obtained from the combination of two equivalents of the commercially available carbene IPr 137 with one equivalent of Eschenmoser's salt in toluene. Regarding the mechanism of this reaction, the first equivalent of IPr underwent nucleophilic attack on the iminium salt to form the (dimethylamino)methyl derivative, which was subsequently deprotonated by the second equivalent to yield the desired product 138.

### 4.6. Reactions of iminium salts with organometallic reagents

The widespread use and application of organometallic reagents in organic chemistry^140,141^ also extends to their reactions with iminium salts. These highly electrophilic imine derivatives can readily undergo nucleophilic addition with the carbon–metal bond of organometallic reagents, enabling the formation of new C–C bonds. Subsequent hydrolysis of the resulting adduct affords secondary or tertiary amines, depending both on the substituents present on the iminium salt and on the nature of the organometallic reagent. In particular, lithium, zinc, and magnesium (Grignard) reagents are the most widely employed organometallic compounds for these transformations. Depending on the nature of the metal, the balance between reactivity and selectivity can be finely tuned, providing access to a diverse array of amine derivatives and related nitrogen-containing compounds of synthetic and pharmaceutical relevance.

**Scheme 63:**
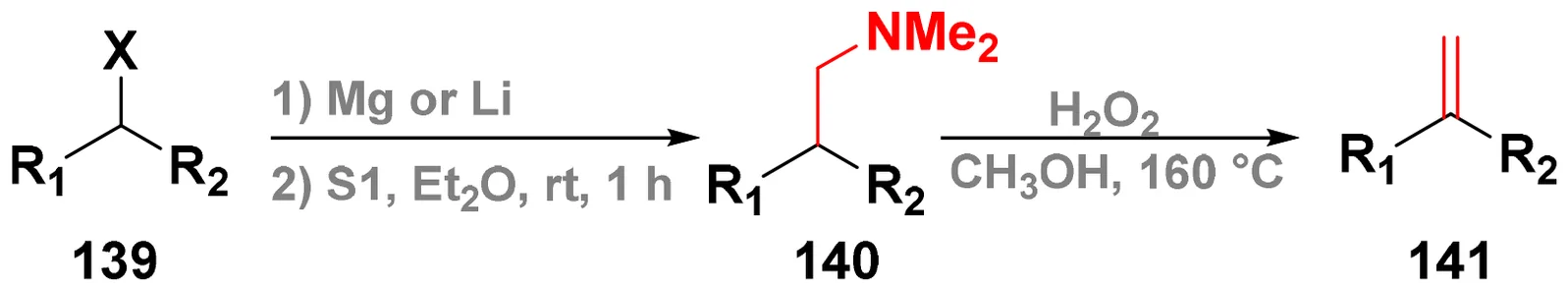
Typical reaction between Eschenmoser's salt and an organometallic reagent.

One of the earliest applications of organometallic reagents with Eschenmoser's salt dates back to 1977, when Poulter *et al.*^13^ reported the employment of lithium or magnesium reagents (prepared through the now well-known halogen–metal exchange procedures from halide 139) in reaction with S1. The reaction enabled access to functionalized β-amines 140, which, upon oxidation with hydrogen peroxide, afforded terminal olefins 141 in high yields (Scheme 63).

**Scheme 64:**
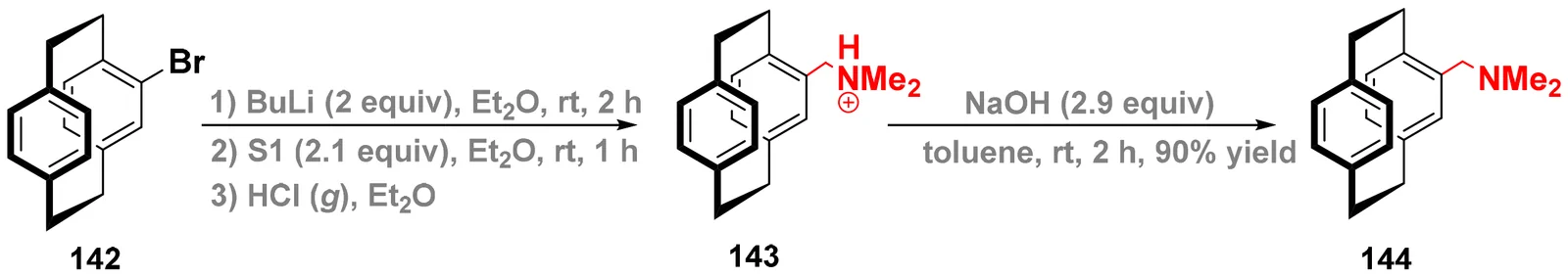
Reaction between Eschenmoser's salt and 4-bromo [2.2]paracyclophane.

Similarly, the bromide substrate 142 previously treated with BuLi reacts with Eschenmoser's salt affording the aminomethylated product 144 in moderate yield after a basic workup (Scheme 64).^142^

**Scheme 65:**
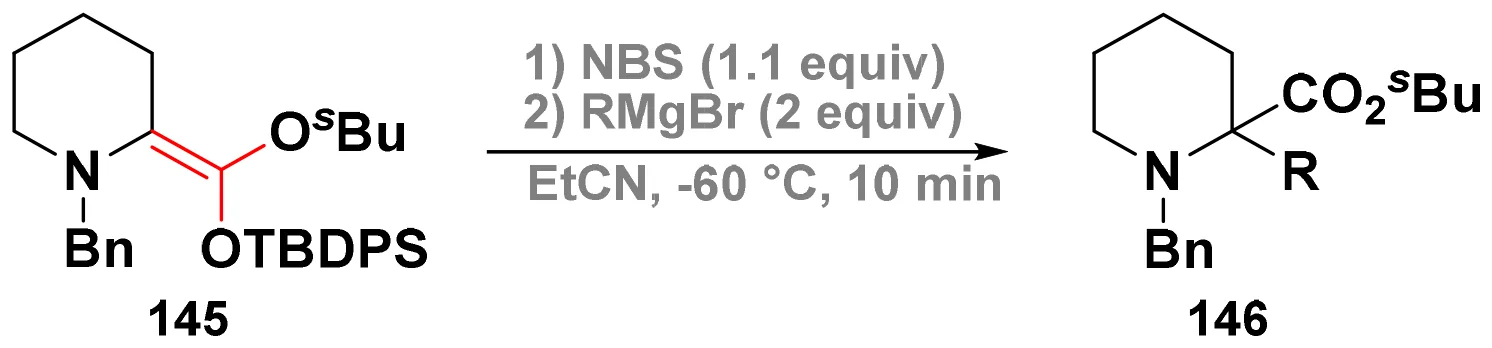
Reaction of an *in situ* formed iminium salt and a Grignard reagent.

Considering the high electrophilicity of ketene species which can be rapidly intercepted by nucleophiles,^143^ Kawamura *et al.*^144^ reported the nucleophilic addition of Grignard reagents to a cyclic amino ketene silyl acetal derived from pipecolic acid 145 (Scheme 65). Notably, oxidation of 145 with NBS (*N*-bromosuccinimide) generated an iminium ion, which readily underwent reaction with the Grignard reagent to afford α-quaternary amino ester derivatives 146.

Primary and secondary aliphatic Grignard reagents, as well as aromatic analogues, reacted efficiently with the iminium salts, providing the desired products in good to excellent yields (Table 4 entries 1–7, 11, and 12). Alkenyl and alkynyl Grignard reagents also delivered the corresponding products in good yields (Table 4 entries 13 and 14). Moreover, reagents bearing acetal, ether, trimethylsilyl (TMS), or olefin functionalities were well tolerated under the reaction conditions (Table 4 entries 5–10).

**Table 4:** Reaction of an iminium salt and Grignard reagents

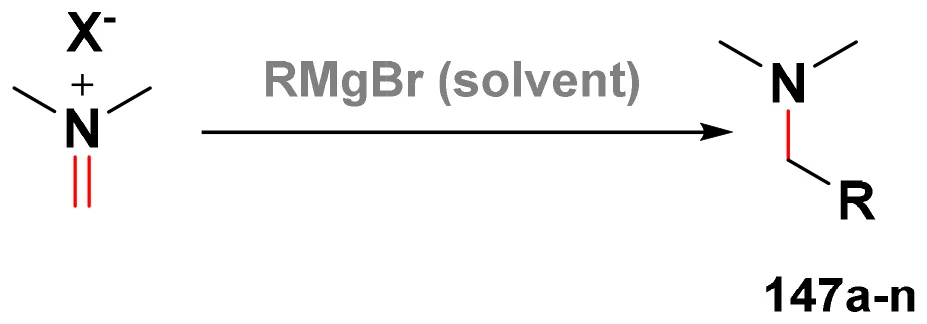			
Entry	Grignard reagent	Product	Yield (%)
1	EtMgBr (Et_2_O)	147a	86
2	*n*PrMgBr (Et_2_O)	147b	80
3	*n*OctMgBr (Et_2_O)	147c	79
4	BnMgBr (Et_2_O)	147d	69
5	TMSCH_2_MgBr (Et_2_O)	147e	70
6	CyMgBr (Et_2_O)	147f	40
7	iPrMgBr (Et_2_O)	147g	66
8	CH_2_=CHCH_2_CH_2_MgBr (Et_2_O)	147h	80
9	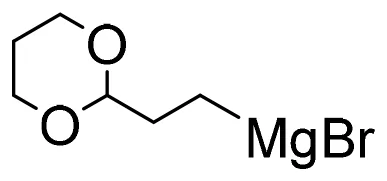	147i	74
10	BnO(CH_2_)_4_MgBr (Et_2_O)	147j	29
11	PhMgBr (Et_2_O)	147k	53
12	2-ThienylMgBr (Et_2_O)	147l	57
13	CH_2_=CHMgBr (THF)	147m	58
14	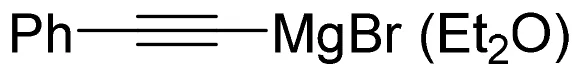	147n	65

**Scheme 66:**
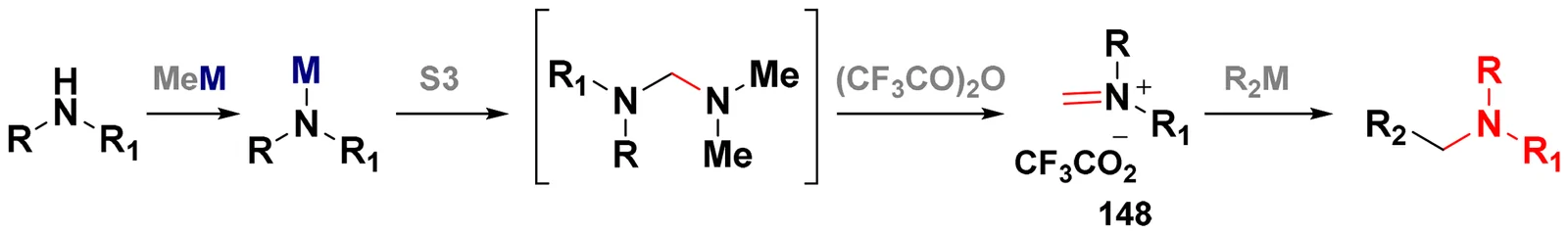
Reaction of an *in situ* formed iminium salt and organometallic reagents.

**Scheme 67:**
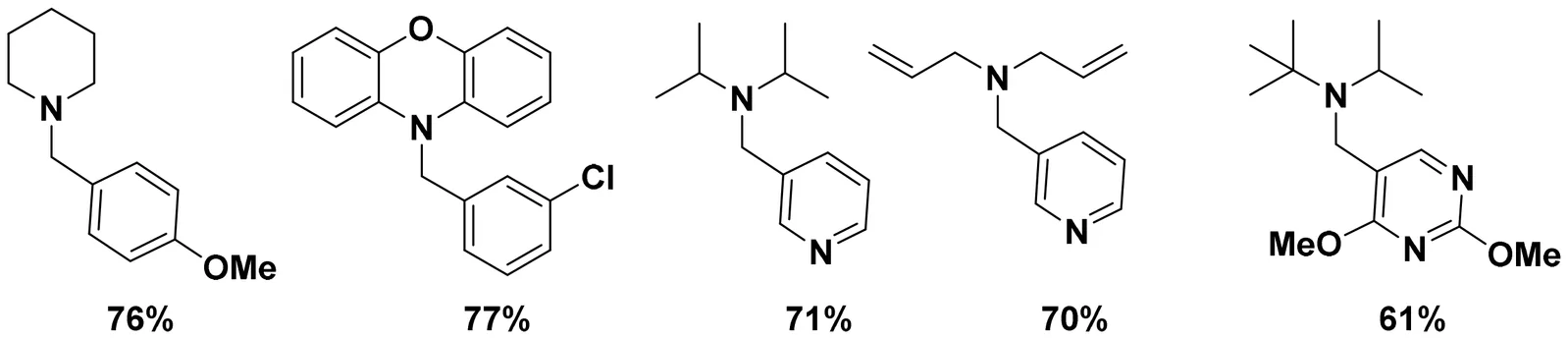
Reaction of an *in situ* formed iminium salt and a Grignard reagent.

The addition of organometallic reagents to iminium ions constitutes a useful synthetic strategy for tertiary amines (Scheme 66). In this context, Knochel *et al.*^29,145^ reported a one-pot homologative amination taking advantage of the ability of organometallic reagents to promptly react with trifluoroacetate Potier–Tietze salts, affording a series of highly functionalized amines. Thus, deprotonation of the starting amine with an organometallic species (MeLi or MeMgX) furnished the corresponding metallic amide, which promptly reacts with Potier–Tietze salt S3 affording an aminal, which directly undergoes disproportionation under mild acidic conditions with TFAA providing the complex iminium ions 148. The resulting iminium salt 147 is capable of reacting with several organometallics leading to polyfunctional tertiary amines (Scheme 67). A series of aryl and heteroaryl Grignard reagents proved to be effective in the process affording the desired highly functionalized benzylamines in high yields.^29^

**Scheme 68:**
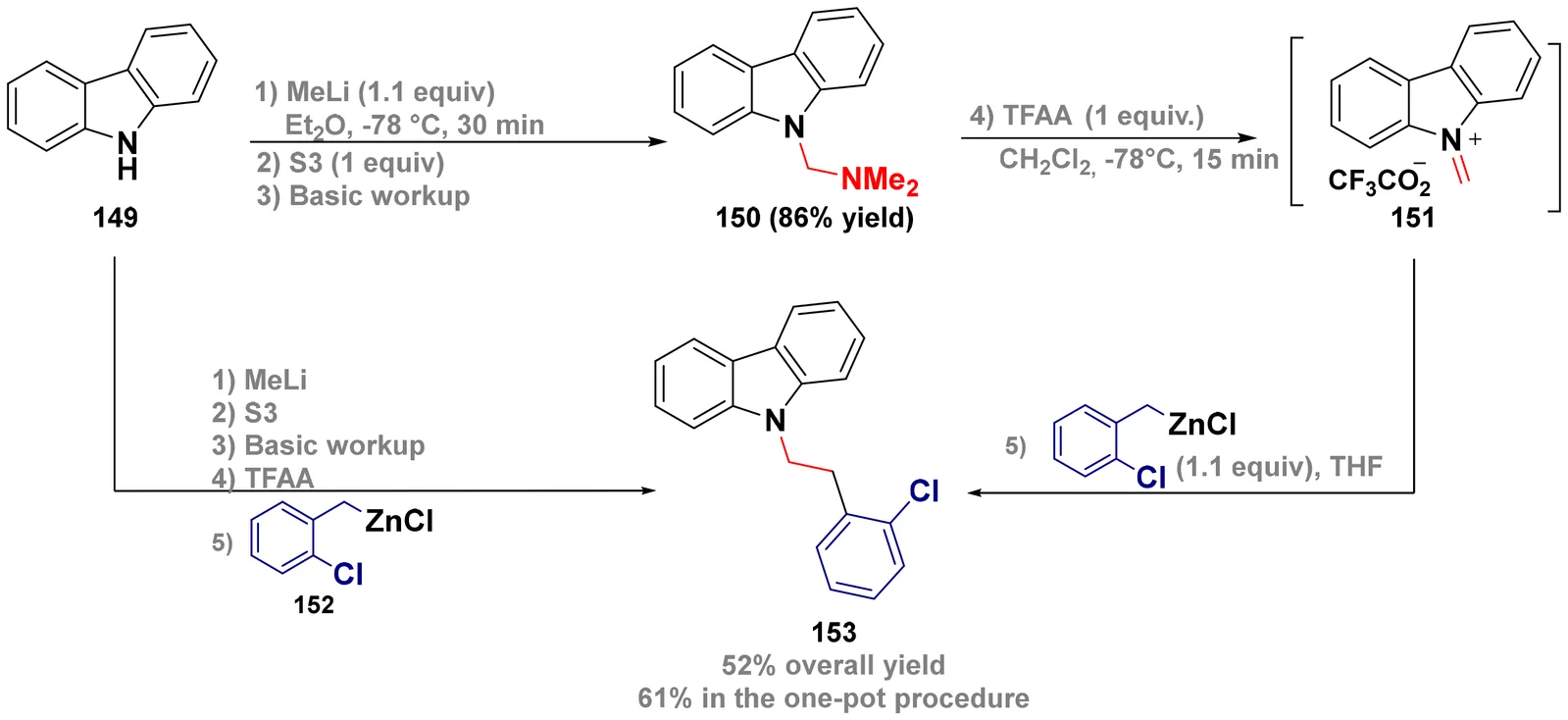
Preparation of tertiary amines starting from the trifluoroacetate methylene-iminium salt S3.

Interestingly, the strong electrophilic behavior of iminium salts allows relatively weaker nucleophiles, such as organozinc reagents, to attack efficiently the iminium reagent.^1^ Thus, Knochel's one-pot approach^29^ was also applied using organozinc reagents. For instance, 9*H*-carbazol 149, previously treated with MeLi to provide the corresponding lithium amide, reacted with iminium trifluoroacetate S3 affording the mixed aminal 150 (isolable after basic workup) which, in the presence of TFAA, formed a new iminium trifluoroacetate 151, able to undergo reaction with the organozinc reagent 152 affording the tertiary amine 153 in 52% yield (Scheme 68). The methodology proved its applicability to a range of functionalized secondary amines as well as a variety of organozinc reagents.

**Scheme 69:**
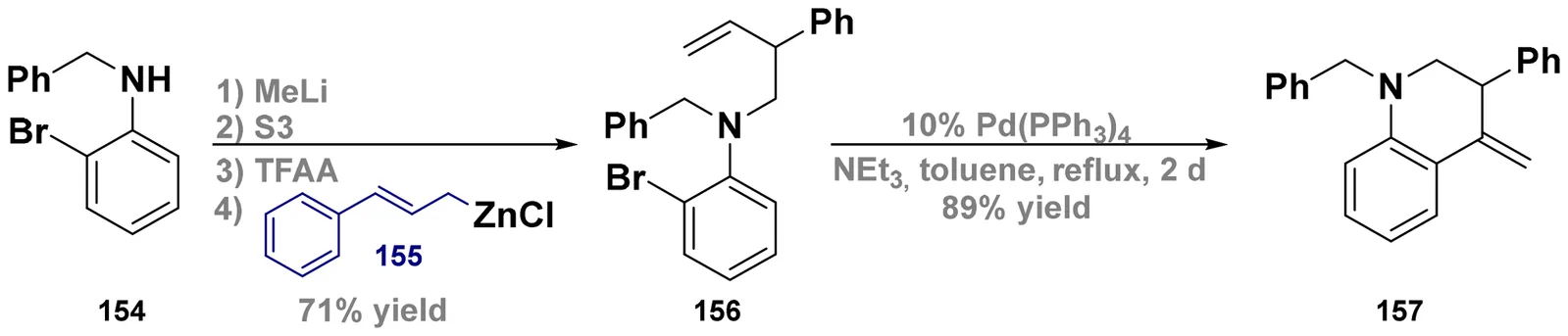
Reaction of salt S3 with an amine and a subsequent reaction with an organozinc compound.

The high functional group tolerance of this procedure enabled the synthesis of functionalized precursors suitable for cyclization reactions. This was demonstrated for amine 154 which, affording the corresponding intermediate iminium ion, reacted regioselectively with cinnamylzinc chloride 155 to deliver the polyfunctional aniline 156 (71% yield). The subsequent Heck cyclization of 156 led selectively to the *exo*-methylene quinolidine 157 in 89% yield (Scheme 69).

**Scheme 70:**
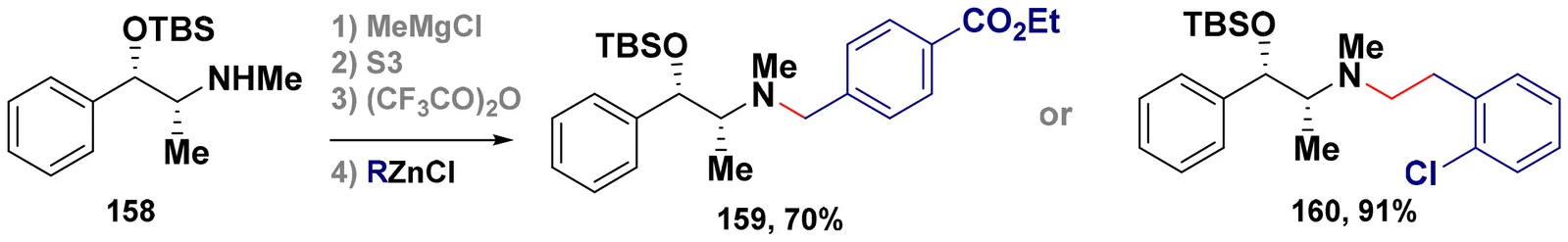
Reaction of the (+)-ephedrine derivative.

Notably, this homologative amination allowed the conversion of the (+)-ephedrine derivative 158 to benzylic and phenethylic amines 159 and 160 in 70–91% yields (Scheme 70), proving not only the broad applicability of this procedure but also how the development of new direct methodologies is highly beneficial for medicinal chemistry.

**Scheme 71:**
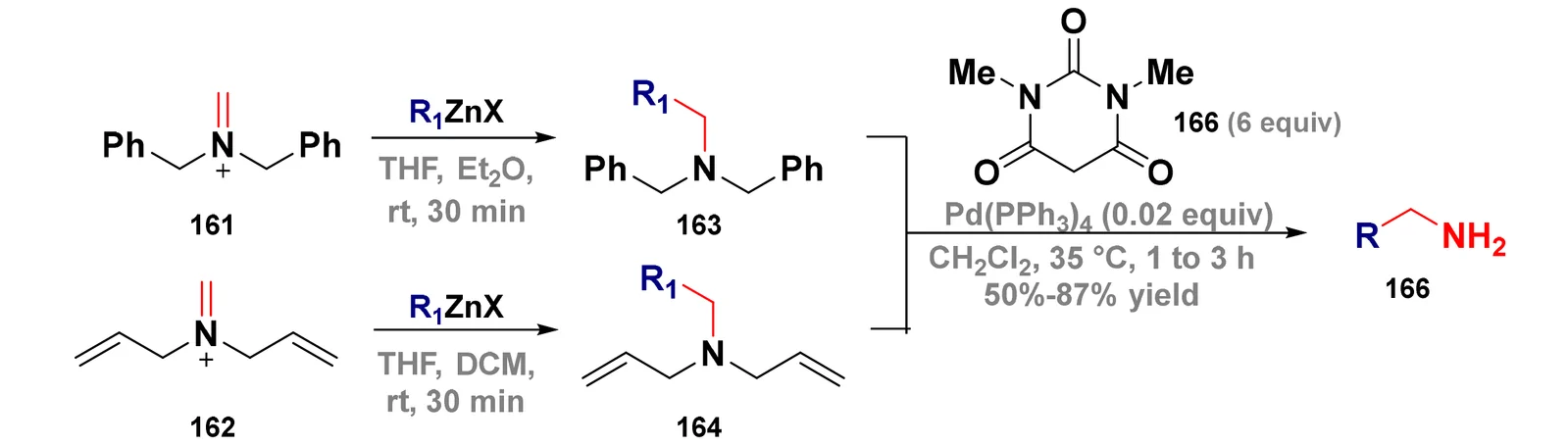
Reactions between iminium salts and a general organozinc reagent followed by Guibè deprotection.

Importantly, the reactivity of Eschenmoser's salt can be extended to a wide variety of functionalized iminium salts. In particular, it is possible to achieve the same reactivity observed with the methaniminium salt by employing salts bearing different substituents. The ability of various iminium salts to exhibit similar reactivity represents an important characteristic of this class of compounds, making them highly versatile and applicable to a broad range of synthetic targets. Indeed, the addition of organozinc to iminium salts 161 and 162 could afford primary amines 165 upon deprotection of the allyl or benzyl moieties^145^ following the Guibé method^146^ (*N*,*N*‘-dimethylbarbituric acid 166 in the presence of Pd(PPh)_3_ in CH_2_Cl_2_) (Scheme 71).

**Scheme 72:**
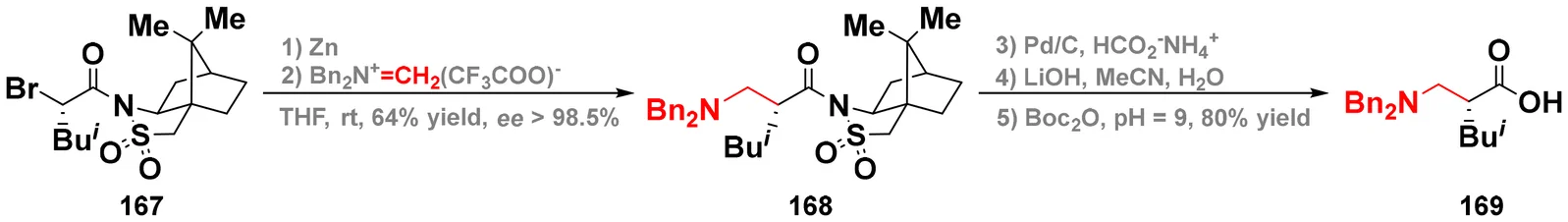
Synthesis of β^2^-homoleucine.

Iminium trifluoroacetates could also engage in a Reformatsky-type reaction as reported by Moumne *et al.*^147^ for the synthesis of β^2^-aminoacids. The Reformatsky reaction of α-bromo esters 167 with iminium trifluoroacetate salts provides a direct method to access β-amino acids 169 (Scheme 72). The asymmetric version of the aminomethylation of the Reformatsky reagent (*via* the use of Oppolzer's sultam as a chiral auxiliary) allowed the synthesis of β^2^-homoleucine, which was employed in the structure–activity relationship studies of biologically active peptides.

## 5. Eschenmoser's salt equivalents

The difficulties associated with the classical preparation and purification of anhydrous, soluble, and functionalized iminium salts can be overcome by generating these reactive intermediates directly in the reaction mixture, offering several practical and mechanistic advantages. In fact, many preformed iminium salts are hygroscopic, thermally unstable, or poorly soluble in common organic solvents, which complicates their isolation and handling. Therefore, generating *in situ* the reactive intermediates circumvents these issues, ensuring greater operational simplicity and often leading to higher overall yields. Furthermore, this approach enables the rapid and controlled formation of the electrophilic species under mild conditions, minimizing decomposition and undesired side reactions.

**Scheme 73:**
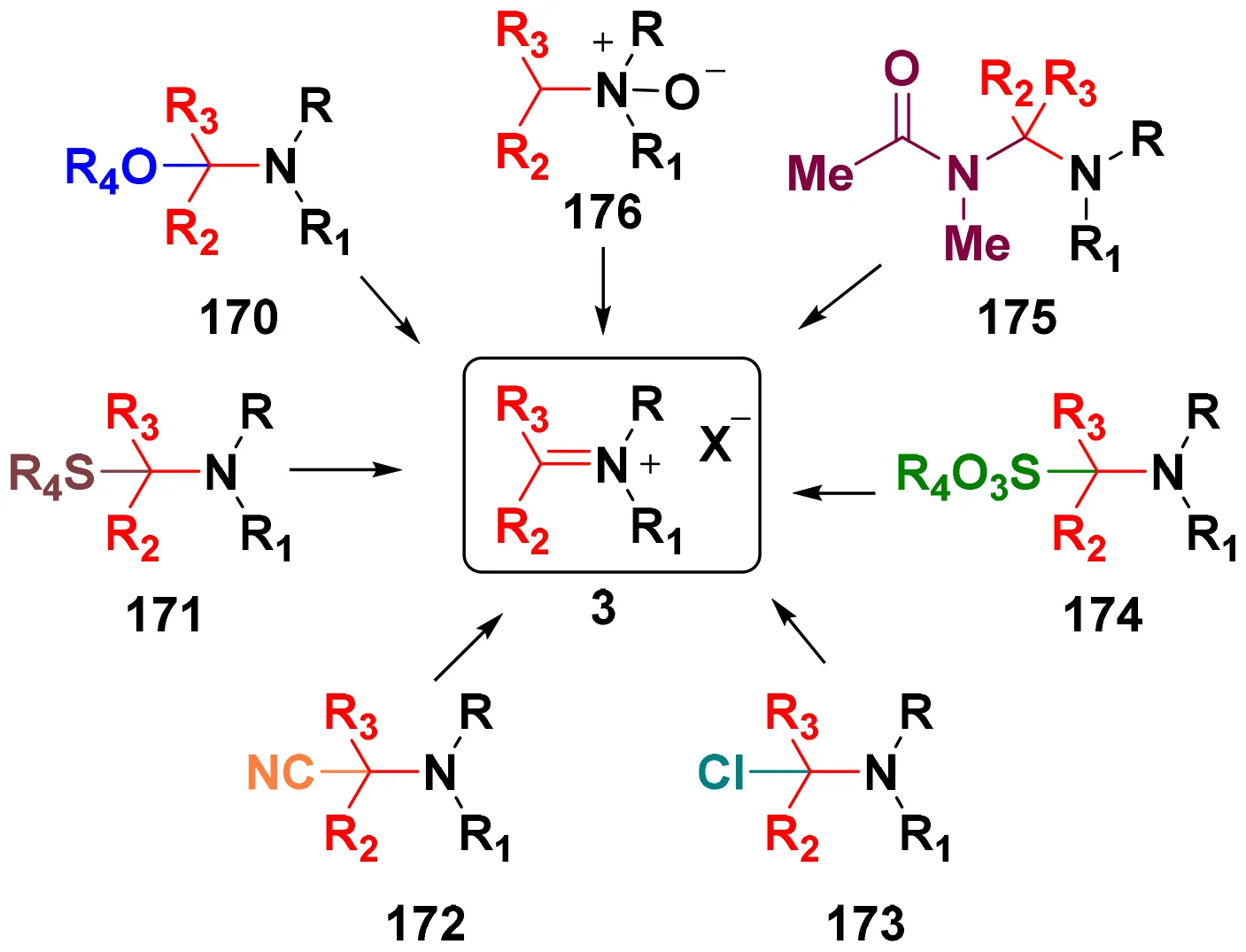
Different synthetic pathways to specific functionalized iminium salts.

The availability of a wide range of *in situ* generated iminium salts has greatly expanded their synthetic potential, for example, in reactions with organometallic reagents. Typically, *N*,*N*-disubstituted aminoalkyl ethers (or acetates) 170, sulfides 171 and nitriles 172 are frequently employed as precursors. In addition, *N*,*N*-disubstituted aminoalkyl amides 173, sulfonates 174, halides 175 and amine *N*-oxides 176 have also been successfully employed for this purpose (Scheme 73).

Overall, the *in situ* generation of functionalized iminium salts enhances both the efficiency and scope of a variety of transformations, allowing the development of more versatile and reliable methodologies for the synthesis of nitrogen-containing compounds. This versatility highlights the central role of iminium salts as key intermediates in modern synthetic chemistry.

**Scheme 74:**
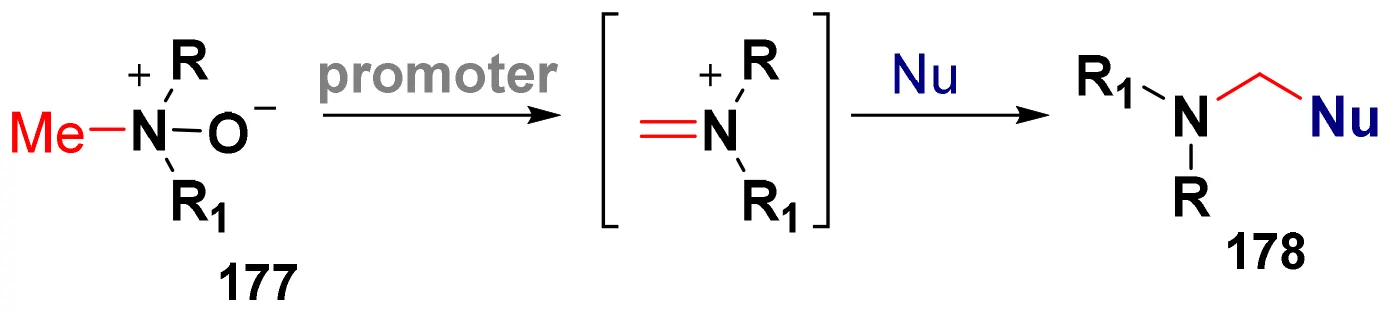
Polonovsky preparation of iminium ions from amine oxide.

Among the several methods available for generating the required iminium ions, a commonly used procedure known as the Polonovsky or modified-Polonovsky reaction employs a tertiary amine *N*-oxide 177 which is treated with a promoter, namely acetic anhydride, TFAA, sulfur dioxide, or ferrous salt; a subsequent treatment with nucleophiles will deliver the corresponding tertiary amines 178.^148^ The process usually requires more than stoichiometric amounts of these expensive promoters which are sometimes difficult to handle (Scheme 74).

**Scheme 75:**
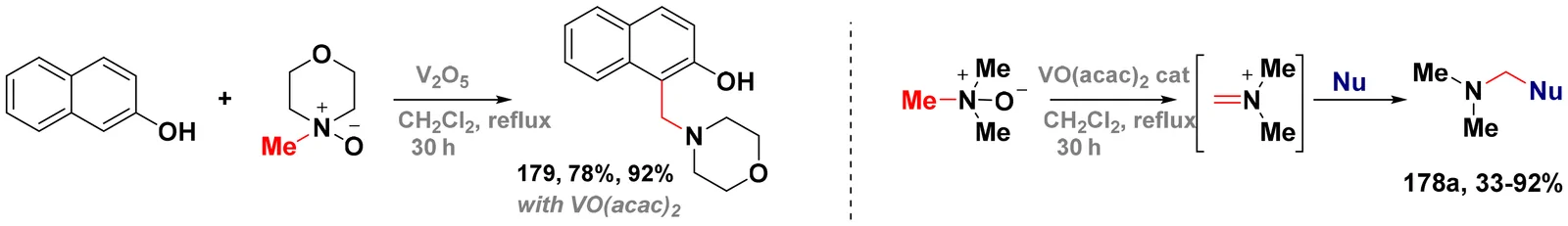
Equivalent reaction to Eschenmoser's methylenation with a vanadium catalyst.

To avoid the need of a promoter, Hwang and Uang^149,150^ proposed a vanadium-mediated generation of the iminium ion from amine oxide. Hence, by reacting an equimolar amount of 2-naphthol and *N*-methylmorpholine-*N*-oxide (NMO) in the presence of a catalytic amount of V_2_O_5_ (10 mol%), the corresponding Mannich adduct 179 was obtained in good yield. In order to develop a more general method, the more soluble VO(acac)_2_ was successfully applied for the conversion of NMO and trimethylamine oxide (TMNO) into iminium salts equivalent to Eschenmoser's salt. Various naphthol and phenolic nucleophiles were reacted under these optimized reaction conditions, providing the corresponding Mannich base 178a in excellent yields (Scheme 75).

The methodology proved to be perfectly versatile also in the case of a variety of tertiary amine oxides and for glycinate *N*-oxides which exhibited high regioselectivity in the formation of Mannich bases. Interestingly, the protocol involves an umpolung-type reaction facilitating the incorporation of an aryl substituent at the α-position of α-amino acids.^150^

**Scheme 76:**
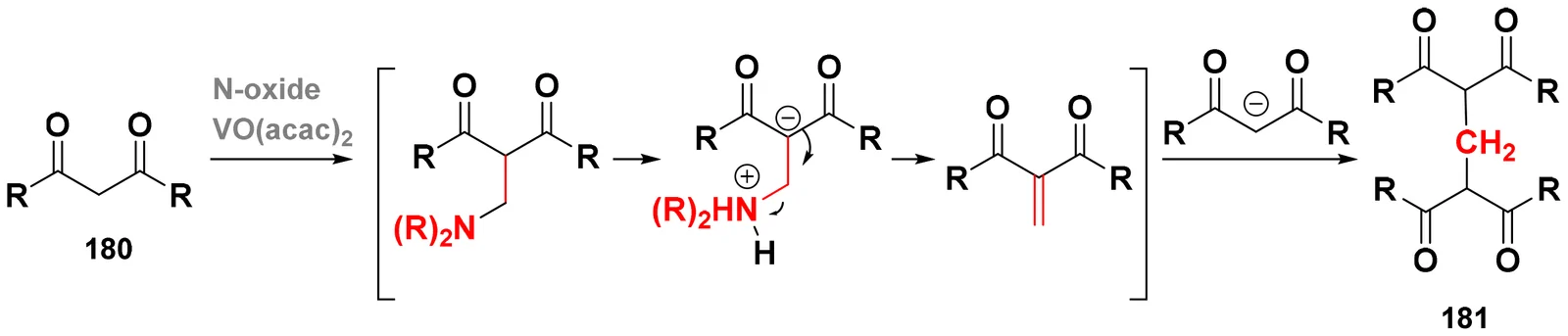
Double alkylation reaction with a vanadium catalyst.

In addition, when 1,3-dicarbonyl compounds 180 were reacted with NMO or TMNO, a double alkylation product 181 was obtained (Scheme 76), due to the subsequent Michael addition of a second molecule of the 1,3-dicarbonyl compound.

**Scheme 77:**
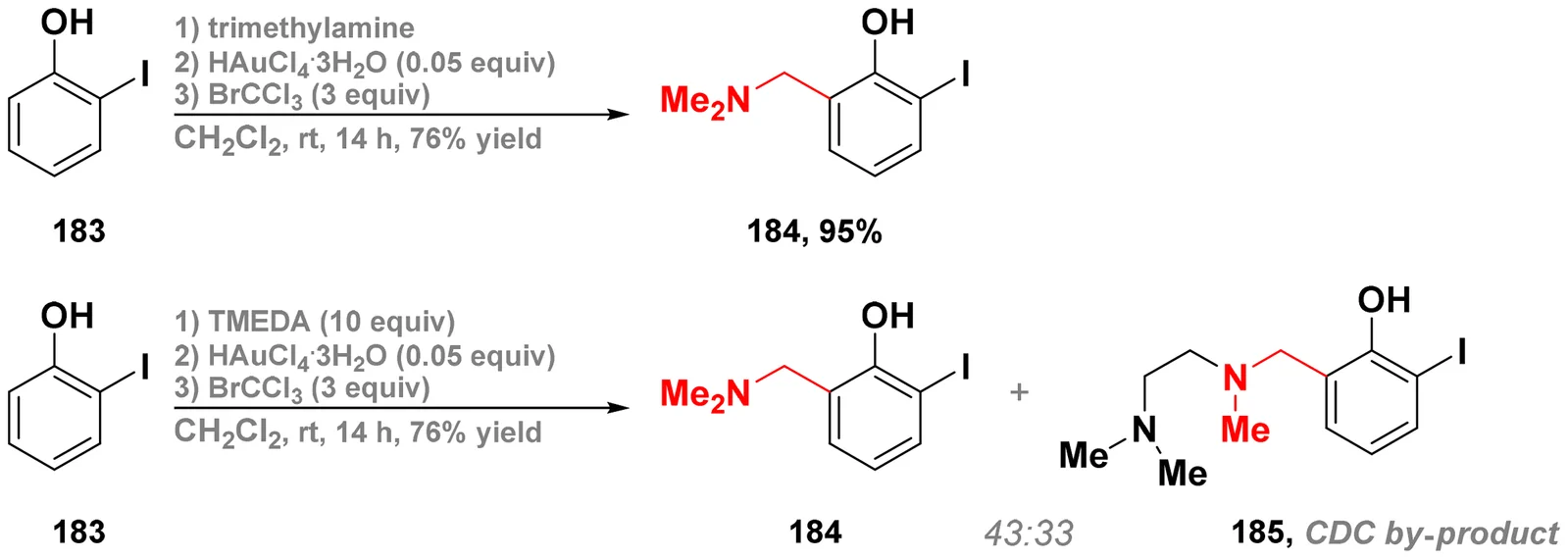
TMEDA as a masked function of Eschenmoser's salt.

The selective alkylation of phenols has attracted considerable attention for many decades. Although Eschenmoser's salt and its derivatives can react with phenols through a Mannich-type reaction, the lack of chemoselectivity remains one of the main drawbacks of these existing methodologies.^151^ To address this limitation, Sun *et al.*^152^ reported in 2013 a visible light promoted Henry-type reaction.^153^ In this transformation, TMEDA (*N*,*N*,*N*′,*N*′-tetramethylethylenediamine) or trimethylamine (TMA) functions as a masked Eschenmoser's salt under oxidative conditions. Remarkably, whereas the direct aminomethylation of phenols 183 under O_2_ oxidative conditions failed to provide satisfactory yields, the employment of a gold catalyst in combination with BrCCl_3_ as an oxidant afforded the desired product 184 in 95% yield (Scheme 77). The reaction proceeded *via* a cross-dehydrogenative coupling (CDC) mechanism when TMA is used; when TMEDA is employed, an alternative carbon–carbon activation coupling (CAC) pathway is initiated, resulting in the same product 184 alongside the CDC by-product 185.

**Scheme 78:**
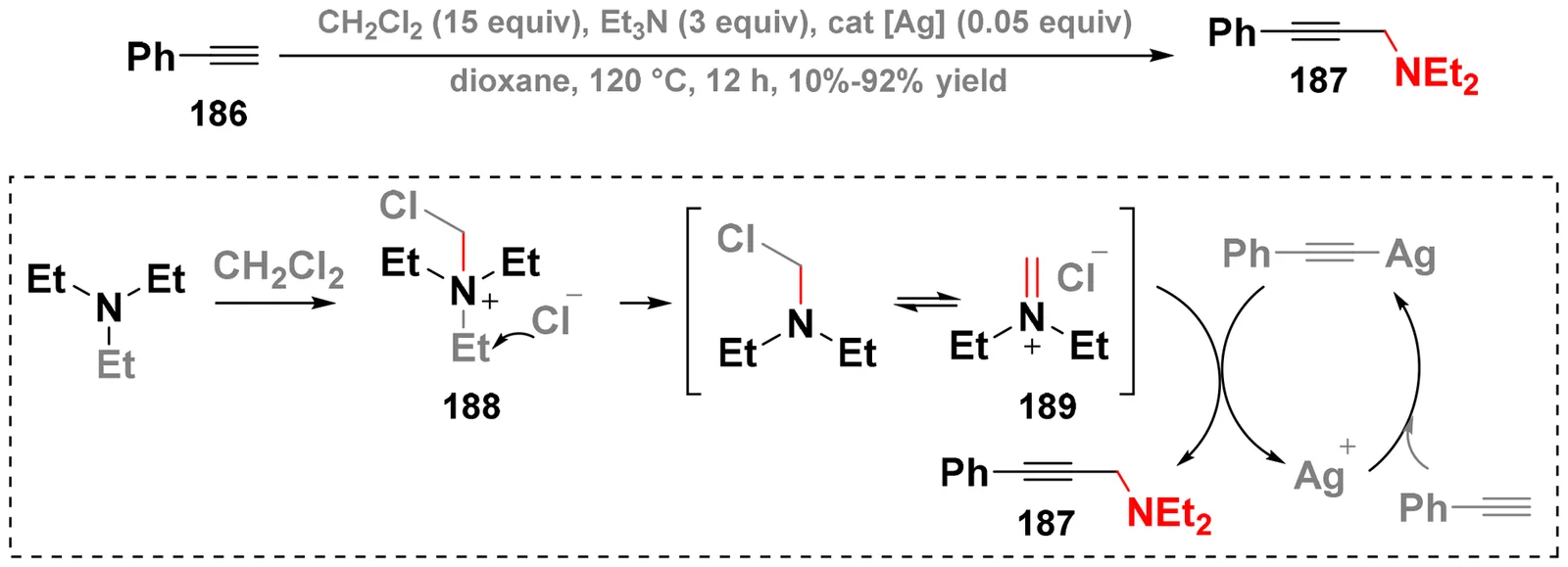
Triethylamine employed as an Eschenmoser's salt equivalent.

In the context of a silver-catalyzed reaction of alkynes such as 186,^154^ CH_2_Cl_2_ and tertiary amines enable the straightforward synthesis of propargylamines 187 in high to excellent yields through selective C–N bond cleavage (Scheme 78). This three-component coupling reaction proceeds *via* the *in situ* formation of a methaniminium chloride 188 from the reaction of the tertiary amine and CH_2_Cl_2_. Subsequent decomposition through R–Cl dissociation generates a methylene ammonium chloride 189, which, upon reacting with the silver catalyst, gives the corresponding propargylamine regenerating the catalyst. This method exhibits remarkable functional group tolerance and can be applied to both aromatic and aliphatic alkynes, as well as a variety of tertiary amines.

**Scheme 79:**
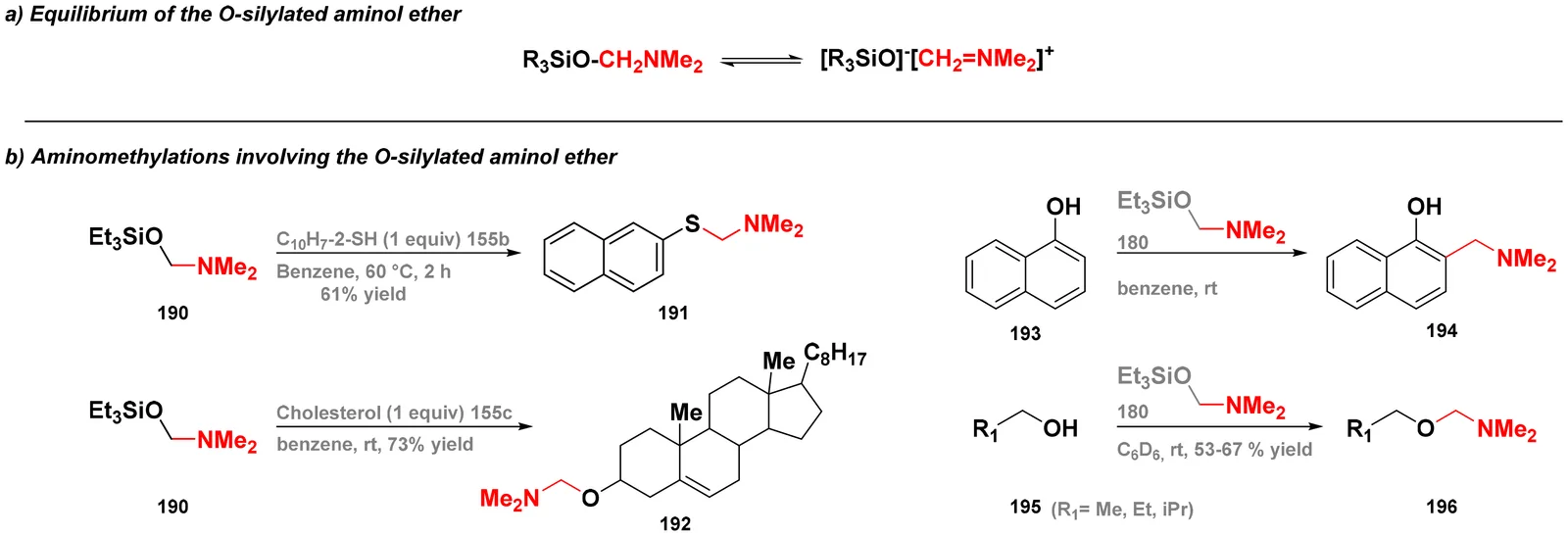
*O*-Silylated aminol ethers as masked analogues of Eschenmoser's salt.

Considering the consistent interest in organosilicon compounds across the chemical sciences, siloxymethyldimethylamines (R_3_SiOCH_2_NMe_2_) represent an intriguing class of reagents that can be considered *masked analogues* of Eschenmoser's salt.^146^ These compounds bear the same aminomethyl unit in a neutral, latent form which can be generated *in situ* and subsequently transferred with the concomitant formation of the thermodynamically favored R_3_SiOH (Scheme 79a). The high stability of the Si–O bond acts as the driving force for the aminomethyl transfer, enabling the process to occur under mild, catalyst-free conditions.

Based on this concept, Gonzalez *et al.*^155^ reported the direct aminomethylation of reactive E–H bonds (E = O, S, N, P, and aromatic C–H) employing *O*-silylated aminol ethers such as 190 for aminomethylation. The reactions proceed almost instantaneously at room temperature, without any need for activating agents, affording the corresponding [(*N*,*N*-dimethylamino)methyl] derivatives such as 191 and 192 in high yields. Notably, for substrates containing E = O, S, or N, no catalysts or activating reagents are required (Scheme 79b). Interestingly, in the case of alcohols, the reactivity pattern depends on the nature of the R group attached to the oxygen: for aromatic alcohols 193, electrophilic substitution on the aromatic ring predominates, yielding the C2-aminomethylated product 194, whereas for aliphatic alcohols 195, substitution occurs preferentially at the hydroxyl site, leading to *O*-aminomethylated compounds 196.

## 6. Conclusions

Eschenmoser's salt and related methaniminium reagents have evolved from classical Mannich-type aminomethylation agents into broadly applicable, fine-tunable electrophilic linchpins that enable both α-methylenation and C–C/C–N bond construction across a remarkably wide substrate scope, from simple carbonyls and active methylene compounds to complex natural products and functional materials. Their performance can be modulated through counterion and structural variation (S1–S3 and analogues), which governs solubility, kinetics, and operational robustness without compromising the characteristic electrophilicity of the C=N center, thereby allowing efficient engagement of enol(ate)s, silyl and boron derivatives, organometallic reagents, and π-systems in highly regio- and stereoselective transformations, including aza-MBH processes and late-stage C–H functionalization. Beyond pre-formed salts, *in situ* generation and “masked” Eschenmoser equivalents—ranging from amine *N*-oxides and tertiary amines under oxidative conditions to *O*-silylated aminol ethers—have expanded the synthetic toolbox by improving handling, enabling catalytic or promoter-economical protocols, and providing access to more sustainable and operationally simple methylenation/aminomethylation manifolds. Collectively, the chemistry surveyed in this review demonstrates that Eschenmoser-type iminium salts are not merely venerable Mannich reagents but versatile, re-designable platforms that continue to intersect with modern trends in synthesis, including MAOS technologies, late-stage diversification, and the construction of functional architectures such as N-heterocyclic olefin amines and porphyrinic sensitizers for DSSCs, ensuring their enduring relevance in contemporary organic chemistry. Taken together, the chemistry surveyed here positions Eschenmoser-type iminium salts not only as mature workhorse reagents but also as adaptable platforms poised to intersect with emerging trends in late-stage functionalization, data-driven reaction design, and the construction of increasingly sophisticated functional architectures in organic and medicinal chemistry.

## Conflicts of interest

There are no conflicts to declare.

## Data Availability

No primary research results, software or code have been included and no new data were generated or analysed as part of this review.
